# Neuro-Nutrition and Exercise Synergy: Exploring the Bioengineering of Cognitive Enhancement and Mental Health Optimization

**DOI:** 10.3390/bioengineering12020208

**Published:** 2025-02-19

**Authors:** Vicente Javier Clemente-Suárez, Alexandra Martín-Rodríguez, Agustín Curiel-Regueros, Alejandro Rubio-Zarapuz, José Francisco Tornero-Aguilera

**Affiliations:** 1 Faculty of Sport Sciences, Universidad Europea de Madrid, Villaviciosa de Odón, 28670 Madrid, Spain; vctxente@yahoo.es (V.J.C.-S.); sandra.martin.rodriguez8@gmail.com (A.M.-R.); agustin.curiel.regueros@universidadeuropea.es (A.C.-R.); 2 Grupo de Investigación en Cultura, Educación y Sociedad, Universidad de la Costa, Barranquilla 080002, Colombia; 3 Faculty of Applied Social Sciences and Communications, UNIE, 28015 Madrid, Spain; 4 KOS Generating Health, 45007 Toledo, Spain

**Keywords:** neuro-nutrition, exercise-induced neuroplasticity, bioengineered interventions, cognitive enhancement, mental health optimization, wearable bioelectronics

## Abstract

The interplay between nutrition, physical activity, and mental health has emerged as a frontier in bioengineering research, offering innovative pathways for enhancing cognitive function and psychological resilience. This review explores the neurobiological mechanisms underlying the synergistic effects of tailored nutritional strategies and exercise interventions on brain health and mental well-being. Key topics include the role of micronutrients and macronutrients in modulating neurogenesis and synaptic plasticity, the impact of exercise-induced myokines and neurotrophins on cognitive enhancement, and the integration of wearable bioelectronics for personalized monitoring and optimization. By bridging the disciplines of nutrition, psychology, and sports science with cutting-edge bioengineering, this review highlights translational opportunities for developing targeted interventions that advance mental health outcomes. These insights are particularly relevant for addressing global challenges such as stress, anxiety, and neurodegenerative diseases. The article concludes with a roadmap for future research, emphasizing the potential of bioengineered solutions to revolutionize preventive and therapeutic strategies in mental health care.

## 1. Introduction

In recent years, the integration of nutrition and exercise as synergistic approaches to enhancing cognitive function and mental health has gained significant attention in scientific and clinical research. The brain, as the most metabolically active organ, relies on precise nutritional inputs and physical activity to support its complex functions, including neurogenesis, synaptic plasticity, and overall neuroprotection. Modern advances in bioengineering offer unprecedented opportunities to investigate and optimize these relationships, enabling the development of personalized interventions to improve psychological resilience, cognitive performance, and quality of life. This emerging interdisciplinary focus addresses critical global challenges, such as the rising prevalence of stress, anxiety disorders, and neurodegenerative diseases. The relationship between physical activity and cognitive function has been widely studied, demonstrating that regular exercise serves as a modifiable lifestyle factor for maintaining or improving cognitive function and preventing cognitive decline and neuro-degenerative diseases [1,2]. Moreover, exercise has been shown to enhance specific cognitive functions and promote neuroplasticity, including increased neuronal growth and activity [3]. In parallel, nutrition plays a critical role in brain health. A balanced diet, rich in omega-3 fatty acids, vitamins, and polyphenols, is essential for neuronal function and mood regulation [4]. The combination of proper nutritional strategies with exercise interventions creates a synergistic effect, amplifying the individual benefits of each. This integrated approach is particularly relevant for addressing conditions such as depression and anxiety, where both nutrition and exercise have shown positive therapeutic effects [5].

The synergy between neuro-nutrition and exercise has emerged as a pivotal area of interest for optimizing brain health and cognitive function. Specific nutrients, such as omega-3 fatty acids, B vitamins, and polyphenols, have been identified as critical for maintaining neuronal integrity and promoting processes like neurogenesis and synaptic plasticity [6]. Omega-3 fatty acids, particularly docosahexaenoic acid (DHA), support synaptic remodeling [referring to the process by which synapses, the connections between neurons, are strengthened, weakened, formed, or eliminated in response to activity, experience, or environmental changes, enabling the brain to adapt, learn, and maintain functional neural circuits] by enhancing membrane fluidity and promoting brain-derived neurotrophic factor (BDNF), a key molecule in neuroplasticity and stress resilience. Polyphenols, on the other hand, exert their effects by activating antioxidant and anti-inflammatory pathways, such as Nrf2 signaling, and modulating kinases like ERK and CREB, which are crucial for synaptic plasticity and cognitive function. Notably, these pathways may converge on shared targets, such as BDNF expression, highlighting potential overlaps and synergistic effects between these nutrients. This nuanced interaction underscores the complementary roles of omega-3 fatty acids and polyphenols in supporting neural health.

Concurrently, exercise is recognized for its ability to stimulate the release of neurotrophic factors [proteins that support the growth, survival, and differentiation of neurons, as well as promote neurogenesis, synaptic plasticity, and repair of the nervous system, playing a critical role in maintaining neural health and function], like BDNF and insulin-like growth factor 1 (IGF-1), which enhance neural connectivity and reduce the risk of cognitive decline [7]. Together, nutrition and exercise potentiate each other’s effects, creating a feedback loop that amplifies neurobiological benefits. This integrative approach provides a promising avenue for addressing age-related cognitive decline, as well as stress-related disorders such as anxiety and depression. Recent findings suggest that combining tailored dietary interventions with structured physical activity can maximize cognitive performance and overall mental health [4,8]. By integrating these mechanisms into preventive and therapeutic strategies, the potential for improving quality of life and cognitive resilience across the lifespan becomes increasingly evident.

Additionally, gender-specific variations in the impact of nutrition and exercise on cognitive performance are increasingly acknowledged, underscoring the necessity of customizing therapies to accommodate these variances [8]. Hormonal fluctuations, including variations in estrogen and testosterone levels, significantly affect cognitive functions and neuroplasticity, shaping the responses of men and women to nutritional and physical exercise interventions. Women may derive more advantages from nutrients such as omega-3 fatty acids and iron due to the requirements of the menstrual cycle, pregnancy, and menopause, whereas men may display distinct metabolic and inflammatory reactions to comparable dietary inputs [8]. The cognitive advantages of exercise, especially aerobic and resistance training, seem to be affected by hormonal and physiological factors, with women exhibiting improved memory and emotional regulation following specific forms of physical activity in comparison to males [9]. The synergistic effects of neuro-nutrition and exercise demonstrate gender-specific dynamics [4]; for instance, women may experience enhanced mood and stress resilience due to the inter-play between exercise-induced brain-derived neurotrophic factor (BDNF) and nutritional regulation [9]. These observations emphasize the necessity for gender-specific techniques in enhancing cognitive function through integrated nutrition and physical activity interventions [3].

The integration of bioengineering into neuro-nutrition and exercise science has significantly advanced the optimization of cognitive function and mental health. Wearable bioelectronics, such as smartwatches and fitness trackers, enable real-time monitoring of physiological parameters, including heart rate variability and physical activity levels, which are crucial for assessing mental well-being [9]. These devices facilitate personalized interventions by providing continuous data that can be analyzed to tailor nutrition and exercise programs to individual needs. For instance, wearable biosensors capable of non-invasive sweat diagnostics can monitor biomarkers related to stress and hydration, offering insights that inform dietary adjustments and exercise regimens [10]. Additionally, bioelectronic advances, such as flexible sensor systems, have improved the detection of physiological changes, allowing for the monitoring of mental health and cognitive load through precise physiological signals [11]. This integration of bioengineering technologies into everyday life enhances the precision of health monitoring and empowers individuals to actively manage their brain health through informed lifestyle choices [12].

The objective of this systematic review is to analyze the synergistic effects of neuro-nutrition and exercise on cognitive enhancement and mental health optimization. Specifically, the review aims to evaluate how key nutrients, such as omega-3 fatty acids, B vitamins, and antioxidants, interact with exercise-induced neurotrophic factors, including BDNF and myokines, to influence neuroplasticity, mood regulation, and stress resilience. Additionally, it seeks to explore the mechanisms through which these interventions impact neurogenesis, synaptic plasticity, and the prevention of neurodegenerative diseases. By synthesizing current evidence, this review aspires to provide a comprehensive understanding of the combined benefits of neuro-nutrition and exercise, offering insights into personalized intervention strategies for optimizing mental health and cognitive performance.

To conduct this systematic review, established methodologies were adhered to, ensuring a rigorous and unbiased approach [13,14,15]. Systematic reviews are instrumental in synthesizing existing evidence, identifying research gaps, and evaluating the quality and reliability of published studies [16,17]. This review methodology focuses on integrating and critically analyzing findings from diverse sources to advance scientific understanding and guide future research directions. The search was conducted across databases including PubMed, Scopus, and Web of Science, focusing on articles published between 1 January 2010 and 1 December 2024, to ensure the inclusion of the most relevant and up-to-date research. Foundational studies predating 2010 were included when necessary to provide historical or contextual insights. The inclusion criteria comprised (i) peer-reviewed studies examining the effects of nutrition and exercise on neuroplasticity, cognitive function, or mental health, (ii) research on key nutrients and exercise-related neurotrophic factors, and (iii) studies conducted in human or animal models with translational relevance. Exclusion criteria included (i) studies unrelated to the review’s objectives, (ii) publications with significant methodological flaws, and (iii) non-peer-reviewed sources, such as these, conference proceedings, and unpublished studies. Articles meeting the inclusion criteria were independently assessed by all authors for their relevance, scientific rigor, and alignment with the review’s subsections. Discrepancies were resolved through consensus to ensure the validity of the findings.

## 2. Neuro-Nutrition: Key Nutrients for Brain Health

As a highly dynamic organ, the brain continuously adapts to new experiences and environmental changes through processes such as neuroplasticity. Neurotrophic factors, including brain-derived neurotrophic factor (BDNF), nerve growth factor (NGF), and insulin-like growth factor 1 (IGF-1), play a crucial role in supporting neuronal growth, survival, and synaptic plasticity. These proteins influence key cognitive functions such as learning and memory by promoting neurogenesis and enhancing neural connectivity. In particular, BDNF activates the TrkB receptor, triggering signaling pathways that regulate synaptic strength and plasticity, contributing to cognitive resilience and mental well-being.

Equally important is synaptic remodeling, a process that allows the brain to optimize neural connections by strengthening or weakening synapses based on activity and experience. This mechanism underlies cognitive flexibility, memory consolidation, and overall brain adaptability. The interaction between neurotrophic factors and synaptic remodeling is fundamental in maintaining cognitive function and preventing age-related decline.

Understanding these mechanisms provides valuable insights for designing nutritional and exercise interventions aimed at optimizing brain health and enhancing mental performance. For instance, allelic variants of the BDNF gene, especially the Val66Met variant, substantially affect cognitive skills like memory, inhibitory control, and neuroplasticity. Individuals possessing the Met allele frequently demonstrate less activity-dependent BDNF release, potentially attenuating the neuroprotective benefits of essential nutrients such as omega-3 fatty acids, polyphenols, and B vitamins [17]. These nutrients, recognized for their ability to augment BDNF expression and mitigate oxidative stress, may exhibit differing efficacy based on an individual’s genetic makeup. Additionally, polymorphisms in monoamine neurotransmitter systems, such the serotonin transporter (5-HTTLPR) and dopamine-related COMT Val158Met polymorphisms, interact with BDNF variations to influence mood regulation and executive function [17]. Tailored nutritional therapies, guided by genomic analysis, offer potential for enhancing cognitive outcomes by addressing specific genetic vulnerabilities, especially in groups predisposed to neurodegenerative disorders or cognitive deterioration [11]. This complex organ, which accounts for about 2% of body weight but consumes roughly 20% of the body’s energy resources, relies heavily on a diverse range of nutrients to maintain its structure, optimize its function, and enhance its resilience to aging and environmental stressors [18]. Nutritional interventions that support brain health have become a focal point in the effort to preserve cognitive function throughout the lifespan and combat the increasing prevalence of neurodegenerative diseases such as Alzheimer’s disease and Parkinson’s disease. Regarding gender disparities, there are markedly influential nutritional requirements for cognitive function. Women require elevated quantities of iron and omega-3 fatty acids owing to reproductive variables, including menstruation and pregnancy, which influence both physical and mental well-being. Moreover, hormonal fluctuations during menopause can modify food intake and use in the female brain. Men, conversely, demonstrate distinct metabolic and inflammatory reactions to comparable foods, which may modify their cognitive advantages. A gender-specific emphasis on neuro-nutrition may improve intervention options for sustaining cognitive resilience in both genders [19].

Thus, epidemiological evidence underscores the importance of nutrition in brain health. Studies have consistently shown that dietary patterns rich in specific nutrients, particularly those found in Mediterranean or plant-based diets, are associated with better cognitive outcomes and a reduced risk of dementia and other cognitive disorders [19,20]. For instance, the Framingham Heart Study, one of the largest and most comprehensive longitudinal studies on aging, has identified a strong link between diet and cognitive decline. Researchers found that individuals with a higher intake of vegetables, fruits, whole grains, and omega-3 fatty acids exhibited better cognitive function and had a lower risk of developing Alzheimer’s disease [21]. Conversely, diets high in saturated fats and refined sugars have been associated with cognitive decline, highlighting the negative impact of poor nutrition on brain health [22]. Moreover, large-scale cohort studies such as the Rotterdam Study have further illustrated the role of micronutrients in protecting the brain. This study identified a direct relationship between vitamin D levels and cognitive function, suggesting that deficiencies in this vital nutrient are associated with increased cognitive decline in older adults [23]. Similarly, research conducted as part of the Nurses’ Health Study has shown that adequate intake of B vitamins, particularly folate, B6, and B12, is essential for cognitive health, as these vitamins play a role in homocysteine metabolism, a process that affects neuronal health and mental function [23].

In addition to cognitive decline, nutrition also plays a significant role in mental health. Epidemiological studies have demonstrated that nutritional factors can influence the risk of developing mood disorders such as depression and anxiety. A systematic review and meta-analysis of cohort studies found that individuals with higher intakes of fruits, vegetables, fish, and nuts have a lower risk of depression, while those consuming diets high in processed foods, refined carbohydrates, and fats have a higher incidence of mental health disorders [24]. These findings suggest that nutrition not only impacts cognitive function but also plays a critical role in maintaining emotional balance and mental well-being. The concept of neuro-nutrition has emerged as a promising field dedicated to exploring how specific nutrients affect brain structure, function, and mental health. It recognizes that the brain, like other organs, requires a continuous supply of bioactive compounds, ranging from macronutrients such as omega-3 fatty acids to micronutrients like vitamins, minerals, and polyphenols to support optimal neuronal function. These nutrients influence critical processes such as neurogenesis, synaptic plasticity, and neurotransmitter regulation. Understanding how these nutrients interact with brain cells can inform therapeutic strategies aimed at improving cognitive performance and mental health, particularly in aging populations and those suffering from neurodegenerative diseases or mental health conditions like depression.

### 2.1. The Role of Omega-3 Fatty Acids in Brain Health

Omega-3 fatty acids, particularly docosahexaenoic acid (DHA) and eicosapentaenoic acid (EPA), have garnered significant attention due to their profound impact on brain health. These polyunsaturated fatty acids are considered essential for optimal brain function and development, as they are integral to neuronal structure, synaptic function, and cognitive performance. DHA and EPA are critical components of the phospholipid bilayer that forms neuronal cell membranes, contributing to membrane fluidity and structural integrity. Their role in maintaining cellular homeostasis, modulating inflammation, and supporting neuroplasticity make them indispensable for both the development and aging of the brain.

Omega-3 fatty acids, particularly DHA, play a central role in the fluidity and functionality of neuronal membranes. DHA, being the most abundant omega-3 fatty acid in the brain, is highly concentrated in areas of the brain associated with cognitive functions, including the hippocampus, prefrontal cortex, and cerebral cortex [25]. These regions are directly involved in memory, executive functions, and decision making. The ability of DHA to modulate the physical properties of neuronal membranes enhances synaptic plasticity, a critical feature of learning and memory [6]. DHA is crucial for the structure and function of synapses, the points of communication between neurons. Research has shown that DHA is involved in the formation of synaptic vesicles and the proper functioning of neurotransmitter receptors, such as glutamate and gamma-aminobutyric acid (GABA) receptors [26]. This membrane fluidity is vital for maintaining effective synaptic transmission, which is necessary for processing and transmitting information across neural circuits. Therefore, a deficiency in DHA may impair synaptic function, leading to cognitive deficits and susceptibility to neurodegenerative diseases.

The neuroprotective effects of omega-3 fatty acids are well documented in both preclinical and clinical studies. In clinical trials, omega-3 supplementation has demonstrated the ability to enhance cognitive performance, particularly in aging populations. For instance, a randomized controlled trial demonstrated significant improvements in cognitive performance in older adults who supplemented with omega-3 fatty acids [27]. The study showed improvements in memory, attention, and executive function, underscoring the potential of omega-3 supplementation to mitigate age-related cognitive decline. In addition, it has been found that omega-3 fatty acids, particularly DHA and EPA, could lower the risk of cognitive decline and reduce symptoms of mood disorders, such as depression, in elderly individuals [28]. The ability of omega-3 fatty acids to promote cognitive health is attributed to their influence on neuroinflammation, neurogenesis, and synaptic plasticity. Omega-3s have been shown to increase the production of BDNF, a protein that plays a crucial role in the survival and growth of neurons, as well as in synaptic plasticity [29]. This neurotrophic effect is especially important in the context of neurodegenerative diseases, as BDNF supports neuronal resilience and repair.

One of the key mechanisms through which omega-3 fatty acids exert their neuroprotective effects is by modulating neuroinflammation. Chronic neuroinflammation is a hallmark of neurodegenerative diseases such as Alzheimer’s disease, Parkinson’s disease, and multiple sclerosis, and it is thought to accelerate neuronal damage. Omega-3 fatty acids, particularly DHA, have been shown to reduce the activity of pro-inflammatory cytokines, such as tumor necrosis factor-alpha (TNF-α) and interleukin-1 beta (IL-1β), which are elevated in neurodegenerative conditions [30]. By inhibiting the production of these cytokines, omega-3s can help attenuate the inflammatory response, thereby reducing neuronal injury and protecting cognitive function. Furthermore, omega-3 fatty acids have been shown to decrease oxidative stress, a major contributor to neurodegeneration. Oxidative stress results from an imbalance between the production of reactive oxygen species (ROS) and the brain’s ability to neutralize them with antioxidants. Omega-3 supplementation has been associated with reduced oxidative damage in the brain, as DHA and EPA possess inherent antioxidant properties that help to scavenge free radicals and prevent cellular damage [31].

Omega-3 fatty acids, especially DHA, also support neurogenesis, the process by which new neurons are generated, particularly in the hippocampus, an area of the brain critical for learning and memory. The ability to promote neurogenesis is closely linked to the role of omega-3s in synaptic plasticity, which enables the brain to adapt to new experiences and information. In preclinical studies, omega-3 supplementation has been shown to increase the number of neurons in the hippocampus, which is associated with improved learning and memory [32]. Furthermore, DHA has been shown to modulate the expression of genes related to neurogenesis, such as BDNF, which plays a critical role in the survival and maturation of new neurons [29]. By enhancing neurogenesis and synaptic plasticity, omega-3s contribute to cognitive resilience and the brain’s capacity to adapt to environmental changes or injuries, providing a potential therapeutic approach for conditions characterized by cognitive decline.

The protective effects of omega-3 fatty acids extend beyond cognitive enhancement and play a significant role in preventing age-related cognitive decline and neurodegenerative diseases. Studies have shown that individuals with higher levels of omega-3s in their diet or blood have a lower risk of developing Alzheimer’s disease and other forms of dementia. The Rush Memory and Aging Project found that higher omega-3 intake was associated with a slower rate of cognitive decline and a lower risk of dementia in elderly individuals [20]. Additionally, a systematic review concluded that omega-3 fatty acids are beneficial in slowing the progression of cognitive decline, particularly in individuals at risk of Alzheimer’s disease [32]. Omega-3 fatty acids’ ability to reduce neuroinflammation, enhance neurogenesis, and promote synaptic plasticity makes them a powerful tool in the fight against neurodegenerative diseases. In clinical trials, DHA and EPA supplementation has shown promise in improving cognitive function in individuals with mild cognitive impairment (MCI), a precursor to Alzheimer’s disease. Moreover, DHA supplementation improved cognitive performance in patients with MCI, providing evidence for its potential use as a preventive treatment for Alzheimer’s disease [33].

### 2.2. B Vitamins and Their Critical Role in Brain Health

B vitamins, particularly folate, vitamin B6 (pyridoxine), and vitamin B12 (cobalamin), are integral to the proper functioning of the brain, playing essential roles in several biochemical processes that support cognitive function, mood regulation, and neuroprotection. These vitamins are involved in critical processes such as methylation, neurotransmitter synthesis, and DNA repair, all of which are vital for maintaining brain health and reducing the risk of neurodegenerative diseases and cognitive decline.

Vitamin B12 and folate are both crucial for the synthesis and repair of DNA, particularly in rapidly dividing cells, including neurons. Deficiencies in these vitamins can lead to significant neurological and cognitive impairments. A deficiency in vitamin B12, for example, can lead to demyelination of neurons, resulting in cognitive deficits, mood disturbances, and, in severe cases, irreversible brain damage [34]. Folate, similarly, is essential for the synthesis of neurotransmitters such as serotonin, dopamine, and norepinephrine, which regulate mood, behavior, and cognitive function. Research has shown that low levels of B12 and folate are associated with an increased risk of neurodegenerative diseases, including Alzheimer’s disease. Further on, low levels of these vitamins were significantly correlated with an increased risk of Alzheimer’s disease, underscoring the importance of maintaining optimal levels of these nutrients for long-term cognitive health [35]. Furthermore, elevated levels of homocysteine, a byproduct of methionine metabolism, are considered a major risk factor for cognitive decline and neurodegenerative diseases, particularly when vitamin B12 and folate levels are insufficient to properly convert homocysteine into methionine. High homocysteine levels are associated with vascular damage, neuroinflammation, and synaptic dysfunction, all of which contribute to cognitive decline [36]. Both folate and vitamin B12 work to regulate homocysteine levels, and their adequate intake is essential for the prevention of cognitive impairment and related neurological conditions.

Vitamin B6, another crucial member of the B-vitamin family, plays a pivotal role in the synthesis of neurotransmitters like GABA, dopamine, and serotonin. These neurotransmitters are involved in regulating mood, cognition, and sleep, and deficiencies in vitamin B6 have been linked to cognitive deficits, mood disturbances, and even psychiatric disorders such as depression [37]. Research has shown that supplementation with vitamin B6 can improve mood and reduce the severity of symptoms in individuals with depression, suggesting its potential as a therapeutic intervention for mood disorders [36]. Additionally, vitamin B6 is involved in the regulation of homocysteine levels, further supporting its neuroprotective effects in maintaining brain health. In addition to their roles in neurotransmitter synthesis and homocysteine regulation, B vitamins are also critical for reducing oxidative stress and inflammation, which are central to the aging process and the development of neurodegenerative diseases like Alzheimer’s, Parkinson’s, and Huntington’s disease. As people age, oxidative stress increases due to an imbalance between ROS and the body’s antioxidant defenses. This oxidative damage can accelerate neuronal degeneration and exacerbate cognitive decline. Studies have shown that B-vitamin supplementation, particularly of folate, B6, and B12, can lower oxidative stress markers in the brain, providing a neuroprotective effect [38]. Also, B-vitamin supplementation could reduce oxidative stress and improve cognitive function in elderly populations, further supporting the need for adequate B-vitamin intake throughout life to protect against age-related cognitive decline [39].

Additionally, the potential for B-vitamin supplementation to support brain health extends beyond just their neuroprotective properties. Studies have shown that B-vitamins, particularly when consumed together, work synergistically to improve cognitive function. For example, B-complex vitamins, which include B1 (thiamine), B2 (riboflavin), B3 (niacin), and B5 (pantothenic acid) in addition to B6, B12, and folate, have been found to improve cognitive performance, memory, and attention in both young adults and the elderly [40]. The combination of B-vitamins helps in regulating energy production within the brain and supporting overall neural health, further emphasizing their importance as a dietary cornerstone for maintaining brain function. In conclusion, B vitamins play a critical and multifaceted role in maintaining brain health. From supporting DNA synthesis and neurotransmitter production to regulating homocysteine levels and reducing oxidative stress, these vitamins are essential for cognitive function, emotional well-being, and the prevention of neurodegenerative diseases. Given the growing body of evidence linking B-vitamin deficiency with cognitive decline, mood disorders, and age-related neurological diseases, ensuring adequate intake of these vitamins through diet or supplementation is essential for optimizing brain health across the lifespan.

### 2.3. Antioxidants and Neuroprotection: The Role of Vitamins C and E

Oxidative stress plays a significant role in the aging of the brain and the development of numerous neurological disorders. The brain’s high metabolic activity makes it particularly susceptible to oxidative damage, as the process of cellular respiration generates free radicals that can damage cellular structures, including lipids, proteins, and DNA. This damage is linked to the progression of various neurodegenerative diseases, such as Alzheimer’s disease, Parkinson’s disease, and Huntington’s disease. Antioxidants, which neutralize free radicals, are essential for maintaining brain health and protecting neurons from oxidative damage. Among the most crucial antioxidants are vitamins C and E, both of which have well-documented neuroprotective properties.

Vitamin C, also known as ascorbic acid, is a potent water-soluble antioxidant that plays a critical role in protecting the brain from oxidative damage. As a scavenger of free radicals, vitamin C neutralizes ROS and prevents cellular damage in brain tissue [41]. One of the most notable functions of vitamin C is its ability to regenerate other antioxidants, such as vitamin E, ensuring a sustained antioxidant defense in brain cells. Additionally, vitamin C is involved in the modulation of neurotransmitter function. It is required for the synthesis of dopamine, a neurotransmitter involved in mood regulation and motor control, by aiding the conversion of tyrosine to dopamine [41]. Research has consistently shown that vitamin C supplementation can reduce markers of oxidative stress and improve cognitive function in individuals with neurodegenerative diseases. In addition, vitamin C supplementation in patients with Alzheimer’s disease led to significant reductions in oxidative stress markers and cognitive decline [38]. In another study, it was found that higher plasma vitamin C levels were associated with a lower risk of cognitive decline in older adults [34]. This underscores the importance of adequate vitamin C intake in protecting against age-related cognitive decline and in supporting overall brain function. Vitamin C also has a role in reducing neuroinflammation, which is often elevated in neurodegenerative diseases. By neutralizing ROS and reducing inflammatory mediators, vitamin C contributes to a reduction in brain inflammation, which is linked to cognitive dysfunction and neurodegeneration. Therefore, vitamin C is not only essential for protecting neurons from oxidative damage but also for promoting a healthy inflammatory response in the brain.

Vitamin E, a fat-soluble antioxidant, plays a critical role in the maintenance of neuronal health, particularly in protecting neuronal membranes from lipid peroxidation. Lipid peroxidation, the oxidative degradation of lipids, results in the formation of reactive aldehydes and other byproducts that can damage neuronal membranes and disrupt cellular functions. This process is particularly damaging in the brain, where phospholipid-rich neuronal membranes are essential for maintaining synaptic integrity and function. Vitamin E acts by scavenging free radicals and preventing the oxidation of polyunsaturated fatty acids in neuronal membranes, thus preserving the integrity of the brain’s cellular structures [33]. The neuroprotective effects of vitamin E are particularly evident in the context of aging and neurodegenerative diseases. Vitamin E supplementation has been shown to slow the progression of Alzheimer’s disease in elderly patients, highlighting its potential as a therapeutic agent for neurodegenerative conditions [33]. Furthermore, vitamin E has been shown to reduce the formation of amyloid plaques, which are characteristic of Alzheimer’s disease, by modulating oxidative stress pathways. This suggests that vitamin E not only prevents neuronal damage but may also inhibit the pathological processes that contribute to the onset and progression of Alzheimer’s disease. In addition to its antioxidant properties, vitamin E plays a role in modulating neuroinflammation and supporting neurogenesis. By reducing oxidative stress and inflammation, vitamin E contributes to the preservation of brain plasticity, which is vital for learning, memory, and recovery from brain injury. The ability of vitamin E to support neurogenesis is particularly important in the context of aging, as the brain’s capacity to generate new neurons declines over time. Vitamin E’s effects on neurogenesis and its ability to protect neuronal cells from oxidative stress make it an essential nutrient for maintaining cognitive function throughout life.

Thus, vitamins C and E are critical for brain health due to their powerful antioxidant properties. They work synergistically to protect the brain from oxidative damage, modulate neuroinflammation, and preserve neuronal integrity. Vitamin C protects brain cells by neutralizing free radicals, regenerating other antioxidants like vitamin E, and supporting neurotransmitter synthesis. On the other hand, vitamin E plays a vital role in protecting neuronal membranes from lipid peroxidation, reducing oxidative damage, and promoting neurogenesis. Together, these vitamins contribute to the maintenance of brain plasticity, cognitive function, and mental health, making them indispensable in preventing age-related cognitive decline and neurodegenerative diseases.

### 2.4. Polyphenols and Cognitive Function

Polyphenols, abundant in fruits, vegetables, tea, and dark chocolate, are well-established for their potent antioxidant and anti-inflammatory effects. These bioactive compounds have demonstrated significant benefits for brain health, particularly in supporting cognitive function, neuroprotection, and reducing the risk of age-related cognitive decline. As powerful modulators of oxidative stress and inflammation, polyphenols play a crucial role in protecting the brain from damage caused by aging and neurodegenerative diseases, while also enhancing cognitive abilities through several key mechanisms.

Among the most well-known polyphenolic compounds are flavonoids, which are particularly abundant in blueberries, green tea, and other fruits and vegetables. Flavonoids, such as anthocyanins, the pigments responsible for the red, blue, and purple colors in berries, have shown robust neuroprotective properties. Along these lines, anthocyanins significantly improve memory and cognitive function, with regular consumption of berries associated with enhanced cognitive performance in both younger and older adults [42]. Furthermore, anthocyanins have been shown to support brain plasticity, which refers to the brain’s ability to adapt, reorganize, and form new neural connections in response to learning and environmental changes [43]. This is particularly important in aging populations, where cognitive decline is often accompanied by reduced synaptic function. The neuroprotective effects of flavonoids are attributed to their ability to reduce oxidative stress and inflammation, two major contributors to cognitive aging and neurodegenerative diseases. Berries and other flavonoid-rich foods enhance synaptic plasticity by promoting the production of BDNF, a key protein involved in neuronal survival and synaptic function. BDNF has been shown to facilitate the growth and differentiation of new neurons in the hippocampus, a region critical for memory and learning. By increasing the availability of BDNF and supporting synaptic function, flavonoids like anthocyanins not only protect neurons from damage but also stimulate cognitive processes that contribute to improved memory and cognitive aging [43]. This makes flavonoids particularly effective in slowing cognitive decline and enhancing cognitive function, especially in elderly individuals.

Another important polyphenol in the context of cognitive function is epigallocatechin gallate (EGCG), a major catechin found in green tea. EGCG has been widely studied for its ability to improve cognitive function, reduce neuroinflammation, and protect against neuronal damage, particularly in the context of neurodegenerative diseases such as Alzheimer’s and Parkinson’s. EGCG supplementation improves cognitive performance in animal models by reducing oxidative stress and enhancing memory [44]. The neuroprotective properties of EGCG are primarily attributed to its antioxidant capabilities, which help mitigate the damage caused by free radicals and ROS, which are overproduced in neurodegenerative conditions. EGCG also plays a significant role in modulating neuroinflammation, which is a central mechanism in the development of several neurological diseases. Chronic neuroinflammation can accelerate neuronal degeneration and is associated with the progression of Alzheimer’s, Parkinson’s, and other neurodegenerative diseases. EGCG has been shown to inhibit the activation of pro-inflammatory cytokines and reduce the activation of microglia, the immune cells of the central nervous system, thereby reducing neuroinflammation and protecting neurons from damage [42]. Moreover, EGCG has been found to protect against the accumulation of toxic protein aggregates, such as beta-amyloid plaques and alpha-synuclein fibrils, which are hallmark features of Alzheimer’s and Parkinson’s diseases, respectively. It has been demonstrated that EGCG could inhibit the aggregation of beta-amyloid and alpha-synuclein, potentially reducing the formation of neurotoxic plaques and fibrils that contribute to the progression of these diseases. By preventing the formation of these aggregates, EGCG offers a promising therapeutic strategy for mitigating the development and progression of Alzheimer’s, Parkinson’s, and other neurodegenerative conditions [42].

### 2.5. Amino Acids: Building Blocks for Neurotransmitter Synthesis

Amino acids, as the fundamental building blocks of proteins, play a crucial role in maintaining brain health, particularly in the synthesis of neurotransmitters. These compounds are involved in numerous physiological processes within the brain, including the regulation of mood, cognition, and overall mental well-being. Of particular importance are tryptophan and tyrosine, which are essential amino acids that serve as precursors to critical neurotransmitters such as serotonin and dopamine. Both neurotransmitters are involved in the regulation of mood, sleep, attention, and motivation, making these amino acids essential for cognitive function and emotional stability.

Tryptophan is an essential amino acid and the precursor to serotonin, a neurotransmitter that plays a central role in regulating mood, sleep, appetite, and overall emotional well-being. Serotonin is often referred to as the “feel-good” neurotransmitter due to its involvement in promoting feelings of happiness and contentment. Low serotonin levels are commonly linked to mood disorders such as depression, anxiety, and insomnia [45]. Therefore, maintaining adequate tryptophan levels is crucial for optimal serotonin production, which in turn supports mental health and emotional regulation. Research has demonstrated that tryptophan supplementation can improve mood and alleviate symptoms of depression by increasing serotonin levels in the brain. For instance, a study showed that tryptophan supplementation led to a significant improvement in mood and reduced symptoms of depression in individuals suffering from major depressive disorder [46]. This finding suggests that tryptophan plays a direct role in enhancing serotonin production and, consequently, in improving emotional well-being. Moreover, studies have indicated that tryptophan supplementation can be an effective adjunct treatment for conditions such as seasonal affective disorder (SAD) and generalized anxiety disorder, where serotonin imbalances are often observed. In addition to its impact on mood regulation, serotonin is also involved in the regulation of other critical processes, including sleep and appetite. Because tryptophan is a precursor to serotonin, maintaining an adequate intake of this amino acid is essential for supporting both sleep quality and appetite control. Low serotonin levels can lead to disturbances in sleep patterns, contributing to insomnia or poor sleep quality, which in turn affects overall cognitive performance. Thus, tryptophan not only supports mood but also contributes to broader aspects of mental and physiological health.

Tyrosine, another essential amino acid, is a precursor to dopamine, a neurotransmitter that plays a critical role in motivation, attention, and reward processing. Dopamine is involved in regulating the brain’s reward system and is essential for goal-directed behavior, focus, and motivation. Low dopamine levels are associated with cognitive impairments, lack of motivation, and conditions such as attention deficit hyperactivity disorder (ADHD), Parkinson’s disease, and chronic fatigue syndrome [47]. Therefore, ensuring adequate tyrosine availability is important for maintaining optimal dopamine synthesis and overall cognitive function. Research has shown that tyrosine supplementation can improve cognitive performance, particularly under conditions of stress and fatigue. Tyrosine supplementation enhances memory and attention during cognitively demanding tasks, suggesting that it plays a role in maintaining mental performance under challenging conditions [46]. This is particularly valuable in environments that require sustained cognitive effort, such as studying, working under pressure, or engaging in tasks requiring long-term focus. In stressful or fatiguing situations, the brain’s supply of dopamine can be depleted, leading to cognitive fatigue. Tyrosine supplementation helps replenish dopamine levels, thereby enhancing cognitive resilience and mental performance. Moreover, tyrosine has been shown to improve attention and mental clarity in individuals experiencing stress-induced cognitive decline. Studies have demonstrated that tyrosine supplementation could counteract the cognitive impairments associated with acute stress by restoring dopamine levels and promoting neurochemical balance. This makes tyrosine an important nutrient for supporting mental function in high-stress environments or situations that require intense concentration and focus [47].

## 3. The Role of Exercise in Cognitive Enhancement

Exercise, often lauded for its physical health benefits, plays a significant role in brain health and cognitive enhancement. Physical activity has a profound impact on various cognitive domains, including memory, attention, executive function, and processing speed. The relationship between physical activity and cognitive function extends across the lifespan, showing benefits for young adults, aging populations, and even those at risk for neurodegenerative diseases. The underlying mechanisms by which exercise enhances cognitive performance involve complex physiological changes at the neurochemical, neurogenetic, and structural levels, contributing to the brain’s resilience and adaptability [48]. Additionally, the cognitive advantages of exercise differ between males and females owing to physiological and hormonal disparities. Research indicates that women may exhibit improved memory and emotional management after engaging in aerobic and weight training. Conversely, men may demonstrate more rapid enhancements in muscle-related cognitive skills, such as reaction quickness, yet exhibit distinct responses to stress regulation. Exercise-induced BDNF release, essential for neuroplasticity, is influenced by gender-specific hormonal levels. Tailoring exercise programs according to gender may enhance cognitive function and psychological health in both groups [49]. Also, the cognitive benefits of exercise are also modulated by genetic factors, including allelic variations in BDNF and monoamine neurotransmitter systems. For example, carriers of the Met allele of the BDNF gene may experience attenuated increases in exercise-induced BDNF levels, which are critical for neuroplasticity, memory, and stress resilience [49,50]. Despite this, regular aerobic and resistance exercise has been shown to enhance cognitive function in these individuals, particularly when combined with tailored interventions that account for their genetic predispositions. Additionally, polymorphisms in serotonin and dopamine systems influence the efficacy of exercise in improving inhibitory control, emotional regulation, and executive function. Understanding these genetic interactions underscores the potential for personalized exercise regimens to maximize cognitive benefits, especially in individuals with genetic profiles linked to lower baseline neuroplasticity. Future research should focus on how these insights can inform precision strategies for preventing neurodegenerative diseases and enhancing mental health [51,52].

### 3.1. Exercise and Brain Plasticity

One of the primary mechanisms through which exercise enhances cognitive function is by promoting brain plasticity. Brain plasticity, or neuroplasticity, refers to the brain’s remarkable ability to reorganize itself by forming new neural connections and adapting to new information, experiences, or environmental changes. This dynamic process is essential for various cognitive functions, including learning, memory, and adaptation following brain injury or neurodegenerative diseases. Regular physical activity has been shown to stimulate neural plasticity by promoting cellular processes that enhance both structural and functional brain connectivity. These changes are particularly crucial in maintaining and improving cognitive function throughout life, especially as individuals age or experience cognitive impairment [6].

One of the most significant ways exercise supports brain plasticity is through the release of BDNF, a protein critical for neurogenesis, synaptic plasticity, and the survival of neurons. BDNF has been described as one of the most potent factors influencing brain health because of its role in supporting the growth, differentiation, and survival of neurons. Regular exercise, particularly aerobic exercise, has been shown to increase BDNF levels, which is vital for enhancing the formation of new neurons and strengthening neural networks. This increase in BDNF levels occurs most notably in the hippocampus, a brain region central to memory formation, learning, and spatial navigation. The hippocampus is highly sensitive to exercise-induced changes in BDNF levels, which explains why physical activity has such a profound effect on learning and memory. Studies have consistently shown that exercise-induced increases in BDNF are associated with improvements in both short-term and long-term memory, helping counteract the cognitive decline that accompanies aging [49]. For example, in individuals over the age of 60, regular physical activity has been linked to a slower rate of cognitive decline, with improved recall, verbal fluency, and spatial memory. Exercise not only helps preserve memory function but can also stimulate recovery in individuals with early cognitive impairments. These findings suggest that exercise could serve as an important intervention for preventing or delaying the onset of neurodegenerative diseases, such as Alzheimer’s disease. Furthermore, BDNF’s pivotal role in synaptic plasticity means that exercise not only supports the formation of new neurons but also enhances the efficiency and strength of existing synaptic connections. Synaptic plasticity refers to the process by which synapses, the connections between neurons, become stronger or weaker in response to activity. The ability of synapses to adjust in response to experience and activity is essential for the brain’s capacity to learn and retain new information. Exercise plays a crucial role in enhancing this plasticity by promoting the release of neurotransmitters and signaling molecules that facilitate synaptic changes, improving memory consolidation and cognitive flexibility.

Through this process, exercise supports the structural and functional integrity of neural circuits involved in cognition. As a result, exercise contributes to the brain’s capacity to adapt to new experiences and challenges. This adaptability is particularly important in older adults, whose brains may face challenges in maintaining synaptic plasticity due to aging-related declines in BDNF levels and other factors. By enhancing synaptic plasticity, exercise helps ensure that the brain remains adaptable and capable of learning new information, even in later stages of life. This is critical for maintaining cognitive health as individuals age, as it helps mitigate the decline in cognitive function often associated with reduced plasticity in older brains [50]. In addition to enhancing synaptic function, exercise also promotes cerebral vascular health, which is vital for ensuring that the brain receives sufficient blood flow to support the metabolic demands of active neurons. Exercise increases cerebral blood flow, providing the brain with more oxygen and nutrients, and improving overall brain health. This increased blood flow also facilitates the delivery of growth factors, such as BDNF, directly to the brain, further supporting the structural changes required for neuroplasticity. Enhanced vascular health, combined with increased BDNF levels, ensures that the brain can sustain and benefit from the cognitive improvements induced by exercise. Moreover, recent studies have also suggested that exercise can counteract the effects of neurodegenerative diseases by promoting neuroprotection. Regular physical activity can help protect neurons from damage due to aging or neurodegenerative processes, such as those seen in Alzheimer’s and Parkinson’s diseases. Exercise-induced increases in oxidative stress defense mechanisms and anti-inflammatory responses help maintain neuronal function and prevent the neuronal atrophy that is characteristic of many neurodegenerative diseases. This neuroprotective effect, in combination with enhanced neurogenesis and synaptic plasticity, makes exercise an effective preventive strategy for preserving brain function in aging populations.

Furthermore, exercise has been shown to enhance the brain’s functional connectivity, or the ability of different brain regions to communicate with one another. Functional connectivity is a key feature of cognitive performance, as it supports the integration of information across different regions of the brain. Exercise improves this connectivity by stimulating the release of neurotransmitters such as dopamine and serotonin, which promote communication between neurons and help regulate mood and cognition. Regular exercise, particularly activities that engage large muscle groups, such as running, swimming, or cycling, enhance the brain’s ability to perform complex cognitive tasks that require the coordination of multiple brain regions. The effects of exercise on brain plasticity extend beyond simply enhancing learning and memory. Exercise also plays an important role in emotional regulation by modulating brain areas involved in stress and emotional processing, such as the prefrontal cortex and amygdala. Physical activity has been shown to decrease the amygdala’s reactivity to stress, improving emotional regulation and reducing symptoms of anxiety and depression. This emotional well-being effect is particularly beneficial in individuals with cognitive impairments or conditions such as MCI, where mood disturbances are often observed. Regular exercise, by promoting brain plasticity and improving emotional balance, can help individuals with cognitive decline maintain a better quality of life and a more positive outlook.

### 3.2. Neurogenesis and Brain Health

Exercise has profound effects not only on synaptic plasticity but also on neurogenesis, the process by which new neurons are generated, particularly in the hippocampus. The hippocampus is crucial for several cognitive functions, including memory formation, learning, and emotional regulation. Given the hippocampus’s essential role in cognition and mood, neurogenesis within this brain region plays a central role in maintaining healthy brain function throughout life. As individuals age, however, the rate of neurogenesis declines, contributing to cognitive impairments commonly associated with aging and neurodegenerative diseases. Fortunately, research has demonstrated that physical activity can stimulate neurogenesis and help maintain or even increase the production of new neurons, particularly in the hippocampus.

Aerobic exercise, such as running, cycling, or other forms of sustained cardiovascular activity, has been shown to promote neurogenesis in the hippocampus. Studies indicate that regular physical activity can lead to improvements in both spatial memory and working memory, both of which rely heavily on hippocampal function. For instance, exercise could significantly increase neurogenesis in the hippocampus, which was correlated with improvements in cognitive tasks that require memory retention and recall [51]. This increase in hippocampal neurogenesis is largely facilitated by the release of various growth factors, including BDNF, vascular endothelial growth factor (VEGF), and IGF-1. These growth factors are essential for supporting the survival, differentiation, and maturation of new neurons, ensuring the brain remains adaptable and capable of forming new connections. One of the primary benefits of exercise-induced neurogenesis is its ability to help prevent or slow down cognitive decline and neurodegeneration. The hippocampus is especially vulnerable to age-related changes, and its function deteriorates with advancing age. In conditions like Alzheimer’s disease and other forms of dementia, the hippocampus experiences significant atrophy, leading to profound impairments in memory and cognitive function. However, regular exercise can help mitigate this decline by preserving or even enhancing the generation of new neurons. It has been demonstrated that elderly individuals who engaged in consistent aerobic exercise showed increased hippocampal volume and improved memory function, suggesting that physical activity is an effective strategy for combating age-related cognitive decline [52].

Exercise’s impact on neurogenesis is particularly significant for older adults, especially those at risk for cognitive impairments or those already exhibiting early signs of cognitive dysfunction. In individuals with MCI, a condition often considered a precursor to Alzheimer’s disease, exercise has been shown to enhance neurogenesis in the hippocampus, improving both cognitive function and memory. Even in cases of early-stage dementia, physical activity can slow the progression of cognitive decline by stimulating the creation of new neurons and supporting brain plasticity. Additionally, exercise may help to maintain cognitive abilities for a longer period in individuals at risk of developing more severe forms of dementia. Beyond its cognitive benefits, neurogenesis also plays a crucial role in regulating mood. The hippocampus is not only involved in memory and learning but also in emotional processing. Reduced neurogenesis in the hippocampus has been linked to depression and anxiety, both of which are common in individuals experiencing cognitive decline. Exercise-induced neurogenesis has been shown to improve mood and reduce symptoms of depression and anxiety by enhancing hippocampal function. Physical activity encourages the production of neurotrophic factors that support the survival of neurons and improve their functionality, leading to better emotional regulation and mental well-being [51]. This connection between exercise, neurogenesis, and mood regulation further highlights the importance of physical activity in maintaining both cognitive function and emotional health, particularly in aging populations.

Furthermore, exercise’s influence on neurogenesis can help prevent the onset of neurodegenerative diseases. As the brain ages, its ability to regenerate new neurons diminishes, but regular physical activity can slow or even reverse this decline. The promotion of neurogenesis through exercise not only enhances cognitive abilities but also strengthens the brain’s capacity to withstand age-related neurodegeneration. In fact, studies have shown that elderly individuals who engage in regular exercise show better resistance to age-related diseases such as Alzheimer’s and Parkinson’s disease [6]. Exercise-induced increases in BDNF and other growth factors support the repair and maintenance of brain structures, helping to preserve cognitive function and delay the onset of neurodegenerative processes. Exercise also helps preserve cognitive abilities by improving vascular health, particularly through enhanced cerebral blood flow. Improved blood flow to the brain supports the delivery of oxygen and nutrients, which are necessary for neuronal function and survival. This vascular benefit, combined with the stimulation of neurogenesis, ensures that the brain has the necessary resources to maintain cognitive function. By improving the brain’s ability to deliver nutrients and remove waste products, exercise supports both the physical and functional aspects of neurogenesis.

### 3.3. Neuroinflammation and Exercise

Chronic neuroinflammation is a central factor in the progression of cognitive decline and the development of neurodegenerative diseases, including Alzheimer’s and Parkinson’s disease. Neuroinflammation arises when the brain’s immune cells, known as microglia, become activated in response to various stressors such as injury, infection, or cellular damage. In the case of neurodegenerative diseases, microglia become persistently activated, leading to the production of pro-inflammatory cytokines, such as TNF-α and IL-1β, which further damage neurons and accelerate the progression of cognitive decline. This chronic activation and inflammation plays a pivotal role in the onset and progression of these diseases, contributing to the cognitive impairments observed in aging and diseased brains.

One of the key benefits of exercise is its ability to reduce neuroinflammation and promote an anti-inflammatory environment in the brain. Regular physical activity has been shown to significantly reduce microglial activation, thereby lowering the levels of pro-inflammatory cytokines. Physical activity decreases microglial activation and the production of inflammatory markers, including TNF-α and IL-1β, which are associated with neurodegenerative processes [30]. This reduction in neuroinflammation is particularly important in mitigating the risk and progression of neurodegenerative diseases, such as Alzheimer’s, where inflammation plays a key role in neuronal damage and cognitive decline. As such, exercise has emerged as a valuable intervention in aging populations and those at risk of developing neurodegenerative conditions. In addition to its direct anti-inflammatory effects, exercise also has a significant impact on oxidative stress, another key contributor to neurodegeneration. Oxidative stress occurs when there is an imbalance between the production of ROS and the brain’s ability to neutralize these free radicals through antioxidant defenses. The accumulation of ROS causes cellular damage and inflammation, leading to neuronal injury and cognitive decline. Exercise promotes the production of antioxidants, which help counteract the effects of ROS and reduce oxidative damage in brain cells. According to Gomez-Pinilla (2008), physical activity enhances the brain’s antioxidant capacity, thereby reducing oxidative stress and protecting neurons from damage. By mitigating both oxidative stress and neuroinflammation, exercise plays a vital role in maintaining cognitive function and preventing the cellular damage that contributes to neurodegeneration [6].

The neuroprotective effects of exercise, including its anti-inflammatory and antioxidant benefits, are particularly beneficial in older adults and those at risk for age-related cognitive decline. In aging populations, neuroinflammation and oxidative stress are often elevated, leading to the breakdown of neuronal integrity and the progression of conditions like Alzheimer’s. By reducing inflammation and promoting antioxidant activity, exercise can help preserve brain health and slow the onset of cognitive decline. Regular physical activity has been associated with improved cognitive performance, reduced neuroinflammation, and enhanced neuronal resilience, particularly in older adults and individuals with MCI [50]. Moreover, the combination of reducing both neuroinflammation and oxidative stress not only benefits cognitive function but also improves overall brain health. Regular exercise helps maintain the structural integrity of the brain, preserving areas such as the hippocampus, which is crucial for learning and memory. By protecting against neuroinflammation and oxidative stress, exercise ensures that neurons remain functional and capable of adapting to new experiences, ultimately enhancing synaptic plasticity and cognitive performance. Exercise also contributes to improving vascular health in the brain, which is important for maintaining optimal brain function. The increased blood flow induced by physical activity ensures that the brain receives adequate oxygen and nutrients, supporting neuronal survival and function. This increased cerebral blood flow also helps clear waste products, including ROS, reducing the burden of oxidative damage and improving overall brain health. These combined effects on inflammation, oxidative stress, and vascular health underscore the multifaceted benefits of exercise for brain health.

### 3.4. Exercise and Cognitive Function in Aging

The impact of exercise on cognitive function is evident across the lifespan, with particularly significant benefits for aging individuals. As people age, cognitive decline becomes a more common concern, with many experiencing impairments in memory, processing speed, and executive function. These changes are often exacerbated by a reduction in physical activity, which contributes to further cognitive deterioration. However, a substantial body of research has demonstrated that regular exercise, especially aerobic exercise, can significantly slow or even reverse some of the cognitive declines associated with aging.

As individuals enter their later years, cognitive aging manifests as slower processing speed, diminished memory, and difficulties with complex cognitive tasks, including those that require executive function (planning, decision making, and attention). A decrease in the size of the hippocampus, a brain area crucial for learning and memory, is often observed in aging individuals, which contributes to declines in memory. These changes can significantly impair day-to-day function and overall quality of life. However, exercise has been shown to play a critical role in maintaining and improving cognitive health by promoting brain structures and functions essential for cognition. Regular aerobic exercise, such as running, cycling, or swimming, has been consistently linked with improved cognitive performance in older adults. It has been demonstrated that older adults who engaged in regular aerobic exercise showed significant improvements in cognitive tasks that required memory retention and processing speed [53]. Participants who exercised had superior cognitive performance compared to those who did not engage in physical activity. These improvements were not limited to basic memory tasks but extended to more complex cognitive functions, including executive control, which are vital for decision making, attention, and problem solving.

The mechanisms behind these improvements include structural changes in the brain, particularly in the hippocampus. Exercise has been found to increase hippocampal volume, a key brain region involved in memory and spatial navigation. This increase in hippocampal volume has been associated with better memory performance in older adults. Additionally, aerobic exercise enhances functional connectivity within brain networks, particularly those involved in memory and executive control, which helps improve the overall efficiency of cognitive processing. The improvements in functional connectivity suggest that exercise may help the brain adapt to the challenges of aging by supporting the communication between different brain regions required for optimal cognitive performance [53]. Moreover, exercise contributes to neuroplasticity, the brain’s ability to reorganize itself by forming new neural connections. The release of BDNF in response to physical activity supports neurogenesis and synaptic plasticity, particularly in regions such as the hippocampus. This process promotes cognitive function, supporting the ability to learn, retain new information, and adapt to new cognitive demands. Regular physical activity also helps to modulate other factors that influence brain health, including inflammation and oxidative stress, both of which can impair cognitive function as people age. In addition to improving cognitive function, exercise plays a key role in preventing neurodegenerative diseases, such as Alzheimer’s disease and dementia. A growing body of evidence suggests that individuals who engage in regular physical activity have a lower risk of developing dementia, even when adjusting to other lifestyle factors such as diet and education. Studies have shown that exercise’s effects on neurogenesis, neuroplasticity, and vascular health contribute to its ability to reduce the risk of dementia. Regular aerobic exercise has been found to delay the onset of Alzheimer’s disease by enhancing brain resilience to neurodegenerative processes [53]. For instance, the Rush Memory and Aging Project, a large-scale longitudinal study, found that higher levels of physical activity were associated with a slower rate of cognitive decline and a reduced risk of dementia in elderly individuals. This study underscored the importance of sustained physical activity for preserving cognitive function and protecting against neurodegenerative diseases. The long-term benefits of exercise on brain health are particularly important in aging populations, where early interventions can significantly delay or prevent the onset of Alzheimer’s and other forms of dementia.

Furthermore, exercise may help reduce the severity of symptoms in individuals who are already experiencing early cognitive decline. In people with MCI, a condition that often precedes Alzheimer’s disease, physical activity has been shown to improve cognitive performance, particularly in tasks related to memory and executive function. This improvement is thought to be due to the combination of exercise-induced neurogenesis, enhanced neuroplasticity, and reductions in inflammation and oxidative stress, all of which contribute to brain health and cognitive resilience. Regular physical activity also helps protect against further cognitive decline by promoting vascular health, which is essential for maintaining healthy brain function. Exercise’s protective role extends beyond cognitive function to include mental health, as physical activity has been shown to reduce symptoms of depression, anxiety, and stress, which can negatively affect cognitive performance. In fact, regular exercise is often recommended as a therapeutic intervention for improving mood and mental clarity, which in turn enhances cognitive function. This is especially relevant in aging populations, as mental health conditions can exacerbate cognitive decline and reduce quality of life. In conclusion, exercise is one of the most effective interventions for preserving cognitive function in older adults. Through its impact on brain structures like the hippocampus, the enhancement of neuroplasticity, and its ability to improve functional connectivity between brain regions, exercise significantly improves cognitive performance, particularly in memory, executive function, and processing speed. Moreover, exercise has the potential to prevent or delay the onset of Alzheimer’s disease and other forms of dementia, making it a powerful tool for maintaining cognitive health throughout aging. The combined effects of exercise on brain health, mental well-being, and the prevention of neurodegenerative diseases underscore the importance of physical activity as part of a healthy aging strategy.

### 3.5. Mental Health and Cognitive Function

Mental health conditions such as depression, anxiety, and stress can significantly impair cognitive performance, affecting memory, attention, and decision making. However, regular physical activity has been consistently shown to reduce the severity of these symptoms, thereby supporting overall cognitive health. This relationship between mental well-being and cognitive performance is largely mediated by the physiological and biochemical changes that occur in the brain during exercise.

One of the key mechanisms through which exercise improves mental health is by promoting the release of endorphins, often referred to as the brain’s “feel-good” chemicals. Endorphins are neurotransmitters that help induce feelings of relaxation and well-being, which can counteract the negative effects of stress, anxiety, and depression. By stimulating the release of endorphins, exercise can help regulate mood and reduce the cognitive impairments often associated with mental health disorders. This effect is particularly beneficial for individuals suffering from conditions like major depressive disorder and generalized anxiety disorder (GAD), as these conditions are frequently linked to cognitive dysfunction [54]. In addition to boosting mood, endorphins can also promote feelings of calmness and emotional balance, which further supports cognitive performance by improving focus and mental clarity. Exercise has also been shown to enhance cognitive resilience by improving mental clarity and boosting energy levels. Many mental health disorders are accompanied by cognitive fatigue, which can hinder concentration, memory, and decision-making abilities. Regular physical activity helps combat this fatigue by increasing blood flow to the brain, providing neurons with more oxygen and nutrients, and promoting the generation of neurotrophic factors like BDNF. These factors not only improve cognitive function but also help protect the brain from the negative effects of stress. Increased blood flow and enhanced neuronal function contribute to better cognitive performance, particularly in tasks that require attention and memory [50]. The benefits of exercise on mental health and cognitive function are particularly pronounced in individuals with chronic conditions such as MCI or early-stage Alzheimer’s disease. In these populations, mood disturbances are common and can exacerbate cognitive decline. Regular exercise has been shown to reduce the symptoms of depression and anxiety in individuals with cognitive impairments, contributing to improved cognitive outcomes and an overall enhanced quality of life. For example, exercise interventions significantly reduced depression and anxiety in individuals with MCI, which in turn led to improvements in cognitive performance, particularly in tasks involving memory and executive function. These improvements in cognitive function are attributed to exercise’s ability to increase neuroplasticity and neurogenesis, as well as its effects on reducing inflammation and oxidative stress, both of which are elevated in individuals with MCI and early-stage dementia [54].

Exercise also plays a role in enhancing stress resilience, which is essential for maintaining cognitive function in the face of mental and physical stressors. Chronic stress can have detrimental effects on the brain, particularly in areas involved in memory and learning, such as the hippocampus and prefrontal cortex. Chronic activation of the hypothalamic–pituitary–adrenal (HPA) axis, a system that regulates the body’s stress response, leads to increased levels of cortisol, a hormone that can damage neurons and impair cognitive performance over time. Exercise has been shown to regulate the HPA axis, reducing cortisol levels and promoting a more balanced stress response. This is particularly important for older adults and individuals with cognitive decline, as chronic stress and high cortisol levels are strongly linked to the acceleration of cognitive decline and neurodegenerative diseases like Alzheimer’s [52]. Beyond reducing depression and anxiety, exercise can also improve sleep quality, which is essential for maintaining cognitive health. Many individuals with mental health conditions, particularly depression and anxiety, experience disrupted sleep patterns, which in turn affect cognitive performance during the day. Regular physical activity has been shown to improve sleep quality and help regulate sleep–wake cycles, contributing to better cognitive function and mood [55]. The ability to sleep more soundly not only aids in emotional recovery but also supports cognitive processes such as memory consolidation and learning.

## 4. Mechanisms of Neuroplasticity: Nutrition and Exercise

Neuroplasticity, the nervous system’s capacity to adapt structurally and functionally in response to intrinsic and extrinsic stimuli, has emerged as a central theme in contemporary neuroscience. Over the last few decades, researchers have increasingly focused on identifying factors that enhance neural plasticity, given its potential to counter cognitive decline, improve quality of life in older adults, and mitigate the impact of various neurological disorders. Among the most promising modulators are nutrition and physical exercise, two lifestyle components over which individuals can exert substantial control. Accumulating evidence demonstrates that dietary patterns, especially those rich in polyunsaturated fatty acids, polyphenols, fibers, and essential micronutrients, combined with regular physical exercise (notably moderate-to-vigorous aerobic activities) can exert synergistic effects on brain function. These effects unfold across multiple biological strata. At the molecular and cellular levels, improved nutritional quality and systematic exercise enhance neurotrophic factor expression, optimize energy metabolism, and facilitate epigenetic modifications that promote synaptic plasticity. At higher organizational levels, these interventions can foster adult hippocampal neurogenesis, modulate neurotransmitter balance, and refine neural circuit connectivity. Moreover, the interplay between diet and exercise can attenuate inflammation, oxidative stress, and hormonal imbalances—environmental conditions that otherwise hamper neuroplastic potential. By reducing these detrimental factors, a virtuous cycle emerges wherein neural networks become more resilient and better equipped to respond adaptively to various cognitive challenges (Table 1).

The synergistic effects of nutrition and exercise on neuroplasticity and cognitive performance have been extensively documented in the scientific literature. Dominguez et al. (2021) emphasize that omega-3 fatty acids, polyphenols, and other bioactive compounds play a crucial role in regulating neurotrophic factor expression, reducing oxidative stress, and modulating neuroinflammatory pathways, all of which contribute to cognitive resilience and mental health optimization [84]. Similarly, Ngandu et al. (2015) demonstrated that a multidomain intervention combining dietary modifications with structured physical and cognitive training significantly enhanced cognitive function and reduced the risk of dementia in an at-risk elderly population, reinforcing the importance of integrated lifestyle interventions [85]. Additionally, a recent study in the *International Journal of Behavioral Nutrition and Physical Activity* (2024) examined the effects of single and combined dietary and exercise interventions on cognitive outcomes, concluding that the interaction between these factors yields greater benefits than either intervention alone [86]. 

## 5. Myokines and Neurotrophins: Exercise-Induced Brain Factors

Physical exercise is a powerful, multifaceted stimulus known not only to strengthen muscles and improve cardiovascular health but also to profoundly influence brain function. Over the past two decades, scientific inquiry has shifted from simply observing the cognitive benefits of exercise to understanding the intricate cellular and molecular mechanisms that underlie these effects. Among the most compelling discoveries is the role of myokines—muscle-derived signaling factors—and neurotrophins—brain-derived growth factors—in mediating the adaptive responses of the central nervous system (CNS) to regular physical activity.

**Figure 1:**
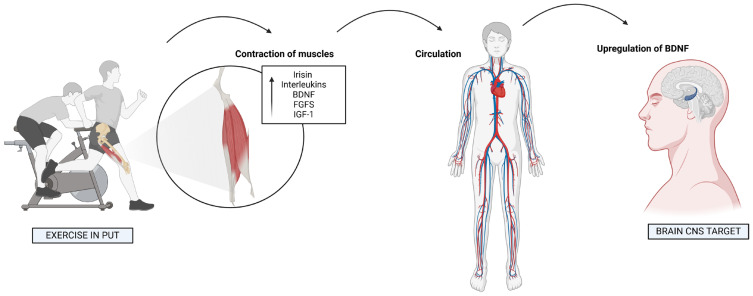
Impact of physical exercise on brain function via myokines and neurotrophins.

Traditionally, skeletal muscle was conceptualized primarily in terms of its contractile properties and mechanical outputs, such as force production and locomotion. However, a paradigm shift has positioned muscle as a dynamic endocrine organ capable of producing and secreting a diverse array of signaling molecules, termed myokines [83,84]. These myokines are released into the bloodstream during and after both acute and chronic exercise stimuli, influencing not only peripheral metabolic pathways and inflammatory processes but also exerting significant effects on the CNS. Physical exercise enhances brain function by stimulating the release of key molecules such as Irisin, IL-6, BDNF, FGFs, and IGF-1. Irisin, produced by muscles, promotes BDNF expression, which supports neuroplasticity and cognitive function. IL-6 helps regulate inflammation and metabolism, while FGFs and IGF-1 contribute to neuroprotection and synaptic remodeling. These factors collectively highlight the crucial role of exercise in improving brain health and preventing cognitive decline (Figure 1).

Such endocrine communication is increasingly viewed as a key mediator of the cognitive and neuroprotective benefits of physical activity. Among the best-characterized myokines is irisin, a peptide hormone cleaved from the membrane protein FNDC5. Irisin was initially identified for its role in stimulating the browning of white adipose tissue, thereby improving systemic metabolic homeostasis and energy expenditure [57,85]. In recent years, attention has shifted to its effects on the brain. Notably, irisin can cross the blood–brain barrier, and growing evidence indicates that it upregulates the expression of BDNF within the hippocampus—a brain region critical for learning, memory consolidation, and spatial navigation. Experimental studies in rodents demonstrate that elevated irisin levels correlate with increased synaptic plasticity, enhanced long-term potentiation, and improved performance in memory tasks [58,86]. Although the precise receptor mechanisms for irisin in the CNS remain elusive, these findings underscore the importance of muscle-derived factors in sculpting the neural landscape.

Cathepsin B represents another exercise-responsive myokine that has attracted attention due to its association with cognitive health. Research indicates that physically active rodents and humans exhibit higher circulating cathepsin B levels, which correlate with enhanced hippocampal function and improved outcomes in memory tests [85,87]. Although the underlying molecular pathways are not fully elucidated, it is hypothesized that cathepsin B may modulate gene expression profiles influencing synaptic remodeling, adult neurogenesis, and neuronal survival. Such gene expression changes could involve the regulation of synaptic proteins, growth factors, or signaling cascades essential for maintaining and strengthening neuronal circuitry. Interleukin-6 (IL-6), a well-known cytokine produced transiently by contracting muscle fibers during exercise, also contributes to muscle–brain communication. While chronic elevations in IL-6 and other pro-inflammatory markers are deleterious to brain health, the acute, exercise-induced surge in IL-6 may serve a beneficial, regulatory function [83,88]. In this context, IL-6 can act as a metabolic sentinel, informing the brain of the body’s current energetic state and availability of substrates. By adjusting neural energy utilization and potentially modulating microglial activity, short-term IL-6 increases could create an environment more conducive to neuroplasticity and adaptive remodeling. This finely tuned inflammatory signal may help “prime” the brain to respond more effectively to neurotrophic factors, synaptogenic cues, and other beneficial molecular changes induced by regular physical activity. Collectively, these findings highlight a complex, bidirectional dialogue between skeletal muscle and the brain. Through the release of myokines such as irisin, cathepsin B, and IL-6, exercise exerts a profound influence on the CNS, shaping synaptic networks, enhancing cognitive reserve, and ultimately contributing to improved cognitive function and resilience against neurodegenerative insults. As research advances, further identification of specific myokine receptors, downstream signaling targets, and temporal dynamics will refine our understanding of this muscle–brain axis, opening avenues for targeted interventions to optimize brain health through exercise-based strategies.

Furthermore, neurotrophins are secreted proteins essential for the survival, differentiation, and growth of neurons. Among these molecules, BDNF has emerged as a principal mediator of exercise-induced enhancements in brain function. BDNF supports synapse formation and stability, modulates neurotransmitter release, and is integral to long-term potentiation a cellular mechanism underlying learning, memory formation, and the capacity to adapt to new information [80,89]. A range of physical activities has been shown to elevate BDNF levels within key brain regions. Regular endurance-based routines, such as running or cycling, consistently boost BDNF concentrations in areas involved in higher cognitive processes, including the hippocampus and prefrontal cortex [56,58]. The resulting increase in BDNF correlates strongly with improvements in neurogenesis, synaptic efficiency, and overall cognitive performance. While sustained aerobic exercise has received the most attention, emerging evidence indicates that other exercise modalities—such as resistance training and high-intensity interval training—can also elevate BDNF levels, though the timing, magnitude, and persistence of these changes may depend on variables like intensity, duration, and individual fitness status [90]. Beyond BDNF, additional neurotrophins, including Nerve Growth Factor (NGF) and Neurotrophin-3 (NT-3), are influenced by regular physical activity. Although these factors are less extensively studied in the context of exercise, they likely contribute synergistically to creating a pro-plasticity environment, promoting more robust dendritic arborization, supporting synaptic resilience, and enhancing neuronal survival [86,91]. In this way, exercise-induced elevations in multiple neurotrophins, rather than a single factor, collectively shape a neural landscape primed for learning, memory consolidation, and long-term cognitive health.

The interaction between myokines and neurotrophins is both reciprocal and synergistic, shaping a dynamic signaling network that transmits exercise-induced peripheral cues to central neural circuits. One of the most well-characterized pathways involves the transcriptional coactivator Peroxisome Proliferator-Activated Receptor Gamma Coactivator-1 Alpha (PGC-1α), which is activated in skeletal muscle during physical activity. PGC-1α enhances the transcription of Fibronectin Type III Domain-Containing Protein 5 (FNDC5), leading to increased release of irisin into the bloodstream [57]. Upon reaching the CNS, irisin modulates gene expression, ultimately stimulating the production and release of BDNF. This establishes a positive feedback loop, wherein heightened physical activity augments BDNF availability, reinforcing neuroplastic adaptations and cognitive benefits [84]. Although the precise irisin receptor within the CNS remains elusive, growing evidence indicates that irisin engages intracellular signaling cascades involving cyclic adenosine monophosphate (cAMP) Response Element-Binding Protein (CREB), a transcription factor critical for BDNF gene expression and synaptic plasticity. Similarly, cathepsin B, another exercise-responsive myokine, may refine gene networks that underlie synaptic remodeling and neuronal resilience, potentially collaborating with neurotrophic factors and additional growth signals to maintain a robust and adaptable neural architecture [85]. IL-6, secreted acutely by muscle fibers during exercise, also contributes to this crosstalk through subtler, more indirect mechanisms. By modulating inflammatory and metabolic pathways, IL-6 may optimize the microenvironment for neurotrophin actions. A finely tuned inflammatory response can clear damaged synapses and bolster those that are functionally active, ensuring that BDNF and related neurotrophins operate under conditions conducive to sustained synaptic health and plasticity [83,88].

At the intracellular level, exercise-induced adaptations are orchestrated by a complex interplay of metabolic and signaling pathways. A prominent example is the 5’ Adenosine Monophosphate-Activated Protein Kinase (AMPK)–PGC-1α axis, which responds to shifts in muscle energy status triggered by repetitive contractions. Activation of PGC-1α in muscle cells not only promotes mitochondrial biogenesis and enhances metabolic efficiency but also influences the expression of FNDC5, thereby controlling irisin release [60,89]. Once irisin enters the CNS, it likely interacts with putative receptors on neurons or glial cells, initiating a cascade of intracellular events culminating in CREB activation. The resulting transcriptional upregulation of BDNF supports synaptic differentiation, spine formation, and synaptic efficacy. When BDNF binds to its cognate high-affinity receptor, Tropomyosin Receptor Kinase B (TrkB), it triggers downstream pathways such as Mitogen-Activated Protein Kinase/Extracellular Signal-Regulated Kinase (MAPK/ERK) and Phosphoinositide 3-Kinase (PI3K)/Akt. These cascades converge on translational and cytoskeletal regulators that increase synaptic protein synthesis, augment dendritic spine density, and strengthen excitatory synaptic connections [80,89]. Beyond molecular signaling events, exercise-induced improvements in cerebrovascular perfusion and angiogenesis, partly mediated by VEGF, reinforce these adaptive outcomes. Enhanced delivery of oxygen and nutrients to active neurons supports neurogenesis, synaptic remodeling, and efficient neurotransmission. The integration of metabolic optimization, myokine–neurotrophin signaling, and improved vascular support is instrumental in consolidating the neural benefits of exercise [75,92]. Ultimately, this multifactorial framework explains how repetitive bouts of physical activity reshape the brain’s structural and functional landscape, fostering cognitive resilience and long-term neurological health.

The impact of exercise on myokine and neurotrophin signaling—and, by extension, on brain function—is not uniform. Instead, it varies considerably depending on the modality, intensity, duration, and frequency of physical activity. Understanding these nuances is essential for tailoring exercise prescriptions to optimize cognitive and neurological benefits. Sustained moderate to vigorous intensity aerobic exercise (running, cycling, swimming) is among the most studied modalities in the context of neuroplasticity. Endurance training enhances cardiovascular fitness, increases cerebral blood flow, and promotes favorable metabolic adaptations. These systemic improvements synergize with elevated myokine (irisin) and neurotrophin (BDNF) levels, ultimately augmenting hippocampal neurogenesis, synaptic remodeling, and cognitive performance [56,59]. Over time, these adaptations may translate into improved memory retention, enhanced executive functions, and greater resilience against age-related cognitive decline. HIIT involves short bursts of high-intensity activity alternated with brief recovery periods. This protocol can induce rapid increases in metabolic stress and trigger distinctive molecular cascades. While studies indicate that HIIT can acutely elevate BDNF levels, sometimes more robustly than continuous moderate-intensity exercise, the sustainability and longitudinal impact of these spikes on long-term cognitive outcomes remains an area of active investigation [90]. Nevertheless, HIIT’s time-efficient model and potential to induce acute cognitive gains (improved attentional control, faster information processing) make it an attractive option for individuals to seek quicker, though sometimes transient, neural benefits. Strength exercise (weightlifting, bodyweight exercises, resistance bands) primarily target skeletal muscle hypertrophy, increased muscle strength, and improved metabolic health. Although resistance training may not consistently produce large, immediate elevations in BDNF relative to aerobic modalities, it favorably influences systemic parameters—such as glucose metabolism, inflammatory status, and hormone profiles—that indirectly support neuronal health and plasticity [75,93]. Over the longer term, enhancing muscle mass and metabolic resilience may establish an internal environment that supports stable myokine and neurotrophin signaling, potentially offering protective effects against neurodegeneration and contributing to improved cognitive function, particularly in aging individuals.

Exercises that integrate low to moderate physical effort with mindfulness, breath control, and proprioceptive awareness, such as yoga, tai chi, and qigong have also attracted increasing research interest. While traditionally less studied from a strictly molecular perspective, emerging evidence suggests that these practices may modulate stress-responsive hormonal and inflammatory pathways, thereby influencing brain plasticity indirectly [94]. Over time, the subtle improvements in autonomic regulation and mood may support healthier patterns of myokine and neurotrophin release, enhancing cognitive–emotional balance and potentially reinforcing resilience to neurocognitive stressors. A growing body of literature advocates for combining different exercise modalities to achieve synergistic or additive effects on cognitive health. For instance, integrating aerobic and resistance training can harness the strengths of both modalities: the robust neurotrophin elevations commonly seen with aerobic exercise and the favorable metabolic and hormonal environment cultivated by resistance training [95]. Similarly, pairing aerobic exercise with mind–body interventions may amplify stress-buffering effects, thereby further optimizing conditions for plasticity-related signaling molecules to operate (Table 2).

## 6. Gut–Brain Axis: Nutrition and Cognitive Function

Beyond considering the brain in isolation, the gut–brain axis has emerged as a pivotal area of research—a bidirectional communication network linking the central nervous system (CNS) and the gastrointestinal tract. This complex interplay involves neural (via the vagus nerve), hormonal (e.g., gut-derived peptides like ghrelin and leptin), and immunological signaling pathways, as well as microbial metabolites produced by the gut microbiota. Nutrition is a primary modulator of this axis; dietary choices directly shape the composition and activity of the gut microbiome, influencing the production of metabolites such as short-chain fatty acids (SCFAs), tryptophan metabolites, and bile acids, which interact with the CNS to regulate brain function. These metabolites can modulate neuroinflammation, neuroplasticity, and neurotransmitter synthesis, directly impacting cognitive outcomes and mood regulation.

The gut microbiome—a complex ecosystem composed of trillions of microorganisms, including bacteria, archaea, viruses, and fungi—plays a critical role in bridging the effects of diet and exercise on brain health. For instance, the fermentation of dietary fibers by gut bacteria produces SCFAs (e.g., acetate, propionate, and butyrate), which serve as energy sources for colonocytes, maintain gut barrier integrity, and cross the blood–brain barrier to influence neuronal health and neurogenesis. Additionally, microbial-derived tryptophan metabolites are precursors to serotonin, a neurotransmitter critical for mood and cognitive function. Regular physical activity complements these effects by promoting gut microbial diversity, increasing SCFA production, and reducing systemic inflammation, further strengthening the gut–brain connection. Understanding these interconnections provides opportunities for tailored dietary and exercise interventions aimed at optimizing cognitive function, preventing neurodegenerative diseases, and managing mood disorders [96]. This microbial community is highly sensitive to dietary composition. For instance, a diet rich in fermentable fibers (fruits, vegetables, whole grains, legumes) supports a more diverse and stable microbiota, conducive to producing beneficial metabolites and maintaining intestinal barrier integrity. Conversely, diets high in saturated fats and simple sugars may reduce bacterial diversity and favor the expansion of proinflammatory species. Such dietary patterns have been associated with poorer metabolic profiles and, increasingly, with cognitive deficits, reduced synaptic plasticity, and heightened neuroinflammation [97]. As the understanding of the gut microbiome advances, researchers have begun to map specific bacterial taxa and metabolic signatures to cognitive outcomes. Identifying these microbial “fingerprints” is an ongoing challenge, as factors like genetic predisposition, early life microbial exposures, antibiotics, and overall health all influence the gut ecosystem. Nevertheless, the growing body of evidence highlights the central role that gut microbes play in shaping the neural substrate for learning, memory, and executive functions.

Several mechanisms facilitate communication between the gut and the CNS, creating a sophisticated, bidirectional signaling network. Neural pathways such as the vagus nerve, which innervates the gastrointestinal tract, transmits signals from the gut to the brain. Changes in gut microbial composition, intestinal permeability, or the presence of specific microbial metabolites can alter vagal signaling. These changes, in turn, may influence stress responses, mood regulation, motivational states, and even learning and memory processes [98]. The gut essentially “talks” to the brain, providing continuous updates about nutrient status, immune challenges, and microbiome balance. In addition, the gut is a key immunological hub, housing the largest concentration of immune cells in the body. Intestinal barrier integrity and the balance of gut microbes influence systemic inflammation. Dysbiosis (an imbalance in gut microbial composition) can weaken the intestinal barrier, allowing microbial antigens or proinflammatory mediators to enter circulation. Some of these factors may cross the blood–brain barrier (BBB), triggering neuroinflammation and microglial activation that can impair synaptic plasticity and contribute to cognitive decline [99]. Along these lines, gut microbes produce a range of bioactive compounds, including SCFAs (such as butyrate, acetate, and propionate), secondary bile acids, and neurotransmitter molecules (GABA, serotonin precursors, dopamine precursors). SCFAs have been shown to influence BBB integrity, modulate microglial activity, and promote synaptic plasticity. Butyrate, for example, can regulate gene expression in the brain through epigenetic mechanisms, enhancing the availability of neurotrophic factors and potentially improving cognitive function [100]. Moreover, the HPA axis, a major stress response system, is influenced by gut microbes. Chronic dysbiosis or gut inflammation can exacerbate HPA axis dysregulation, elevating cortisol levels and impairing cognitive processes. Conversely, a balanced gut ecosystem can help normalize stress hormone regulation, promoting resilience to cognitive challenges and reducing vulnerability to anxiety and depression-like behaviors [101].

Nutrition affects the gut microbiome and its downstream effects on brain health through multiple dietary components and patterns. Prebiotics, which are nondigestible fibers selectively fermented by beneficial gut microbes, shift the microbiome towards a composition enriched in commensal bacteria like Bifidobacterium and Lactobacillus. These bacteria produce SCFAs and anti-inflammatory compounds that support gut barrier function and may enhance cognitive outcomes. Studies in both humans and animal models have demonstrated that diets rich in prebiotic fibers can improve learning, memory, and reduce anxiety behaviors [102]. In addition, probiotics, live microorganisms that confer health benefits when administered in adequate amounts, can alter neurotransmission, improve gut barrier integrity, and reduce systemic inflammation. Certain probiotic strains (Lactobacillus rhamnosus JB,1) influence GABA receptor expression in the brain and mitigate stress-induced behaviors in animal models [103]. While clinical results vary, these findings point towards probiotics as potential tools to modulate cognitive function and emotional well-being.

Polyphenols and Phytochemicals: Plant-derived compounds like polyphenols (found in berries, cocoa, tea, and red wine) have antioxidant and anti-inflammatory properties that extend from the gut to the brain. Many polyphenols are metabolized by gut microbes into bioactive metabolites that can cross the BBB and enhance synaptic signaling. Epidemiological and intervention studies suggest that polyphenol-rich diets improve cognitive performance and may reduce the risk of neurodegenerative diseases by enhancing neuronal resilience and dampening inflammation [104]. Long-chain omega-3 fatty acids such as DHA and EPA influence gut microbial composition and exert neuroprotective effects. They support synaptogenesis, maintain neuronal membrane fluidity, and modulate neuroinflammation. While research into the gut microbiota-mediated effects of omega-3s is ongoing, there is suggestive evidence that combining DHA/EPA with prebiotics or other beneficial dietary components could synergistically improve cognitive function [105]. In addition, fermented products like yogurt, kefir, kimchi, and sauerkraut contain beneficial microorganisms and microbial metabolites. Regular consumption of these foods can reinforce gut microbiota diversity, modulate neurotransmitter production, and potentially reduce anxiety and depression symptoms, changes that may indirectly support cognitive function and protect against age-related cognitive decline [106].

The gut–brain axis plays a pivotal role not only in adults but also during critical windows of development. In early life, the microbiome is dynamically shaped by factors such as mode of birth delivery (vaginal vs. cesarean), breastfeeding vs. formula feeding, and early dietary exposures. The first two to three years of life are critical for establishing a stable gut microbial community, which can influence the trajectory of brain maturation, neural circuit formation, and cognitive development [107]. Emerging evidence suggests that disruptions to the early microbiome—due to antibiotic exposure, poor maternal diet, or lack of breastfeeding—can alter synaptic pruning, neurotransmitter systems, and stress reactivity in ways that may predispose individuals to cognitive deficits and mood disorders later in life. Interventions aimed at optimizing the infant gut microbiota through maternal nutrition, probiotics, and supportive early dietary patterns may have lasting positive impacts on cognitive outcomes across the lifespan, setting a foundation for better mental health and academic performance.

Dysbiosis, characterized by reduced microbial diversity and an overabundance of pathogenic or proinflammatory species, has been implicated in various neurological and psychiatric conditions, including Alzheimer’s disease, Parkinson’s disease, autism spectrum disorders, and depression [108]. While disentangling cause from effect is challenging, growing evidence suggests that modifying the gut microbiome through targeted dietary interventions like adding prebiotics, probiotics, or increasing fiber intake may slow disease progression or improve cognitive symptoms. For example, individuals with Alzheimer’s disease often exhibit altered gut microbiota profiles and increased intestinal permeability. Preclinical studies have demonstrated that dietary enrichment with prebiotics or certain probiotics can reduce amyloid-beta accumulation and enhance cognitive performance in mouse models. Similarly, in Parkinson’s disease, gut microbial imbalances may contribute to alpha-synuclein misfolding and neurodegeneration. Emerging data indicate that dietary strategies aiming to restore healthy gut microbiota could slow disease onset or symptom severity [109].

Although much of the foundational research on the gut–brain axis has been conducted in animal models, clinical studies are increasingly validating these findings in humans. Several randomized controlled trials have investigated the cognitive effects of probiotic supplementation. While results are mixed—likely due to differences in probiotic strains, dosing strategies, participant demographics, and outcome measures—some studies report improvements in memory, executive function, and stress resilience in participants consuming specific probiotic blends [110]. Dietary interventions, too, have shown promise. Adhering to the Mediterranean diet, rich in fruits, vegetables, whole grains, legumes, fish, and olive oil, correlates with greater gut microbial diversity and better cognitive performance in older adults. Longitudinal and interventional studies suggest that improving diet quality over time aligns with slowed cognitive decline, reduced inflammation, and lower risk of dementia. These findings highlight the potential for diet-based strategies to preserve cognitive health by cultivating a beneficial gut microbiota environment [111]. The responsiveness of the gut microbiome to dietary changes is highly individualized. Genetic background, early life exposures, and current health status shape how a person’s microbiota responds to new eating patterns. Consequently, not everyone will experience the same cognitive benefits from identical nutritional interventions. As a result, researchers are exploring the field of precision nutrition to develop personalized dietary recommendations based on an individual’s microbiome profile, biomarkers of inflammation, and metabolic parameters. Such a tailored approach could identify who stands to benefit most from dietary fibers, polyphenol-rich foods, or probiotic supplementation to enhance cognitive outcomes and mental well-being.

The term “psychobiotics” refers to probiotics, prebiotics, or fermented foods that positively influence mental health through the gut–brain axis [112]. Psychobiotics can shape neurotransmitter systems, alter stress responses, and potentially improve mood and cognition. Early-stage research on psychobiotics suggests that certain bacterial strains and dietary fibers can reduce anxiety, depressive symptoms, and cognitive impairments in preclinical models and some human trials. While the field is young and further rigorous investigation is needed, psychobiotics represent a promising avenue for dietary-based mental health and cognitive interventions. A deeper understanding of how microbial metabolites influence the brain is crucial for developing effective dietary strategies. SCFAs like butyrate can regulate gene expression in neurons, support BBB integrity, and influence synaptic plasticity. Enhancing butyrate production via increased fiber intake or targeted prebiotic supplementation might promote neurotrophic signaling, reduce neuroinflammation, and bolster memory processes [112]. Other microbial-derived compounds, such as tryptophan metabolites, GABA, and catecholamines, may influence neurotransmitter balance, modulating mood, motivation, and learning capacity. Adjusting dietary components to increase beneficial tryptophan metabolism, for instance, could enhance serotoninergic signaling and improve emotional regulation alongside cognitive performance.

While no single diet guarantees improved cognition for everyone, certain patterns appear consistently supportive. Diets abundant in whole, minimally processed foods—rich in dietary fibers, polyphenols, omega-3 fatty acids, and fermented products—generally promote microbial diversity and beneficial metabolite production. The Mediterranean diet, for example, has been associated with improved metabolic health, reduced systemic inflammation, and better cognitive trajectories [113]. By contrast, Western-style dietary patterns high in refined carbohydrates, saturated fats, and processed foods are linked to dysbiosis, metabolic dysfunction, and an increased risk of cognitive decline. Studies in animal models show that these diets can induce memory deficits and lower hippocampal BDNF levels, in part through changes in the gut microbiota [114]. Thus, shifting dietary habits toward more plant-based, fiber-rich patterns may help maintain cognitive vitality across the lifespan. Thus, despite rapid progress, numerous questions remain. Disentangling causality from correlation in the microbiome–brain relationship demands more longitudinal, carefully controlled human studies. Identifying biomarkers and microbiome signatures predictive of individual responsiveness to dietary interventions will be crucial for personalizing treatments. Mechanistically, we still need a clearer map of how SCFAs, neurotransmitter-like metabolites, and inflammatory mediators operate at the molecular level to alter neural circuits and cognitive processes.

Translating findings into clinical practice also poses challenges. Long-term adherence to dietary interventions, variability in microbiome composition among populations, and complex interactions with other lifestyle factors necessitate a multifaceted approach. Finally, examining how early-life interventions might sculpt a microbiome that confers durable cognitive resilience, or how adjustments during aging might prevent cognitive decline, are avenues ripe for exploration. The gut–brain axis represents a paradigm shift in understanding cognitive function, integrating the gut microbiota and nutrition into the broader context of neural health. Far from being isolated, the brain is profoundly influenced by dietary choices that shaped gut microbial communities, metabolic signaling pathways, and the inflammatory milieu. By strategically leveraging nutrition—via prebiotics, probiotics, polyphenol-rich foods, fermented products, and balanced dietary patterns—we may enhance cognitive resilience, reduce the risk of neurodegenerative disorders, and improve mental health outcomes.

## 7. Oxidative Stress and Neuroprotection: Antioxidants in Action

The human brain, although representing only about 2% of the total body mass, consumes roughly 20% of the body’s resting oxygen supply, and this disproportionate metabolic demand makes it uniquely vulnerable to oxidative stress [115,116]. Oxidative stress arises when the production of ROS and reactive nitrogen species (RNS) exceeds the capacity of intrinsic antioxidants and repair systems, leading to the accumulation of damaged proteins, lipids, and nucleic acids within neuronal circuits [115,117]. Under normal conditions, ROS and RNS serve as signaling molecules and are effectively neutralized by endogenous enzymatic antioxidants such as superoxide dismutase (SOD), glutathione peroxidase (GPx), and catalase, as well as nonenzymatic antioxidants like glutathione and vitamins C and E [118]. However, with advancing age, environmental stressors, poor diet, and certain genetic backgrounds, the delicate redox balance may shift, allowing oxidative damage to accumulate [116,119].

The central nervous system’s heightened susceptibility to oxidative damage is multifactorial. First, neurons rely heavily on aerobic metabolism for ATP production, rendering them prone to mitochondrial ROS leakage, especially under conditions of metabolic stress [120,121]. Second, neuronal membranes are rich in polyunsaturated fatty acids (PUFAs), which are highly susceptible to peroxidation, compromising membrane fluidity and receptor function [115,117]. Third, the adult brain has limited regenerative capacity, making oxidative damage more consequential over time [122]. These vulnerabilities help explain why oxidative stress is implicated in numerous neurodegenerative diseases, including Alzheimer’s disease, Parkinson’s disease, and amyotrophic lateral sclerosis, all of which share a common thread of elevated ROS production, diminished antioxidant defenses, and progressive neuronal loss [123,124]. In Alzheimer’s disease, oxidative modifications to proteins, lipids, and nucleic acids occur early in pathogenesis, potentially accelerating amyloid-beta (Aβ) plaque formation and tau hyperphosphorylation [124]. In Parkinson’s disease, the loss of dopaminergic neurons in the substantia nigra pars compacta is intimately linked to mitochondrial dysfunction, iron accumulation, and unchecked oxidative damage [125]. Similar patterns, including impaired energy metabolism and glial-mediated inflammatory responses, are observed in other conditions like Huntington’s disease and vascular cognitive impairment [116,120]. Together, these findings underscore the importance of interventions that maintain or restore redox homeostasis, as controlling oxidative stress represents a promising avenue for neuroprotection and preservation of cognitive function.

Mounting evidence indicates that nutrition and lifestyle factors critically shape the brain’s antioxidant capacity and susceptibility to oxidative injury [119]. A balanced, nutrient-dense diet provides essential micronutrients—such as vitamins E and C—that directly scavenge free radicals, as well as trace elements like selenium, copper, zinc, and manganese that serve as cofactors for key antioxidant enzymes [118]. For instance, vitamin E, a lipid-soluble antioxidant, protects cell membranes against lipid peroxidation, while vitamin C, a water-soluble antioxidant, can regenerate vitamin E from its oxidized form, creating a synergistic antioxidant network [126]. Beyond vitamins and minerals, bioactive phytochemicals found in fruits, vegetables, tea, wine, and spices are increasingly recognized for their neuroprotective properties. Polyphenols such as flavonoids (quercetin, anthocyanins), catechins (epigallocatechin gallate from green tea), resveratrol (from grapes and red wine), and curcumin (from turmeric) can not only scavenge ROS but also modulate cellular signaling pathways that regulate antioxidant gene expression, inflammation, and synaptic plasticity [122,127]. These compounds may exert their benefits partly through the activation of the transcription factor Nrf2, which controls the expression of numerous antioxidant and detoxification genes, thereby enhancing endogenous defenses [118].

Whole food-based dietary patterns, rather than single nutrients, appear most effective for long-term cognitive protection [128]. Diets rich in colorful fruits, leafy greens, nuts, seeds, whole grains, and fish—key elements of the Mediterranean or DASH diets—are consistently associated with improved cognitive function, reduced risk of dementia, and lower oxidative stress markers [122,127]. Such patterns also often contain omega-3 fatty acids like DHA, which can modulate oxidative stress and inflammation indirectly by optimizing neuronal membrane composition and supporting mitochondrial efficiency [120]. In contrast, Western-style diets high in refined sugars, saturated fats, and processed foods tend to promote oxidative stress, insulin resistance, and low-grade inflammation, thereby exacerbating cognitive decline over time [129]. Lifestyle factors complement dietary strategies for mitigating oxidative stress. Regular physical exercise enhances antioxidant enzyme activity, improves mitochondrial biogenesis, and increases cerebral blood flow, collectively reducing oxidative burden in the brain [130]. Moderate-intensity aerobic exercise, for example, can upregulate SOD and GPx, enhancing the overall antioxidant network [130]. Intermittent fasting or caloric restriction regimens also show promise, as mild metabolic stress can induce hormetic responses, triggering endogenous antioxidant defenses and strengthening neuronal resistance to ROS-mediated injury [126].

Stress management and adequate sleep further support redox homeostasis. Chronic psychological stress elevates cortisol and catecholamine levels, which can increase ROS production and impair antioxidant defenses, while poor sleep quality disrupts metabolic and immune homeostasis, exacerbating oxidative damage [116]. Mindfulness practices, yoga, and proper sleep hygiene may indirectly improve redox balance by stabilizing hormonal fluctuations, reducing inflammation, and supporting the clearance of metabolic waste from the brain [120]. While antioxidant supplements have gained public interest, their efficacy in neuroprotection remains equivocal. Some large-scale clinical trials testing high-dose vitamin E or vitamin C have yielded modest or inconclusive results, and concerns arise that excessive supplementation might interfere with normal redox signaling or fail to reach critical targets within the CNS [115]. Similarly, polyphenol extracts (curcumin or resveratrol pills) have shown variable outcomes in humans, possibly due to poor bioavailability, suboptimal dosing, or interactions with individual gut microbiomes and metabolic profiles [127]. These considerations underscore the importance of focusing on whole food sources, dietary diversity, and lifestyle modifications rather than relying on isolated antioxidant supplements.

As research on oxidative stress and neuroprotection advances, emerging technologies and conceptual frameworks offer new avenues for both prevention and therapy. The quest to identify reliable biomarkers of oxidative damage (lipid peroxidation products, oxidized DNA bases, or advanced oxidation protein products) and antioxidant status can guide personalized interventions. For example, metabolomics, lipidomics, and proteomics approaches enable detailed mapping of redox perturbations within specific brain regions and patient populations [117]. Coupled with genetic and epigenetic profiling, these data may help clinicians identify individuals at higher risk for oxidative damage-related cognitive decline, enabling earlier and more tailored nutritional and lifestyle recommendations. The integration of precision nutrition concepts with personalized medicine is another frontier. Just as certain genotypes influence how people metabolize nutrients, variations in gut microbiota composition could dictate how effectively individuals derive neuroprotective benefits from polyphenols and other antioxidants [127]. Future studies may determine which patients respond best to specific dietary patterns or supplements, leading to the development of psychobiotic or personalized antioxidant regimens that optimize brain health. Similarly, refining exercise prescriptions such as frequency, intensity, and modality may maximize antioxidant enzyme induction and mitochondrial adaptations, further protecting neurons from ROS-induced injury [130].

Gene editing tools, epigenetic modulators, and pharmacological agents targeting redox-sensitive pathways (Nrf2-ARE signaling) represent additional strategies for enhancing intrinsic antioxidant defenses. The concept of hormesis, where mild oxidative challenges can upregulate protective genes, may inspire therapies that precondition the brain against future insult [115,126]. For instance, low doses of certain phytochemicals or controlled fasting periods could trigger beneficial adaptive responses, strengthening neuronal resilience. However, translating these insights into widely applicable clinical interventions remains challenging. Nutritional and lifestyle changes require sustained adherence, cultural acceptance, and socioeconomic feasibility [131]. Moreover, neurodegenerative diseases often progress silently for years before clinical symptoms emerge, so interventions must be initiated early and maintained in the long term. Additionally, the interplay between oxidative stress, inflammation, and other pathological processes (protein misfolding, excitotoxicity) complicates efforts to isolate antioxidant interventions as a singular solution [132].

Ultimately, successful strategies will likely be multifactorial, combining antioxidant-rich diets, regular exercise, stress reduction techniques, sleep optimization, and potentially mild dietary restrictions to harness hormetic responses. Research must continue to refine biomarkers, identify individual responders, and test combination interventions in robust, well-controlled clinical trials. As understanding deepens, clinicians may one day prescribe a regimen of targeted foods, activity patterns, and lifestyle modifications as part of a personalized blueprint for neuroprotection, mitigating the destructive impact of oxidative stress on the aging brain. In conclusion, oxidative stress sits at the nexus of energy metabolism, aging, and neurodegeneration, highlighting the critical importance of effective antioxidant strategies for preserving cognitive function. By emphasizing whole food diets abundant in fruits, vegetables, legumes, whole grains, fish, and other nutrient-dense sources—paired with consistent physical activity, proper sleep, and stress management—individuals can bolster their intrinsic antioxidant defenses and reduce the cumulative burden of ROS on neuronal integrity. This holistic approach aligns well with the complexity of the central nervous system’s redox balance, offering hope that sustained lifestyle modifications will yield meaningful neuroprotective benefits and enhance quality of life, even as populations worldwide face the cognitive challenges of longer lifespans.

## 8. Neurogenesis and Synaptic Plasticity: Interventions and Insights

A healthy brain and the ability to adapt to new situations are underpinned by neurogenesis and synaptic plasticity. Strategies that aim to influence these processes, such incorporating more omega-3 fatty acids into one’s diet [133], flavonoids [134], and polyphenols [135], have demonstrated their potential to enhance neurogenesis and promote synaptic remodeling. Similarly, regular physical exercise, particularly aerobic and resistance training [136], has been shown to upregulate brain-derived neurotrophic factor (BDNF) and other neurotrophins that support neuronal survival and plasticity [137]. Emerging research also highlights the synergistic effects of combining targeted nutritional interventions with structured exercise protocols to optimize neuroplasticity [138,139]. Thus, these findings underscore the importance of a multifaceted approach, integrating lifestyle modifications to support brain health and mitigate cognitive decline.

As is known, the brain’s capacity to adapt and learn is almost invaluable and maintaining cognitive health should be essential [140]. These processes are influenced, as mentioned, by both exercise and nutrition, which individually and synergistically contribute to neural growth and connectivity. Then, exercise and nutrition have distinct, individual, and combined roles in enhancing neuroplasticity, followed by insights into integrated intervention programs [141].

### 8.1. The Role of Exercise

One of the best non-pharmacological ways to increase neurogenesis and synaptic plasticity is to exercise regularly [142,143]. Aerobic activities, such as running and cycling, have been shown to increase the production of BDNF, a key protein that supports the survival of neurons and facilitates synaptic remodeling (Figure 1) [144]. Concretely, Hao and collaborators recently pointed out findings supporting the notion that moderate-intensity physical activity correlates with the maintenance of cognitive function in middle-aged and older adults and may continue to confer this advantage in the future [145]. Additionally, intensity matters; Wu’s study suggests that mild and moderate physical activity may enhance cognitive ability, as opposed to intensive exercise. Thus, the specified intensity of physical activity may be more beneficial in attaining optimal cognitive enhancement, considering age and depressive condition [146]. Notably, the production of lactate is closely linked to the intensity of exercise; studies demonstrate that exercise-induced lactate acts as a signaling molecule to stimulate neurogenesis and enhance cognitive functions such as memory and executive processing. Although lactate was considered the “villain” in exercise physiology, recent research highlights its essential role in supporting cognitive processes, showing that it is crucial for optimal performance in tasks involving memory and executive functions [137].

Resistance training enhances neuroplasticity by reducing inflammation and promoting hormonal balance, including increased levels of insulin-like growth factor 1 (IGF-1) [147,148]. Beak et al. reported that dual-task resistance exercise is superior to resistance training in enhancing cognitive function in older persons with cognitive impairment. Both dual-task resistance training and resistance training enhance mood, alleviate depression, and increase functional fitness [149]. Physical activity and increased movement are already beneficial, even if it does not specifically focus on improving aerobic capacity or resistance. It improves cerebral blood flow, ensuring the delivery of oxygen and nutrients necessary for neuronal health [150,151]. Olivo et al. reported recently that the reduction in Grey Matter Blood Flow (GMBF) observed in younger adults immediately post-exercise is also evident in older adults and is associated with cardiovascular fitness, potentially reinforcing the connection between cardiovascular fitness and cerebrovascular reactivity in later life [152]. While there has been some success in elucidating the role of cerebral circulation as a moderator of aerobic exercise’s long-term effects on cognition, there is still a lack of clarity regarding the mechanisms by which exercise immediately affects cognition and cerebral perfusion, especially in the elderly. Taken together, these findings underscore the significant influence that exercise has on the structure and function of the brain [152].

### 8.2. Key Nutrients

Diet plays an equally critical role in modulating neurogenesis and synaptic plasticity [153]. Concretely, a defect in adult neurogenesis has been suggested as a prevalent characteristic in various age-related neurodegenerative disorders [84]. The administration of flavonoids is presently seen as a potentially advantageous approach for mitigating abnormalities associated with brain aging, particularly the reduction in adult neurogenesis [154]. A balanced diet has typically been associated with cognitive improvements, such as a reduction in neuroinflammation [134], and consequently, a lower likelihood of anxiety [155], stress, or simply academic performance improvements in students [156]. In fact, compliance with a nutritious regimen, such as the Mediterranean Diet (MD), may provide advantageous outcomes for university students, enhancing their academic performance, quality of life, and overall mental and physical health [157]. Also, a plant-based diet and its bioactive compounds have been documented to confer health benefits, not only cognitive but also multifactorial ones, such as protection against cancer, cardiovascular diseases, and diabetes, as well as benefits for individuals with gastrointestinal, immune, and neurodegenerative disorders [158,159].

Regarding these, flavonoids, found in fruits, vegetables, and cocoa, have been also shown to enhance BDNF expression and reduce oxidative stress, thereby supporting these processes [160]. Similarly, polyphenols and curcumin exhibit anti-inflammatory and neuroprotective properties, which contribute to improved synaptic plasticity [161,162]. Also, omega-3 fatty acids, particularly docosahexaenoic acid (DHA), are essential for maintaining neuronal membrane integrity and promoting synaptic formation [163,164]. For instance, Roberts and collaborators found that childhood undernutrition correlates with enduring cognitive damage. Contrary to prevailing beliefs, supplementary feeding for 23 weeks may enhance executive function, cerebral health, and nutritional status in at-risk young children residing in low-income nations [165]. This proves that an adequate intake of vitamins such as B12, D, and E is also linked to better cognitive outcomes by supporting myelination, reducing neurodegeneration, and mitigating oxidative damage [166,167]. McGeown’s study showed that antioxidants, branched-chain amino acids, and ω-3 polyunsaturated fatty acids have demonstrated the most promising preclinical results for modifying neuroprotective effects following traumatic brain injury [168].

Therefore, mental health is essential to a person’s overall health, and vice versa: leading an unhealthy lifestyle can have a negative impact on one’s mental health. To keep one’s brain and body in peak condition, it is recommended to include both macro- and micronutrients in a varied and balanced diet, in addition to leading a healthy lifestyle. Given the effects of stress on the brain over the lifespan and the fact that the human population is aging at a rapid pace, this approach is more important than ever. The significance of dietary interventions for cognitive health is underscored by the fact that these nutrients work together to produce a biochemical milieu that is favorable to brain growth and repair [169].

### 8.3. Synergy of Exercise and Nutrition

**Figure 2:**
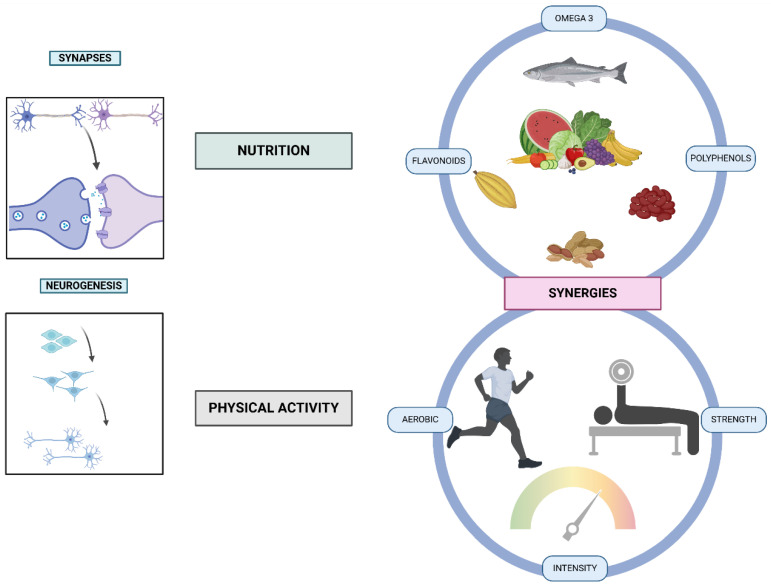
Neurogenesis and synaptic plasticity boosted by synergy interventions.

Emerging research underscores the synergistic effects of combining exercise and nutrition to optimize neurogenesis and synaptic plasticity [170]. Intervention programs that integrate structured aerobic or resistance exercise with a nutrient-dense diet rich in omega-3 fatty acids, flavonoids, and antioxidants have demonstrated superior outcomes in cognitive performance and mental health (Figure 2) [171]. For instance, exercise-induced BDNF upregulation is potentiated by DHA supplementation, leading to enhanced synaptic connectivity [171]. Additionally, the simultaneous use of creatine nitrate and caffeine enhanced cognitive function, especially in activities involving cognitive interference, without affecting short-term exercise performance [172]. Concretely, Komulainen and collaborators reported that a blend of at least moderate-intensity aerobic exercise and a nutritious diet may enhance cognitive function in older adults. Studies such as Wang’s have suggested that the influence of nutrition and exercise on cognitive function is receiving heightened scrutiny. The advantages of exercise for cognitive function and brain plasticity are extensive, and future study may investigate the effectiveness of specific dietary protocols during physical activity in conjunction with nutrition to mitigate cognitive decline [173]. Thus, programs tailored to include both elements have shown promise in slowing cognitive decline, for instance, in aging populations and improving neuroplasticity in individuals with neurodegenerative disorders [53,174].

The interaction between specific types of exercise and nutrients shows significant synergies in optimizing cognitive function and neuroplasticity. Aerobic exercise combined with omega-3 fatty acids, such as DHA and EPA, has been shown to improve brain health by reducing inflammatory markers and increasing mitochondrial density and mitochondria-regulated apoptotic signaling [175]. Likewise, studies in patients with mild cognitive impairment suggest that aerobic exercise combined with omega-3 supplementation may preserve gray matter volume in critical brain regions related to Alzheimer’s disease [176]. On the other hand, B vitamins, essential in neuronal metabolism, can complement the benefits of resistance training by preventing muscle loss and supporting neurotransmitter synthesis. Although evidence is limited, supplementation with B vitamins along with resistance exercise has been shown to preserve serum B12 levels and promote neuroplasticity in older adults [175]. This integrative approach highlights how different combinations of nutrients and exercises can be personalized to maximize benefits in cognitive and physical health, highlighting the importance of strategies tailored to individual characteristics.

In contrast, Bischoff-Ferrari et al. studied and reported that in adults aged 70 years or older without significant comorbidities, interventions involving vitamin D3, omega-3 fatty acids, or a strength-training exercise regimen did not yield statistically significant variations in systolic or diastolic blood pressure, nonvertebral fracture incidence, physical performance, infection rates, or cognitive function [177]. Also, a 12-month multicomponent exercise training and supplementation regimen shown no meaningful impact on cognition in males undergoing treatment [178]. Formica et al., in line with these results, reported that in healthy community-dwelling older adults engaging in resistance-based exercise training three times per week, those who consumed lean red meat according to current Australian dietary guidelines did not observe any significant supplementary benefits in the primary outcome measures of muscle mass, strength, or cognitive function [179]. Personalized approaches, which account for individual dietary needs and physical fitness levels, are particularly effective in maximizing the benefits of these interventions [180]. The timing of nutrient intake involves strategic intake at specific times around exercise to optimize exercise adaptation, including improvements in muscle strength, body composition, and physical performance [181]. Along these lines, recent studies have shown that physical exercise increases levels of neurotrophic factors such as BDNF, a key neurotrophin in neuroplasticity [182]. Although specific research on the timing of nutrient intake and its direct effect on neuroplasticity is limited, evidence suggests that adequate nutrition around exercise may enhance cognitive and neurological benefits. Therefore, a balanced intake of nutrients both before and after exercise is recommended to maximize its positive effects on brain health.

The synergistic effects of exercise and diet offer a potential strategy to boost neurogenesis and synaptic plasticity. Brain health and resilience can be optimized throughout life through this interaction. In contrast, there have been cases where prior studies did not find statistically significant variations in the results of interventions. This emphasizes the need for higher-quality research to establish and standardize protocols that successfully combine dietary and physical activity plans.

## 9. Wearable Bioelectronics in Monitoring Mental Health

Mental illness, regardless of being medically diagnosed or undiagnosed, impacts a significant segment of the population [183]. It is a substantial contributor to widespread impairment, and if inadequately addressed, it may result in serious emotional, behavioral, and physical health issues [184]. Most mental health research projects prioritize treatment, while fewer resources are allocated to technological solutions for mental health problems. Nowadays, wearables are electronic devices and “wearable” gadgets, meaning they are worn on some part of the body to perform a specific task. Clothing, watches, glasses, bracelets or rings have been categorized in the market into five major groups: health, sports and wellness, entertainment, industry, or military (Figure 2) [185]. Today, there is no doubt that wearable technology helps patients monitor numerous aspects of their health, such as heart rate, sleep quality, blood sugar levels, and even depression [186].

Wearable sensors provide numerous benefits compared to conventional mental health evaluation approaches, such as ease, cost-efficiency, and the capacity to collect data in real-world environments [187,188]. Their ability to record information about anxiety, stress, and panic attacks is tested. The current market sensors are described in detail, along with their effectiveness in providing data that can be linked to the health concerns mentioned earlier. There is a lot of change happening in the wearable industry right now, and the products that are already on the market are good enough to give significant data that machine learning algorithms can use for any kind of illness, not just mental health [189]. For instance, Alshurafa et al. developed “Wanda”, a predictive system for reducing cardiovascular disease (CVD) risk factors. Over six months, users received technology-based support, with the system achieving an F-score of 0.92. It effectively identified behaviors in the first month, using activity, blood pressure, and questionnaires, to determine which participants would benefit most from remote health monitoring [190]. Similarly, Aranki et al. developed a smartphone-based system for real-time monitoring of vital signs and cardiovascular symptoms during physical activity in heart disease patients, with potential applications for diabetes, hypertension, and other chronic conditions [191].

### 9.1. Monitoring Mental Health

Regarding mental health, it has been demonstrated that wearables and mobile apps offer promising tools to detect early deterioration in young people, enabling timely intervention and reducing delays in accessing mental health services [192]. For instance, Osmani et al. conducted a study using smartphones to detect episodes and monitor behavioral changes in patients with bipolar disorder [193]. There are many more advancements and studies aimed at monitoring and preventing these diseases [194]. Reinertsen et al. used a support vector machine (SVM) classifier to identify schizophrenia patients from HRV and accelerometer data [195]. A recent study by Greco et al. (2018) suggested that HRV is also a great variable for detecting subclinical depression in 60 undergraduate students [196]. Prince et al. (2018) analyzed data from the largest cohort of Parkinson’s patients to date (312 patients and 236 controls) using smartphone-based finger tapping and memory tests. This study identified digital biomarkers predictive of Parkinson’s severity, enabling remote and quantitative patient monitoring [197]. Thus, findings suggest that mental health monitoring is practicable [198]. However, the evidence also reported that while wearable technology and digital mental health interventions (DMHIs) offer significant potential to revolutionize mental health care, they also pose unique challenges that require careful evaluation and strategic implementation [186]. If we needed to identify the most commonly used devices, Robinson et al. highlights that electrodermal activity (EDA/GSR/SC/Skin Temperature), physical activity, and heart rate (HR) are the most commonly assessed biometrics. Although smartwatches are increasingly prevalent in the market, fitness trackers provide the greatest public value benefit of GBP 513.9 million, presumably owing to superior user retention rates [199]. Non-invasive wearable devices use advanced machine learning-based algorithms to interpret external physiological data in terms of internal processes such as neuroplasticity and neurogenesis. For example, heart rate variability (HRV) can be correlated with autonomic nervous system activity and its impact on the HPA axis, linked to the release of neurotrophins such as BDNF [200]. Cortisol can provide insights into the state of chronic stress and its relationship to neuroinflammation, a key factor in the inhibition of synaptic plasticity [201]. The combined use of physiological data and big data-based algorithms allows us not only to monitor the current state of the individual, but also to predict patterns of neuronal recovery and adaptability.

### 9.2. Key Innovations

**Figure 3:**
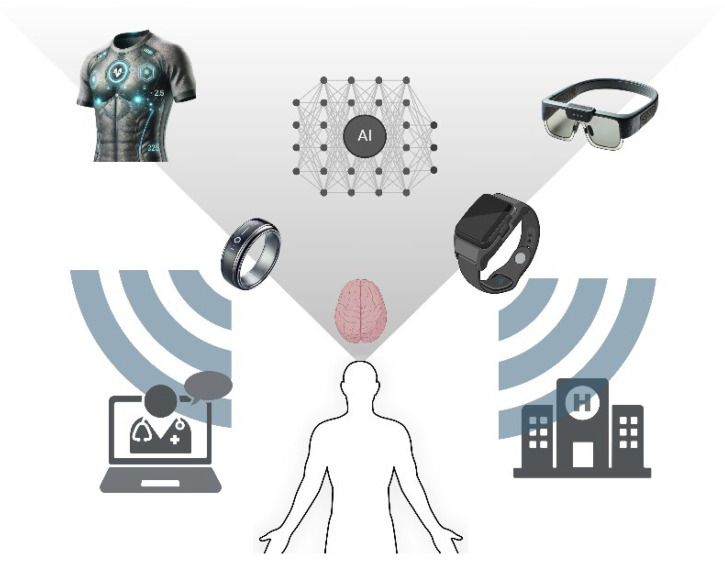
Wearable bioelectronics improving mental health.

Recent advancements include the integration of artificial intelligence (AI) for predictive analytics, allowing wearable devices to identify early signs of mental health conditions before they manifest clinically (Figure 3) [202]. For example, predictive models using longitudinal data from wearable devices can forecast depressive episodes or heightened anxiety periods [203]. Additionally, wearable devices equipped with haptic feedback mechanisms are being explored to deliver biofeedback therapies, helping users manage stress in real time. AI offers significant prospects for enhancing autism spectrum disorder (ASD) management; however, realizing these advantages necessitates a collaborative endeavor among engineers, clinicians, ethicists, and politicians to create AI solutions that are both novel and ethically sound, equitable, and universally advantageous [204].

Despite their potential, wearable bioelectronics for mental health monitoring face challenges such as ensuring data privacy, maintaining device accuracy, and overcoming user adherence barriers. Data security is paramount given the sensitive nature of mental health information, necessitating robust encryption and secure communication protocols [205]. Wearable devices are supported by advanced machine learning models that allow for the interpretation of physiological data in the context of neurocognitive health. Algorithms such as Random Forest, Support Vector Machine (SVM), and XGBoost have proven effective in processing data such as heart rate variability (HRV), sleep patterns, and activity levels. These systems integrate these metrics into classifiers capable of predicting changes in neuroplasticity and other mental health outcomes. For example, a recent system used these algorithms combined into a voting classifier to predict mental health-related outcomes in real time, achieving high accuracy in its predictions [206].

Opportunities abound in expanding the accessibility of these devices to underserved populations. Low-cost, scalable solutions can democratize mental health monitoring, bridging gaps in traditional healthcare systems. Moreover, interdisciplinary collaborations between technologists, psychologists, and medical professionals are vital for designing user-centric devices that meet clinical needs [207].

In conclusion, wearable bioelectronics represent a significant leap in mental health monitoring, combining technological innovation with clinical application to improve outcomes [187]. Future developments in this field will likely focus on enhancing device capabilities, integrating multimodal data sources, and fostering user trust to maximize adoption and effectiveness.

## 10. Bioengineering and Personalized Neuro-Nutritional Strategies

The convergence of bioengineering innovations and personalized medicine presents unprecedented opportunities for tailoring neuro-nutritional strategies to individual needs, addressing both cognitive enhancement and mental health optimization. Advancements in genomics, metabolomics, and wearable bioelectronics enable the identification of individual physiological and neurochemical profiles, which can inform precise interventions aimed at optimizing brain function and psychological resilience [208].

Recent breakthroughs in bioengineering have facilitated the design of advanced devices and platforms capable of monitoring and modulating the intricate interactions between brain and body systems. These innovations leverage interdisciplinary advances in materials science, nanotechnology, and computational biology to provide unprecedented precision in both diagnostics and intervention. For instance, wearable biosensors, integrated with artificial intelligence (AI) algorithms, can analyze real-time data on a wide array of physiological parameters such as glucose levels, cortisol secretion, neural activity, and even gut microbiota metabolites [209]. By combining these data streams, these systems create a comprehensive profile of an individual’s physiological and neurochemical state. The ability to monitor these biomarkers in real time enables a dynamic assessment of an individual’s response to dietary, pharmacological, and physical interventions. Such insights can inform actionable feedback loops, allowing clinicians and users to adjust strategies in real time for optimized outcomes. For example, wearable electroencephalography (EEG) devices have been adapted to monitor neural activity during cognitive tasks, providing feedback on brain performance and stress levels, which can be linked to dietary modifications or relaxation techniques [210].

Microfluidic systems, another pivotal advancement, provide a non-invasive and efficient means of analyzing biomarkers through fluids like sweat, saliva, or interstitial fluid. These compact platforms, often the size of a smartphone, employ advanced biomarker detection technologies such as enzyme-linked assays, electrochemical sensors, and fluorescence detection to monitor critical neurochemical signals, including serotonin, dopamine precursors, and cortisol [211]. By capturing these signals, microfluidic devices offer insights into neurochemical imbalances that underline mood disorders, anxiety, and cognitive impairments. This information can be used to tailor interventions, such as nutrient-specific supplementation or therapeutic regimens aimed at restoring neurotransmitter equilibrium. Furthermore, bioelectronic medicine has emerged as a transformative field, with implantable devices capable of directly modulating neural circuits to treat neurodegenerative diseases or enhance cognitive functions [212]. Devices like vagus nerve stimulators and closed-loop neuromodulation systems provide precise control over neurophysiological states, enabling therapeutic interventions tailored to individual needs. These systems, often coupled with AI, can predict and respond to neural signals in real time, offering a new dimension to personalized mental health care [213].

The convergence of bioengineering and machine learning has also enhanced the accuracy and applicability of neuro-nutritional interventions. Predictive models trained on vast datasets from diverse populations can identify trends and predict outcomes, enabling practitioners to design precision interventions based on an individual’s genetic predispositions, metabolic profile, and lifestyle factors [214]. For example, combining genetic data with wearables that track dietary intake and physical activity can help predict susceptibility to conditions like cognitive decline or metabolic disorders, leading to preemptive interventions. In addition to monitoring, advancements in responsive biomaterials are pushing the boundaries of personalized neuro-nutrition. Smart hydrogels and nanoparticles designed for targeted drug and nutrient delivery can respond to changes in pH, temperature, or enzymatic activity in the body [215]. These technologies can deliver specific nutrients or medications to targeted brain regions, bypassing systemic effects and maximizing efficacy. Together, these integrative approaches signify a paradigm shift in how we understand and manage the interplay between nutrition, exercise, and mental health. They provide a foundation for the next generation of bioengineered solutions, paving the way for precise, individualized strategies that optimize cognitive function and psychological resilience. Future research must aim to address the scalability and accessibility of these technologies, ensuring equitable implementation across diverse populations [216].

Personalized nutrition, rooted in the principles of precision medicine, represents a transformative approach to optimizing cognitive health and psychological resilience. By tailoring dietary recommendations and interventions to an individual’s genetic, biochemical, and lifestyle profile, personalized neuro-nutritional strategies hold the promise of mitigating cognitive decline, enhancing mental health, and preventing neurodegenerative diseases [208]. The advent of genomic technologies has made it possible to design diets that align with an individual’s genetic predispositions. Single-nucleotide polymorphisms (SNPs), which influence nutrient metabolism and brain health, serve as valuable markers for tailoring interventions [217]. For example, individuals carrying the APOE ε4 allele, associated with a heightened risk of Alzheimer’s disease, may benefit from diets enriched in omega-3 fatty acids such as DHA. DHA, a critical component of neuronal membranes, supports synaptic plasticity and reduces neuroinflammation. Similarly, variations in genes like MTHFR, which influence folate metabolism, can guide supplementation with bioavailable forms of folate to support neurotransmitter synthesis and prevent cognitive impairment [218]. Additionally, nutrigenomics, an emerging field that explores the interaction between diet and gene expression, has revealed insights into the role of diet in modulating epigenetic markers. Dietary components such as polyphenols, omega-3 fatty acids, and B vitamins have been shown to influence DNA methylation and histone modification, enhancing the expression of genes involved in neuroplasticity and stress resilience. These insights enable the design of diets that not only address genetic predispositions but also actively optimize gene function for brain health [219].

Advancements in metabolomics and proteomics have revolutionized the ability to detect nutrient deficiencies or imbalances at an individual level. Through the analysis of biomarkers such as amino acids, fatty acid profiles, and micronutrient levels, clinicians can identify specific deficiencies and design interventions with precision [220]. For instance, low serum levels of vitamin D, a critical modulator of synaptic plasticity and neurogenesis, can be addressed through targeted supplementation. Similarly, magnesium deficiency, which is linked to heightened stress responses and reduced cognitive function, can be rectified through dietary adjustments or supplements. Biomarker-guided strategies also extend to the regulation of neurotransmitter precursors [221]. For example, ensuring adequate intake of tryptophan and tyrosine precursors to serotonin and dopamine, respectively, can improve mood regulation and cognitive performance. These amino acids can be delivered through functional foods or supplements designed to align with an individual’s metabolic profile [222]. The role of the gut–brain axis in cognitive and emotional health has gained significant attention, with growing evidence highlighting the impact of the gut microbiota on neurotransmitter production and neuroinflammation. Personalized neuro-nutritional strategies increasingly incorporate probiotics and prebiotics to modulate the gut microbiome, fostering the production of beneficial metabolites such as SCFAs that influence brain function [223]. For example, specific strains of probiotics, such as Lactobacillus and Bifidobacterium, have been shown to enhance serotonin synthesis in the gut and reduce systemic inflammation, contributing to improved mood and cognitive clarity. Prebiotics, such as inulin and resistant starch, serve as substrates for beneficial gut bacteria, promoting a microbiome environment conducive to brain health. Personalized approaches may include the incorporation of these elements into daily diets, informed by microbiome analyses that identify individual microbial compositions [224].

The use of functional foods enriched with bioactive compounds and advanced supplement formulations further enhances the personalization of neuro-nutrition. For instance, polyphenol-rich foods like berries and green tea can be tailored to enhance cognitive performance through their antioxidant and anti-inflammatory properties. Similarly, fortified foods with added omega-3s, vitamins, and minerals provide targeted benefits for brain health [225]. In combination with wearable technologies and AI-driven platforms, these interventions can be continuously monitored and adjusted based on real-time feedback, ensuring their efficacy. The integration of data from wearable devices, metabolomic profiles, and genetic analyses into comprehensive nutrition plans represents the pinnacle of precision neuro-nutritional care. While the potential of personalized neuro-nutritional interventions is immense, challenges remain in their implementation [226]. Cost and accessibility of advanced diagnostic technologies, ethical considerations in the use of genetic data, and the need for large-scale clinical validation of tailored interventions are critical areas for future focus. Nonetheless, as bioinformatics and machine learning continue to evolve, the scalability and affordability of these personalized strategies are expected to improve, bringing transformative benefits to a wider population [227]. Through the integration of genetic, biochemical, and microbiome data, personalized neuro-nutritional interventions provide a powerful framework for optimizing brain health and resilience. By addressing individual variability in nutrient requirements and leveraging the interplay between diet and the nervous system, these approaches promise to redefine mental health care and cognitive enhancement in the era of precision medicine.

## 11. Integrating Exercise with Nutrition for Stress Resilience

The phenomenon of stress resilience is defined as the body’s ability to adapt, resist, and recover from stressors, thereby minimizing psychophysiological impact. This capability of the organism is influenced by a combination of genetic, biological, and environmental factors and plays an important role in preventing stress-related disorders, such as anxiety and depression [228]. In a psychophysiological context, stress resilience is closely involved in the effective regulation of the hypothalamic–pituitary–adrenal (HPA) axis [229]. The HPA axis controls the release of cortisol and other stress-related hormones. Cortisol, known as the stress hormone, is essential for maintaining homeostasis, but its chronic overproduction can result in adverse effects such as neuronal damage, decreased brain plasticity, and predisposition to disorders such as depression or chronic anxiety [230]. Dysfunction of the HPA axis can lead to chronic stress and contribute to the development of mental health disorders.

Scientific evidence demonstrates that regular physical activity, at moderate to vigorous levels, is an effective intervention to achieve resilience against stress [231]. Physical activity has been shown to regulate cortisol levels and stimulate the release of endorphins and serotonin, which are related to mood stabilization and emotional well-being [232]. Moreover, physical activity promotes the release of neurotrophic factors such as BDNF, a neurotrophic protein essential for brain plasticity [233]. This physiological process is key in brain plasticity, as it stimulates the formation of new neuronal connections, improving learning and memory [234]. Aerobic exercises such as running, walking, and cycling improve cerebral vascularization, increase oxygen delivery to the brain and attenuate the release of cortisol under stress [235]. Although moderate physical exercise has high psychophysiological benefits, new lines of research have shown that high-intensity exercise, although it can initially increase cortisol, provides high resilience benefits against stress [236]. Researchers suggest that intense exercise stimulates adaptive immune responses and thus improves the constant processes of homeostasis of the organism.

In parallel, nutrition plays a vital role in supporting resilience to stress by influencing various neurobiological processes [237]. Omega-3 fatty acids, especially DHA and EPA, are essential to mitigate neuroinflammation by inhibiting proinflammatory cytokines and promoting the production of resolvins [238]. These fatty acids enhance synaptic plasticity, favoring cognitive processes such as memory and learning. Micronutrients like B vitamins contribute to the synthesis of neurotransmitters such as serotonin and dopamine, which are essential for emotional regulation and neuronal protection [239]. Another micronutrient, magnesium, has been shown to be a modulator of neuronal excitability by regulating NMDA channels and decreasing the hyperactivation of the HPA axis, thus stabilizing the stress responses [240]. Additionally, polyphenols such as resveratrol and catechins exhibit potent antioxidant properties, promoting neurogenesis in the hippocampus and protecting against cognitive decline [241].

Combining regular physical activity with targeted nutritional strategies creates powerful synergistic effects by acting on shared physiological pathways to build resilience [242]. Together, these habits help reduce systemic inflammation, balance reactive oxygen species (ROS), and support neurogenesis, fostering a protective environment for the brain [243]. This holistic approach also influences the gut–brain axis, a fascinating and emerging area in stress resilience research. Including fermentable fibers in the diet stimulates the production of short-chain fatty acids (SCFAs), which play a role in neuronal signaling and help maintain the balance of the HPA axis [244]. Physical exercise complements these effects by improving microbial diversity, reinforcing the intestinal barrier, and enhancing gut–brain communication. Both interventions reduce oxidative damage, regulate cortisol levels, and promote a healthy response to stress. Furthermore, improved circulation from exercise enhances the delivery of essential nutrients and metabolites to the brain, amplifying the anti-inflammatory and neuroprotective benefits of a nutrient-rich diet [245].

This combined model of physical activity and nutrition not only builds resilience to stress, but also supports overall mental and physical health. By focusing on mechanisms such as HPA axis regulation and oxidative balance, this approach provides a comprehensive framework for adapting to stress. It also holds promise for preventing stress-related disorders and slowing neurodegenerative processes, laying a solid foundation for long-term well-being.

## 12. Translational Approaches to Address Neurodegeneration

Nowadays, neurodegenerative diseases such as Alzheimer’s, Parkinson’s, and amyotrophic lateral sclerosis (ALS) represent one of the challenges in terms of intervention for global health systems. In addition, the world is experiencing progressive aging, with an increasing number of elderly people with neurodegenerative problems. These pathologies are characterized by the gradual and irreversible loss of neurons, a degradation of cognitive and motor abilities, and, in turn, a marked deterioration of the quality of life [246]. In this context, interdisciplinary approaches are essential that not only seek to treat the symptoms but also help predict and manage the disease [247]. The development of tools for early diagnosis has been studied as a key aspect to allow intervention before neurophysiological damage is irreversible. In this sense, biomarkers have been positioned as fundamental elements for the early detection of these pathologies [248]. For example, Alzheimer’s detection models have been developed with proteins such as amyloid beta and tau protein obtained through cerebrospinal fluid (CSF). Early detection models have also been studied through the Neurofilament Light Chain (NfL) in blood or plasma [249]. These evaluations are less invasive and can monitor the progression of different neurodegenerative disorders. Other invasive methods studied include molecular imaging technologies, such as positron emission tomography (PET) and magnetic resonance imaging (MRI), which have consolidated their ability to visualize pathological processes in the brain noninvasively [250].

Beyond diagnosis, prevention and management of these diseases have begun to integrate strategies based on nutrition and physical exercise, which have shown a significant impact on neuroprotection [245]. A diet rich in antioxidants, such as polyphenols, vitamins E and C, and omega-3 fatty acids, has demonstrated its ability to reduce oxidative stress and neuroinflammation, two key processes in the progression of neurodegenerative diseases [251]. Physical exercise, particularly aerobic and resistance activities, stimulates the release of brain-derived neurotrophic factor (BDNF), a protein essential for synaptic plasticity and neurogenesis [232]. These interventions, when implemented together, have a synergistic effect that promotes a neuroprotective environment, significantly reducing the risk of progression of these disorders.

In the field of emerging technologies, bioengineering and nanotechnology are transforming the research and treatment of neurodegenerative diseases. For example, the development of nanoparticles allows the targeted delivery of antioxidants and neuroprotectors to affected areas of the brain, increasing their efficacy and reducing adverse effects [251]. Portable sensors offer an innovative tool to monitor biomarkers related to oxidative stress and neuroinflammation in real time, providing essential data to personalize therapeutic interventions [252]. Additionally, genetic editing using techniques such as CRISPR-Cas9 is opening new possibilities to correct mutations associated with these diseases, bringing the possibility of curative therapies closer [253].

Looking to the future, the integration of these technological and therapeutic advances requires an interdisciplinary approach that combines biomarkers, advanced technologies, and lifestyle-based strategies such as nutrition and exercise [248]. This model will not only allow the development of personalized treatments tailored to the genomic, epigenetic, and metabolic profiles of each patient, but will also optimize the effectiveness of interventions. International collaboration will be essential to ensure that these advances equitably reach the most vulnerable populations, bridging the gap between scientific discoveries and their clinical application. Similarly, investment in translational research will be key to converting scientific findings into practical solutions that improve the quality of life of patients and reduce the economic burden that these diseases represent for health systems.

Finally, the development of integrative and multidimensional strategies has the potential to transform the paradigm of management of neurodegenerative diseases. By combining advanced technologies, early diagnosis and lifestyle changes, we are paving the way to a future where prevention, personalized treatment and neuroprotection are fundamental pillars. This approach not only offers hope to the millions of people affected by these devastating conditions, but also to the many people who are affected by them.

## 13. Practical Applications: Bridging Science and Everyday Health

The findings from this paper offer actionable insights that can be directly integrated into health and wellness strategies. By emphasizing the synergistic effects of neuro-nutrition and exercise, healthcare providers, educators, and fitness professionals can develop holistic programs tailored to individual needs. Key applications include:

1. Personalized Intervention Design: Leveraging wearable bioelectronics, individuals can monitor physiological and cognitive markers to fine-tune their nutritional and exercise regimens. For example, using devices to track heart rate variability and stress biomarkers enables real-time adjustments to maintain optimal mental and physical health.
2. Mental Health Optimization: The integration of exercise and targeted nutritional strategies—such as omega-3 fatty acids, polyphenols, and B vitamins—can serve as complementary therapies for managing stress, anxiety, and depression. Practitioners can design structured interventions combining dietary guidance with aerobic or resistance training programs.
3. Cognitive Enhancement Programs: For populations ranging from students to aging adults, incorporating brain-boosting nutrients alongside regular physical activity can enhance learning, memory, and executive function. This is particularly relevant for addressing age-related cognitive decline and neurodegenerative diseases.
4. Corporate Wellness Initiatives: Organizations can incorporate these insights into workplace wellness programs, promoting cognitive resilience and emotional well-being through guided exercise sessions and on-site nutritional support.
5. Community Outreach and Education: Public health campaigns can disseminate simplified guidelines on combining balanced diets with exercise to optimize mental health and cognitive performance, empowering individuals to take proactive steps toward well-being.

By translating scientific advancements into practical tools and programs, this interdisciplinary approach has the potential to revolutionize preventative health care, promoting resilience against cognitive decline and enhancing overall quality of life.

The integration of neuro-nutrition and exercise can significantly influence various domains of health and well-being. Educational systems can incorporate these principles into school and university curricula, fostering lifelong habits of mental and physical health through interactive workshops and courses that include nutrition basics for brain health, exercise modules emphasizing cognitive benefits, and real-life case studies demonstrating the impact of combined interventions. Students can actively participate by tracking their dietary habits and physical activity, correlating these with their cognitive performance. Athletes and performance-driven professionals can greatly benefit from tailored interventions that combine exercise with neuro-nutrition, such as timing omega-3 supplementation alongside recovery sessions to enhance synaptic plasticity, leveraging high-intensity interval training to maximize neurotrophic factor release, and utilizing personalized meal plans that optimize neurotransmitter synthesis during critical training periods.

In the realm of mental health care, structured programs can integrate cognitive–behavioral therapy with lifestyle interventions. Clinicians can design nutritional protocols to enhance serotonin and dopamine pathways through amino acid-rich foods like tryptophan and tyrosine, alongside exercise plans targeting anxiety and depression symptoms by reducing cortisol levels and boosting endorphin production. Similarly, for patients managing chronic diseases such as diabetes, cardiovascular conditions, or neurodegenerative disorders, combining neuro-nutrition and exercise can improve insulin sensitivity through dietary polyphenols and aerobic exercise, reduce systemic inflammation with anti-inflammatory diets like the Mediterranean diet coupled with physical activity, and slow cognitive decline by increasing hippocampal neurogenesis.

Workplaces can adopt practices that improve cognitive performance and creativity among employees by introducing active breaks involving light aerobic exercises to enhance blood flow and BDNF levels, offering access to nutritious snacks such as nuts, berries, and omega-3-rich foods to sustain cognitive clarity, and conducting workshops to build awareness of the connection between physical activity, nutrition, and mental well-being. Emerging health technologies also provide opportunities for integrating these interventions. AI-driven apps can monitor dietary intake and physical activity, offering actionable insights, while augmented reality tools can guide physical activities and mindfulness exercises targeting cognitive health. Non-invasive wearables combined with telehealth services can provide real-time feedback on stress and mental health markers, enabling personalized interventions.

Community-based initiatives can amplify the societal impact by promoting the role of exercise and nutrition in preventing common mental health issues. Organizing fitness challenges integrated with dietary guidance, forming partnerships with local gyms and nutritionists to create accessible programs, and leveraging social media and mobile platforms to disseminate evidence-based tips for daily habits are just a few examples. These strategies not only address individual health needs but also provide scalable solutions for global challenges like stress, depression, and cognitive decline, creating a holistic framework that bridges cutting-edge scientific research with actionable, everyday practices.

## 14. Conclusions

The synergy between neuro-nutrition and exercise represents a transformative approach to optimizing cognitive function and mental health. This comprehensive review highlights the intricate biological mechanisms underlying their combined effects, including enhanced neuroplasticity, increased neurogenesis, and the modulation of key neurotrophic and hormonal factors. By integrating targeted dietary strategies with structured physical activity, it is possible to mitigate the impacts of aging, stress, and neurodegenerative diseases, while also enhancing cognitive resilience, emotional regulation, and overall quality of life.

The translational potential of this interdisciplinary approach extends beyond individual health, offering opportunities for innovative applications in education, sports, healthcare, and public health initiatives. From personalized interventions enabled by wearable technology to community-based programs promoting healthy lifestyles, the practical applications of this research provide a roadmap for addressing contemporary challenges such as the rising prevalence of anxiety, depression, and cognitive decline.

### 14.1. Limitations

While this review highlights the synergistic effects of nutrition and exercise on neuroplasticity and cognitive performance, it is important to acknowledge certain limitations. One of the main concerns is the generalizability of these findings across diverse populations. Genetic variations can significantly influence the metabolism and bioavailability of key nutrients, as well as individual responses to exercise-induced neuroplasticity. For instance, polymorphisms in genes regulating brain-derived neurotrophic factor (BDNF), dopamine metabolism, and inflammatory pathways may alter the cognitive benefits of dietary and exercise interventions. Additionally, lifestyle factors such as sleep quality, stress levels, habitual physical activity, and overall diet composition can modulate the effects of these interventions.

Sociocultural influences also play a critical role in shaping dietary habits and physical activity behaviors, which may impact the efficacy of combined interventions. Differences in food availability, cultural dietary preferences, socioeconomic status, and access to structured exercise programs can introduce variability in the observed outcomes. Therefore, caution should be exercised when extrapolating these findings to broader populations, and future research should focus on personalized approaches that consider genetic, environmental, and sociocultural determinants of neurocognitive health. Longitudinal and interventional studies in diverse cohorts are needed to further validate the applicability of these mechanisms across different demographic groups.

### 14.2. Future Studies

Future research should aim to elucidate critical molecular pathways, such as the BDNF signaling cascade, which underlies synaptic plasticity and neurogenesis; the Nrf2 pathway, which regulates antioxidant defense and reduces oxidative stress; and the mTOR pathway, which influences cellular energy metabolism and neuroprotection. Additionally, investigating the long-term effects of combined interventions on these pathways and others, such as those related to gut–brain axis metabolites like short-chain fatty acids (SCFAs) and tryptophan-derived serotonin, is essential. Developing advanced tools for real-time monitoring of these molecular dynamics and optimizing brain health will help bridge the gap between science and everyday life.

More specifically, key pathways for future investigation include:

− *BDNF-TrkB Signaling Pathway*: Future studies should explore how neuro-nutrition and exercise can optimize BDNF expression to enhance neuroplasticity and cognitive resilience. The impact of tailored interventions on TrkB receptor activation and downstream effects warrants further research.
− *PI3K/Akt/mTOR Pathway*: Research should focus on the combined effects of exercise and specific dietary components, such as omega-3 fatty acids and polyphenols, in modulating this pathway to promote neuronal growth and prevent cognitive decline.
− *ERK/CREB Pathway*: Investigating the role of dietary antioxidants and structured exercise programs in enhancing CREB-mediated memory formation and synaptic remodeling could provide new insights into cognitive optimization strategies.
− *Nrf2-Antioxidant Response Pathway*: Further studies should assess how antioxidant-rich diets and exercise-induced mild oxidative stress synergistically activate Nrf2, potentially reducing neuroinflammation and age-related cognitive decline.
− *Gut–Brain Axis and Neuroinflammation*: The influence of gut microbiota on brain health via metabolites like SCFAs and serotonin precursors requires deeper investigation, with an emphasis on dietary and exercise interventions tailored to individual microbiome profiles.

Neuro-nutrition and exercise-based strategies have the potential to revolutionize preventive and therapeutic practices, paving the way for a healthier, more cognitively resilient society.

## Figures and Tables

**Table 1:** State-of-the-art mechanisms of neuroplasticity: interactions between nutrition and exercise.

Key Aspect	Cellular and Molecular Mechanisms	Synaptic and Structural Plasticity	Role of Nutrition (Nutrients, Dietary Patterns)	Role of Exercise (Type, Intensity, Frequency)	Evidence (Preclinical, Clinical, Imaging)
Neurotrophic Factors (BDNF, NGF)	**Molecular:** Exercise and omega-3 fatty acids, polyphenols (resveratrol, catechins), and flavonoids enhance BDNF, IGF-1, and NGF expression. Mechanisms involve the AMPK–PGC-1α–FNDC5 pathway leading to BDNF upregulation. BDNF signaling via TrkB activates downstream MAPK/ERK and PI3K/Akt cascades, enhancing synaptic protein synthesis [6,56].	**Synaptic Plasticity:** Increased BDNF levels facilitate long-term potentiation, synaptogenesis, dendritic spine density, and spine maturation in hippocampal and cortical circuits. This results in improved cognitive functions (learning, memory) [57,58].	**Nutritional Interventions:** Diets rich in long-chain omega-3 (DHA/EPA), berries, green tea, and other polyphenol sources upregulate BDNF. Caloric restriction and intermittent fasting can amplify neurotrophic signaling by promoting metabolic hormesis and mitochondrial efficiency [59].	**Exercise Modalities:** Aerobic training (moderate-to-vigorous intensity) and resistance training enhance circulating BDNF and facilitate its transport across the blood–brain barrier. High-intensity interval training (HIIT) also increases BDNF acutely. Combined exercise plus enriched diets synergistically boost BDNF expression [57].	**Evidence:** Rodent models show robust hippocampal BDNF increases correlating with enhanced long-term potentiation and spatial memory. Human RCTs demonstrate improved cognitive performance and hippocampal volume after aerobic interventions. Neuroimaging (MRI) shows increased gray matter volume in the hippocampus in older adults practicing regular exercise and healthy diets [56].
Metabolic and Mitochondrial Efficiency	**Molecular:** Exercise enhances mitochondrial biogenesis (via PGC-1α, NRF1, TFAM) and improves bioenergetics. Nutrients with antioxidant and anti-inflammatory properties reduce ROS and maintain mitochondrial integrity. Ketone bodies and certain dietary patterns (Mediterranean, plant-based) support efficient ATP generation and mitigate oxidative stress [60].	**Synaptic and Structural Impact:** Optimized mitochondrial function sustains synaptic transmission, buffering metabolic stress, and thereby stabilizing synaptic connectivity and network efficiency. Enhanced mitochondrial density supports synaptic remodeling and plasticity [61].	**Nutritional Patterns:** Mediterranean-type diets, rich in monounsaturated and polyunsaturated fats, whole grains, legumes, fruits, and vegetables, enhance metabolic flexibility and reduce systemic inflammation. Certain micronutrients (vitamin E, polyphenols) and intermittent fasting regimens promote mitochondrial health and neuroplasticity [62].	**Exercise Types:** Regular aerobic exercise increases hippocampal and cortical mitochondrial density and capacity for oxidative phosphorylation. Resistance training improves insulin sensitivity and reduces metabolic dysfunction, indirectly supporting neuronal energetics [63].	**Evidence:** Animal studies link improved mitochondrial function with robust synaptic resilience and delayed neurodegenerative processes. Human imaging studies (fMRI, MRS) suggest enhanced cerebral blood flow and metabolic efficiency in exercising individuals, correlating with better cognitive performance [64].
Epigenetic Regulation (DNA Methylation, Histone Modifications, miRNAs)	**Molecular:** Dietary polyphenols (resveratrol, curcumin), B vitamins, and exercise modulate epigenetic regulators such as SIRT1, HDACs, DNMTs, and miRNAs. Exercise-induced changes in histone acetylation and DNA methylation patterns favor genes involved in synaptic plasticity (BDNF, synapsin I, CREB) [65].	**Synaptic Plasticity:** Epigenetically driven gene expression shifts promote synaptogenesis, dendritic remodeling, and long-term stabilization of synaptic connections. Enhances adaptive responses to cognitive challenges and stressors [66].	**Nutritional Factors:** Nutraceuticals and diets rich in polyphenols, folate, and choline influence DNA methylation and histone modification, bolstering the transcription of plasticity-related genes. Omega-3s also modulate miRNA profiles linked to neural plasticity [67].	**Exercise Parameters:** Chronic aerobic exercise modifies epigenetic markers that regulate the expression of neurotrophic and synaptic genes. Resistance training may alter histone acetylation patterns, further enhancing neuron structural integrity [68].	**Evidence:** Animal models show that exercise-induced epigenetic modifications improve memory persistence. Human studies have associated physical activity with beneficial epigenetic signatures (reduced hypermethylation of BDNF promoter) [69].
Adult Hippocampal Neurogenesis	**Molecular/Cellular:** Exercise increases levels of VEGF, IGF-1, and BDNF, stimulating proliferation and differentiation of neural progenitor cells in the dentate gyrus. Nutrients like flavonoids and omega-3s enhance survival and maturation of newborn neurons [70].	**Structural Impact:** Enhanced neurogenesis leads to greater hippocampal volume, improved pattern separation, spatial navigation, and memory consolidation. Increases synaptic integration of new neurons into functional circuits [71].	**Dietary Enhancers:** Diets rich in cocoa flavanols, green tea catechins, blueberries, and DHA have been linked to increased neurogenesis. Ketogenic diets and intermittent fasting strategies may also support regenerative capacity by modulating insulin and growth factor pathways [72].	**Exercise Interventions:** Sustained aerobic exercise (running, cycling) robustly enhances neurogenesis. HIIT protocols can also boost neurogenesis, though more research is needed. Combined nutritional and exercise interventions can synergistically amplify hippocampal neurogenesis [73].	**Evidence:** Rodent studies consistently show increased dentate gyrus neurogenesis after exercise and flavonoid supplementation. Human imaging (MRI) has correlated higher fitness with increased hippocampal volume and memory performance in older adults [74].
Neurotransmitter System Modulation	**Molecular:** Exercise and balanced nutrition modulate dopaminergic, serotonergic, glutamatergic, and GABAergic systems. Adequate iron, B vitamins, and amino acids are essential for optimal neurotransmitter synthesis. Exercise may increase serotonin availability and receptor sensitivity, and modulate dopamine release in reward circuits [75].	**Synaptic Balancing:** Improved neurotransmitter homeostasis refines excitatory–inhibitory balance, enhancing signal-to-noise ratio in neuronal processing. This leads to better mood regulation, attention, and executive control [76].	**Nutrient Contributions:** Iron, folate, vitamin B12, and amino acids (tryptophan, tyrosine) support neurotransmitter synthesis and receptor function. Polyphenol-rich diets can modulate GABA and glutamate signaling, improving cognitive flexibility and emotional stability [77].	**Exercise Modalities:** Aerobic and resistance training can elevate serotonin and dopamine levels, improve receptor sensitivity, and modulate hippocampal and cortical glutamatergic signaling. The frequency and intensity of exercise influence the magnitude of these effects [64].	**Evidence:** Preclinical models reveal exercise-induced increases in dopamine turnover and serotonin release. Clinical studies link regular physical activity and quality diets to reduced depressive symptoms and enhanced cognitive–emotional integration [78].
Inflammation and Oxidative Stress Reduction	**Molecular:** Physical exercise reduces proinflammatory cytokines (TNF-α, IL-6) and enhances antioxidant enzyme activities (SOD, CAT). Nutrients rich in antioxidants (vitamin C, E, polyphenols) and anti-inflammatory compounds (curcumin, n-3 fatty acids) lower neuroinflammation and oxidative stress, preserving neuronal integrity [75].	**Structural Stability:** Reduced inflammation and oxidative damage maintain synaptic integrity, prevent neuronal atrophy, and support dendritic complexity. This stable environment fosters robust adaptive plasticity and decreases vulnerability to neurodegeneration [76].	**Anti-inflammatory Diets:** Mediterranean, DASH, and Nordic diets, as well as specific compounds (alpha-lipoic acid, sulforaphane), attenuate systemic and neuroinflammation. Lower systemic inflammation correlates with improved cognitive trajectories and reduced risk of dementia [77].	**Exercise Approach:** Moderate, regular aerobic exercise exerts anti-inflammatory and antioxidative effects, while high-intensity exercise might transiently increase ROS but subsequently enhance endogenous antioxidant defenses. The long-term net effect is neuroprotective [75].	**Evidence:** Animal studies show combined diet–exercise protocols reduce microglial activation and oxidative damage. Clinical trials link exercise plus dietary intervention with lowered inflammatory biomarkers and improved executive functions [77].
Hormonal and Peripheral Factor Regulation	**Molecular:** Exercise-induced release of irisin, adiponectin, and decreased cortisol levels reshape the metabolic and neurotrophic environment. Nutritional interventions maintaining glycemic control and insulin sensitivity support optimal hormone signaling to the brain [57].	**Synaptic and Network Effects:** Balanced hormonal signaling influences synaptic plasticity indirectly by regulating energy availability, neurotrophin circulation, and inflammatory status, thereby optimizing conditions for neuroplastic adaptation [79].	**Diet–Hormone Interplay:** Balanced diets stabilizing insulin and leptin signaling prevent metabolic stress on neurons. Protein-rich diets and adequate micronutrients ensure proper hormone synthesis and receptor functionality [57].	**Exercise Synergy:** Aerobic and resistance training improve peripheral insulin sensitivity, enhance irisin and adiponectin levels, and reduce cortisol, collectively promoting a more neurotrophic and less catabolic environment [79].	**Evidence:** Animal models demonstrate that exercise-driven irisin increases BDNF expression and supports neuroplastic changes. Human interventions show exercise and diet synergy improves metabolic profiles and correlates with better cognitive outcomes [79].
Functional Connectivity and Network Integration	**Network-Level:** Exercise and nutrient-rich diets improve functional connectivity within key neural networks (default mode, frontoparietal, hippocampo-cortical circuits). Enhanced vascularization (via VEGF and nitric oxide) supports network-level efficiency [80].	**Structural Connectivity:** Strengthening of white matter integrity and synaptic pruning leads to more efficient neural communication. Enhanced cerebrovascular perfusion supports neurovascular coupling and network resilience [3].	**Nutritional Quality:** Adherence to the Mediterranean diet, or diets with high polyphenolic and healthy fat content, is associated with reduced brain atrophy and improved functional connectivity, delaying cognitive decline [80].	**Exercise Integration:** Endurance training, HIIT, and combined aerobic–resistance regimens are linked with improved white matter integrity, resting-state functional connectivity, and reduced brain atrophy in aging populations [3].	**Evidence:** Neuroimaging studies (fMRI, DTI) in humans reveal that physically fit individuals with high diet quality display increased hippocampal volume, stronger connectivity in cognition-related networks, and slower age-related cognitive decline [3,80].
Gut-Brain Axis and Metabolites (Novel Aspect)	**Molecular/Systems:** The gut microbiota, modulated by diet and exercise, produces short-chain fatty acids (SCFAs), vitamins, and neurotransmitter precursors influencing brain function. Exercise alters gut microbiome composition, increasing beneficial bacteria that produce neuroprotective metabolites [81].	**Synaptic/Structural:** Improved gut barrier integrity and SCFA availability support synaptic plasticity, reduce neuroinflammation, and enhance BDNF expression. This fosters a more adaptive and resilient neural circuitry [82].	**Dietary Influence:** Fiber-rich, plant-based diets and fermented foods promote healthy microbiota that supports neural health. Polyphenols and probiotics can modulate microbial communities, influencing neurotransmitter metabolism and synaptic plasticity [81].	**Exercise Interactions:** Endurance training modulates gut flora towards a more anti-inflammatory profile, synergizing with diet to bolster neuroplasticity. Preclinical studies show exercise-induced increases in Lactobacillus and Bifidobacterium linked to improved cognition [82].	**Evidence:** Animal research links exercise- and diet-induced microbiome shifts with improved learning and stress resilience. Human studies (observational and intervention-based) correlate better gut flora diversity with enhanced cognitive performance and lower risk of neurological disorders [81,82,83].

**Table 2:** Effects and exercise modality.

Exercise Modality	Stimulus Characteristics	Molecular Pathways (Myokines, Neurotrophins)	Cognitive and Neural Effects
**Aerobic (Endurance) Training**	Continuous moderate-to-vigorous-intensity, extended-duration sessions (≥30 min)	Robust elevations in BDNF and irisin; enhanced mitochondrial function and blood flow; improved inflammatory profile	Increases hippocampal neurogenesis, improves memory, executive function, and slows age-related cognitive decline
**High, Intensity Interval Training (HIIT)**	Short, intense bouts (30 s–4 min) interspersed with low-intensity recovery	Acute, sometimes pronounced spikes in BDNF; transient metabolic stress; potential rapid induction of beneficial inflammatory and metabolic mediators	May yield immediate improvements in attention, processing speed; long-term effects on sustained cognitive function still under investigation
**Resistance Training**	Repetitive, high-load, low-to-moderate repetition schemes; focuses on muscular strength and hypertrophy	Potential moderate BDNF increases; improved insulin sensitivity, hormonal balance, and anti-inflammatory environment indirectly support neurotrophic signaling	Potential protective effect on cognitive aging; may enhance executive functions over time and support brain volume maintenance, especially in older adults
**Mind, Body Exercises**	Low-to-moderate intensity, emphasis on breathing, balance, proprioception, and mindfulness	May modulate stress hormones, inflammatory markers, and possibly support balanced myokine and neurotrophin levels, though evidence at the molecular level is less extensive	Associated with improved attention, emotional regulation, and memory consolidation; may indirectly foster a neuroprotective environment
**Combined/Multimodal Training**	Integrates multiple stimulus types (endurance + strength) within a weekly program	Potential additive effects on BDNF and irisin; improved metabolic and vascular profiles; enhanced overall systemic homeostasis	May yield comprehensive cognitive gains (memory, executive function, processing speed), improved resilience against neurodegeneration, and synergistic brain benefits

## References

[1] Jia R.X., Liang J.H., Xu Y., Wang Y.Q. (2019). Effects of Physical Activity and Exercise on the Cognitive Function of Patients with Alzheimer Disease: A Meta-Analysis. BMC Geriatr..

[2] Mahalakshmi B., Maurya N., Lee S.D., Kumar V.B. (2020). Possible Neuroprotective Mechanisms of Physical Exercise in Neurodegeneration. Int. J. Mol. Sci..

[3] Hillman C.H., Erickson K.I., Kramer A.F. (2008). Be Smart, Exercise Your Heart: Exercise Effects on Brain and Cognition. Nat. Rev. Neurosci..

[4] Phillips C. (2017). Lifestyle Modulators of Neuroplasticity: How Physical Activity, Mental Engagement, and Diet Promote Cognitive Health during Aging. Neural Plast..

[5] Schuch F.B., Vancampfort D., Firth J., Rosenbaum S., Ward P.B., Silva E.S., Hallgren M., De Leon A.P., Dunn A.L., Deslandes A.C. (2018). Physical Activity and Incident Depression: A Meta-Analysis of Prospective Cohort Studies. Am. J. Psychiatry.

[6] Gómez-Pinilla F. (2008). Brain Foods: The Effects of Nutrients on Brain Function. Nat. Rev. Neurosci..

[7] Hötting K., Röder B. (2013). Beneficial Effects of Physical Exercise on Neuroplasticity and Cognition. Neurosci. Biobehav. Rev..

[8] Gomez-Pinilla F., Hillman C. (2013). The Influence of Exercise on Cognitive Abilities. Compr. Physiol..

[9] Kim J., Campbell A.S., de Ávila B.E.F., Wang J. (2019). Wearable Biosensors for Healthcare Monitoring. Nat. Biotechnol..

[10] Bandodkar A.J., Wang J. (2014). Non-Invasive Wearable Electrochemical Sensors: A Review. Trends Biotechnol..

[11] Heikenfeld J., Jajack A., Rogers J., Gutruf P., Tian L., Pan T., Li R., Khine M., Kim J., Wang J. (2018). Wearable Sensors: Modalities, Challenges, and Prospects. Lab Chip.

[12] Natarajan A., Su H.W., Heneghan C. (2020). Assessment of Physiological Signs Associated with COVID-19 Measured Using Wearable Devices. NPJ Digit. Med..

[13] Clemente-Suárez V.J., Beltrán-Velasco A.I., Redondo-Flórez L., Martín-Rodríguez A., Tornero-Aguilera J.F. (2023). Global Impacts of Western Diet and Its Effects on Metabolism and Health: A Narrative Review. Nutrients.

[14] Clemente-Suárez V.J., Beltrán-Velasco A.I., Redondo-Flórez L., Martín-Rodríguez A., Yáñez-Sepúlveda R., Tornero-Aguilera J.F. (2023). Neuro-Vulnerability in Energy Metabolism Regulation: A Comprehensive Narrative Review. Nutrients.

[15] Martín-Rodríguez A., Bustamante-Sánchez Á., Martínez-Guardado I., Navarro-Jiménez E., Plata-SanJuan E., Tornero-Aguilera J.F., Clemente-Suárez V.J. (2022). Infancy Dietary Patterns, Development, and Health: An Extensive Narrative Review. Children.

[16] Clemente-Suárez V.J., Bustamante-Sanchez Á., Tornero-Aguilera J.F., Ruisoto P., Mielgo-Ayuso J. (2022). Inflammation in COVID-19 and the Effects of Non-Pharmacological Interventions during the Pandemic: A Review. Int. J. Mol. Sci..

[17] Clemente-Suárez V.J., Martínez-González M.B., Benitez-Agudelo J.C., Navarro-Jiménez E., Beltran-Velasco A.I., Ruisoto P., Diaz Arroyo E., Laborde-Cárdenas C.C., Tornero-Aguilera J.F. (2021). The Impact of the COVID-19 Pandemic on Mental Disorders. A Critical Review. Int. J. Environ. Res. Public Health.

[18] Bayliak M.M., Gospodaryov D.V., Lushchak V.I. (2023). Homeostasis of Carbohydrates and Reactive Oxygen Species Is Critically Changed in the Brain of Middle-Aged Mice: Molecular Mechanisms and Functional Reasons. BBA Adv..

[19] Puente-González A.S., Sánchez-González F., Hernández-Xumet J.E., Sánchez-Sánchez M.C., Barbero-Iglesias F.J., Méndez-Sánchez R. (2020). Short and Medium-Term Effects of a Multicomponent Physical Exercise Program with a Mediterranean Diet on Bone Mineral Density, Gait, Balance, and Fall Risk for Patients with Alzheimer Disease: Randomized Controlled Clinical Trial Study Protocol. Medicine.

[20] Morris M.C., Tangney C.C., Wang Y., Sacks F.M., Bennett D.A., Aggarwal N.T. (2015). MIND Diet Associated with Reduced Incidence of Alzheimer’s Disease. Alzheimer’s Dement..

[21] Wei B.Z., Li L., Dong C.W., Tan C.C., Xu W. (2023). The Relationship of Omega-3 Fatty Acids with Dementia and Cognitive Decline: Evidence from Prospective Cohort Studies of Supplementation, Dietary Intake, and Blood Markers. Am. J. Clin. Nutr..

[22] Ellouze I., Sheffler J., Nagpal R., Arjmandi B. (2023). Dietary Patterns and Alzheimer’s Disease: An Updated Review Linking Nutrition to Neuroscience. Nutrients.

[23] Kang J., Park M., Lee E., Jung J., Kim T. (2022). The Role of Vitamin D in Alzheimer’s Disease: A Transcriptional Regulator of Amyloidopathy and Gliopathy. Biomedicines.

[24] Harding S.L., Bishop J. (2022). The Gut Microbiome, Mental Health, and Cognitive and Neurodevelopmental Disorders: A Scoping Review. J. Nurse Pr..

[25] Jin J., Jin Q., Wang X., Akoh C.C. (2020). High Sn-2 Docosahexaenoic Acid Lipids for Brain Benefits, and Their Enzymatic Syntheses: A Review. Engineering.

[26] Derbyshire E. (2018). Brain Health across the Lifespan: A Systematic Review on the Role of Omega-3 Fatty Acid Supplements. Nutrients.

[27] Scheinman S.B., Sugasini D., Zayed M., Yalagala P.C.R., Marottoli F.M., Subbaiah P.V., Tai L.M. (2021). LPC-DHA/EPA-Enriched Diets Increase Brain DHA and Modulate Behavior in Mice That Express Human APOE4. Front. Neurosci..

[28] Kocyigit A., Selek S. (2016). Exogenous Antioxidants Are Double-Edged Swords. Bezmialem Sci..

[29] Wang J., Song Y., Gao M., Bai X., Chen Z. (2016). Neuroprotective Effect of Several Phytochemicals and Its Potential Application in the Prevention of Neurodegenerative Diseases. Geriatrics.

[30] McGurran H., Glenn J., Madero E., Bott N. (2020). Risk Reduction and Prevention of Alzheimer’s Disease: Biological Mechanisms of Diet. Curr. Alzheimer Res..

[31] Tofighi N., Asle-Rousta M., Rahnema M., Amini R. (2021). The Anxiolytic Effect of Alpha-Linoleic Acid in Alzheimer’s Disease Model Rats Is Mediated by Enhanced Brain-Derived Neurotrophic Factor Expression. J. Knowl. Health Basic Med. Sci..

[32] Wysoczański T., Sokoła-Wysoczańska E., Pękala J., Lochyński S., Czyż K., Bodkowski R., Herbinger G., Patkowska-Sokoła B., Librowski T. (2016). Omega-3 Fatty Acids and Their Role in Central Nervous System—A Review. Curr. Med. Chem..

[33] Regner-Nelke L., Nelke C., Schroeter C.B., Dziewas R., Warnecke T., Ruck T., Meuth S.G. (2021). Enjoy Carefully: The Multifaceted Role of Vitamin e in Neuro-Nutrition. Int. J. Mol. Sci..

[34] Kangisser L., Tan E., Bellomo R., Deane A.M., Plummer M.P. (2021). Neuroprotective Properties of Vitamin C: A Scoping Review of Pre-Clinical and Clinical Studies. J. Neurotrauma.

[35] Tucker K.L., Qiao N., Scott T., Rosenberg I., Spiro A. (2005). High Homocysteine and Low B Vitamins Predict Cognitive Decline in Aging Men: The Veterans Affairs Normative Aging Study. Am. J. Clin. Nutr..

[36] McNulty H., Ward M., Hoey L., Hughes C.F., Pentieva K. (2019). Addressing Optimal Folate and Related B-Vitamin Status through the Lifecycle: Health Impacts and Challenges. Proc. Nutr. Soc..

[37] García R.M.M., Ortega A.I.J., López-Sobaler A.M., Ortega R.M. (2018). Nutrition Strategies That Improve Cognitive Function. Nutr. Hosp..

[38] Abrego-Guandique D.M., Bonet M.L., Caroleo M.C., Cannataro R., Tucci P., Ribot J., Cione E. (2023). The Effect of Beta-Carotene on Cognitive Function: A Systematic Review. Brain Sci..

[39] Lauer A.A., Grimm H.S., Apel B., Golobrodska N., Kruse L., Ratanski E., Schulten N., Schwarze L., Slawik T., Sperlich S. (2022). Mechanistic Link between Vitamin B12 and Alzheimer’s Disease. Biomolecules.

[40] Li S., Guo Y., Men J., Fu H., Xu T. (2021). The Preventive Efficacy of Vitamin B Supplements on the Cognitive Decline of Elderly Adults: A Systematic Review and Meta-Analysis. BMC Geriatr..

[41] Maurya V.K., Shakya A., McClements D.J., Srinivasan R., Bashir K., Ramesh T., Lee J., Sathiyamoorthi E. (2023). Vitamin C Fortification: Need and Recent Trends in Encapsulation Technologies. Front. Nutr..

[42] Bournival J., Quessy P., Martinoli M.G. (2009). Protective Effects of Resveratrol and Quercetin against MPP+ -Induced Oxidative Stress Act by Modulating Markers of Apoptotic Death in Dopaminergic Neurons. Cell. Mol. Neurobiol..

[43] Gardener S.L., Rainey-Smith S.R., Weinborn M., Bondonno C.P., Martins R.N. (2021). Intake of Products Containing Anthocyanins, Flavanols, and Flavanones, and Cognitive Function: A Narrative Review. Front. Aging Neurosci..

[44] Rezai-Zadeh K., Arendash G.W., Hou H., Fernandez F., Jensen M., Runfeldt M., Shytle R.D., Tan J. (2008). Green Tea Epigallocatechin-3-Gallate (EGCG) Reduces β-Amyloid Mediated Cognitive Impairment and Modulates Tau Pathology in Alzheimer Transgenic Mice. Brain Res..

[45] Jenkins T., Nguyen J., Polglaze K., Bertrand P. (2016). Influence of Tryptophan and Serotonin on Mood and Cognition with a Possible Role of the Gut-Brain Axis. Nutrients.

[46] Meeusen R., Decroix L. (2018). Nutritional Supplements and the Brain. Int. J. Sport Nutr. Exerc. Metab..

[47] Young S.N. (2024). How to Increase Serotonin in the Human Brain without Drugs. J. Psychiatry Neurosci..

[48] Freir D.B., Fedriani R., Scully D., Smith I.M., Selkoe D.J., Walsh D.M., Regan C.M. (2011). Aβ Oligomers Inhibit Synapse Remodelling Necessary for Memory Consolidation. Neurobiol. Aging.

[49] Clark A., Mach N. (2016). Exercise-Induced Stress Behavior, Gut-Microbiota-Brain Axis and Diet: A Systematic Review for Athletes. J. Int. Soc. Sports Nutr..

[50] Jonasson L.S., Nyberg L., Kramer A.F., Lundquist A., Riklund K., Boraxbekk C.J. (2017). Aerobic Exercise Intervention, Cognitive Performance, and Brain Structure: Results from the Physical Influences on Brain in Aging (PHIBRA) Study. Front. Aging Neurosci..

[51] Su H.M. (2010). Mechanisms of N-3 Fatty Acid-Mediated Development and Maintenance of Learning Memory Performance. J. Nutr. Biochem..

[52] Zerbes G., Kausche F.M., Schwabe L. (2020). Stress-Induced Cortisol Modulates the Control of Memory Retrieval towards the Dorsal Striatum. Eur. J. Neurosci..

[53] Zhao C., Noble J.M., Marder K., Hartman J.S., Gu Y., Scarmeas N. (2018). Dietary Patterns, Physical Activity, Sleep, and Risk for Dementia and Cognitive Decline. Curr. Nutr. Rep..

[54] Li Q., Gong B., Zhao Y., Wu C. (2023). Effect of Exercise Cognitive Combined Training on Physical Function in Cognitively Healthy Older Adults: A Systematic Review and Meta-Analysis. J. Aging Phys. Act..

[55] Peng J., Yuan Y., Zhao Y., Ren H. (2022). Effects of Exercise on Patients with Obstructive Sleep Apnea: A Systematic Review and Meta-Analysis. Int. J. Environ. Res. Public Health.

[56] Erickson K.I., Voss M.W., Prakash R.S., Basak C., Szabo A., Chaddock L., Kim J.S., Heo S., Alves H., White S.M. (2011). Exercise Training Increases Size of Hippocampus and Improves Memory. Proc. Natl. Acad. Sci. USA.

[57] Wrann C.D., White J.P., Salogiannnis J., Laznik-Bogoslavski D., Wu J., Ma D., Lin J.D., Greenberg M.E., Spiegelman B.M. (2013). Exercise Induces Hippocampal BDNF through a PGC-1α/FNDC5 Pathway. Cell Metab..

[58] Liu P.Z., Nusslock R. (2018). Exercise-Mediated Neurogenesis in the Hippocampus via BDNF. Front. Neurosci..

[59] Stillman C.M., Cohen J., Lehman M.E., Erickson K.I. (2016). Mediators of Physical Activity on Neurocognitive Function: A Review at Multiple Levels of Analysis. Front. Hum. Neurosci..

[60] Mattson M.P. (2012). Energy Intake and Exercise as Determinants of Brain Health and Vulnerability to Injury and Disease. Cell Metab..

[61] Valenzuela P.L., Castillo-García A., Morales J.S., de la Villa P., Hampel H., Emanuele E., Lista S., Lucia A. (2020). Exercise Benefits on Alzheimer’s Disease: State-of-the-Science. Ageing Res. Rev..

[62] Tarumi T., Zhang R. (2015). The Role of Exercise-Induced Cardiovascular Adaptation in Brain Health. Exerc. Sport Sci. Rev..

[63] Nikolaidis M.G., Kyparos A., Spanou C., Paschalis V., Theodorou A.A., Vrabas I.S. (2012). Redox Biology of Exercise: An Integrative and Comparative Consideration of Some Overlooked Issues. J. Exp. Biol..

[64] Meeusen R. (2014). Exercise, Nutrition and the Brain. Sports Med..

[65] Griñán-Ferré C., Sarroca S., Ivanova A., Puigoriol-Illamola D., Aguado F., Camins A., Sanfeliu C., Pallàs M. (2016). Epigenetic Mechanisms Underlying Cognitive Impairment and Alzheimer Disease Hallmarks in 5XFAD Mice. Aging.

[66] Kauppinen A., Suuronen T., Ojala J., Kaarniranta K., Salminen A. (2013). Antagonistic Crosstalk between NF-ΚB and SIRT1 in the Regulation of Inflammation and Metabolic Disorders. Cell. Signal..

[67] Coker S.J., Smith-Díaz C.C., Dyson R.M., Vissers M.C.M., Berry M.J. (2022). The Epigenetic Role of Vitamin C in Neurodevelopment. Int. J. Mol. Sci..

[68] Fernandes J., Arida R.M., Gomez-Pinilla F. (2017). Physical Exercise as an Epigenetic Modulator of Brain Plasticity and Cognition. Neurosci. Biobehav. Rev..

[69] de Meireles L.C.F., Galvão F., Walker D.M., Cechinel L.R., de Souza Grefenhagen Á.I., Andrade G., Palazzo R.P., Lovatel G.A., Basso C.G., Nestler E.J. (2019). Exercise Modalities Improve Aversive Memory and Survival Rate in Aged Rats: Role of Hippocampal Epigenetic Modifications. Mol. Neurobiol..

[70] Baek S.-S. (2016). Role of Exercise on the Brain. J. Exerc. Rehabil..

[71] Neeper S.A., Góauctemez-Pinilla F., Choi J., Cotman C. (1995). Exercise and Brain Neurotrophins. Nature.

[72] Van Praag H., Kempermann G., Gage F.H. (1999). Running Increases Cell Proliferation and Neurogenesis in the Adult Mouse Dentate Gyrus. Nat. Neurosci..

[73] Okamoto M., Mizuuchi D., Omura K., Lee M., Oharazawa A., Yook J.S., Inoue K., Soya H. (2021). High-Intensity Intermittent Training Enhances Spatial Memory and Hippocampal Neurogenesis Associated with BDNF Signaling in Rats. Cerebral Cortex.

[74] Pereira A.C., Huddleston D.E., Brickman A.M., Sosunov A.A., Hen R., McKhann G.M., Sloan R., Gage F.H., Brown T.R., Small S.A. (2007). An in Vivo Correlate of Exercise-Induced Neurogenesis in the Adult Dentate Gyrus. Proc. Natl. Acad. Sci. USA.

[75] Firth J., Stubbs B., Vancampfort D., Schuch F., Lagopoulos J., Rosenbaum S., Ward P.B. (2018). Effect of Aerobic Exercise on Hippocampal Volume in Humans: A Systematic Review and Meta-Analysis. Neuroimage.

[76] Meeusen R., De Meirleir K. (1995). Exercise and Brain Neurotransmission. Sports Med..

[77] Strasser B., Gostner J.M., Fuchs D. (2016). Mood, Food, and Cognition: Role of Tryptophan and Serotonin. Curr. Opin. Clin. Nutr. Metab. Care.

[78] Xie Y., Wu Z., Sun L., Zhou L., Wang G., Xiao L., Wang H. (2021). The Effects and Mechanisms of Exercise on the Treatment of Depression. Front. Psychiatry.

[79] Foster P.P. (2015). Role of Physical and Mental Training in Brain Network Configuration. Front. Aging Neurosci..

[80] Rothman S.M., Mattson M.P. (2013). Activity-Dependent, Stress-Responsive BDNF Signaling and the Quest for Optimal Brain Health and Resilience throughout the Lifespan. Neuroscience.

[81] Cullen J.M.A., Shahzad S., Dhillon J. (2023). A Systematic Review on the Effects of Exercise on Gut Microbial Diversity, Taxonomic Composition, and Microbial Metabolites: Identifying Research Gaps and Future Directions. Front. Physiol..

[82] Beltrán-Velasco A.I., Clemente-Suárez V.J. (2025). Harnessing Gut Microbiota for Biomimetic Innovations in Health and Biotechnology. Biomimetics.

[83] Pedersen B.K., Febbraio M.A. (2012). Muscles, Exercise and Obesity: Skeletal Muscle as a Secretory Organ. Nat. Rev. Endocrinol..

[84] Clemente-Suárez V.J., Rubio-Zarapuz A., Belinchón-deMiguel P., Beltrán-Velasco A.I., Martín-Rodríguez A., Tornero-Aguilera J.F. (2024). Impact of Physical Activity on Cellular Metabolism Across Both Neurodegenerative and General Neurological Conditions: A Narrative Review. Cells.

[85] Ngandu T., Lehtisalo J., Solomon A., Levälahti E., Ahtiluoto S., Antikainen R., Bäckman L., Hänninen T., Jula A., Laatikainen T. (2015). A 2 year multidomain intervention of diet, exercise, cognitive training, and vascular risk monitoring versus control to prevent cognitive decline in at-risk elderly people (FINGER): A randomised controlled trial. Lancet Neurol..

[86] Keawtep P., Sungkarat S., Boripuntakul S., Sa-Nguanmoo P., Wichayanrat W., Chattipakorn S.C., Worakul P. (2024). Effects of combined dietary intervention and physical-cognitive exercise on cognitive function and cardiometabolic health of postmenopausal women with obesity: A randomized controlled trial. Int. J. Behav. Nutr. Phys. Act..

[87] Cermak N.M., Res P.T., De Groot L.C.P.G.M., Saris W.H.M., Van Loon L.J.C. (2012). Protein Supplementation Augments the Adaptive Response of Skeletal Muscle to Resistance-Type Exercise Training: A Meta-Analysis. Am. J. Clin. Nutr..

[88] Wang B., Liang J., Lu C., Lu A., Wang C. (2024). Exercise Regulates Myokines in Aging-Related Diseases through Muscle-Brain Crosstalk. Gerontology.

[89] Gomez-Pinilla F. (2008). The Influences of Diet and Exercise on Mental Health through Hormesis. Ageing Res. Rev..

[90] Saucedo Marquez C.M., Vanaudenaerde B., Troosters T., Wenderoth N. (2015). High-Intensity Interval Training Evokes Larger Serum BDNF Levels Compared with Intense Continuous Exercise. J. Appl. Physiol..

[91] Cotman C.W., Berchtold N.C. (2002). Exercise: A Behavioral Intervention to Enhance Brain Health and Plasticity. Trends Neurosci..

[92] Kendig M.D., Leigh S.J., Morris M.J. (2021). Unravelling the Impacts of Western-Style Diets on Brain, Gut Microbiota and Cognition. Neurosci. Biobehav. Rev..

[93] Callow D.D., Kommula Y., Stark C.E.L., Smith J.C. (2023). Acute Cycling Exercise and Hippocampal Subfield Function and Microstructure in Healthy Older Adults. Hippocampus.

[94] Gothe N.P., Khan I., Hayes J., Erlenbach E., Damoiseaux J.S. (2019). Yoga Effects on Brain Health: A Systematic Review of the Current Literature. Brain Plast..

[95] Neuvonen E., Lehtisalo J., Solomon A., Antikainen R., Havulinna S., Hänninen T., Laatikainen T., Lindström J., Rautio N., Soininen H. (2022). Psychosocial Determinants for Adherence to a Healthy Lifestyle and Intervention Participation in the FINGER Trial: An Exploratory Analysis of a Randomised Clinical Trial. Aging Clin. Exp. Res..

[96] Bäckhed F., Roswall J., Peng Y., Feng Q., Jia H., Kovatcheva-Datchary P., Li Y., Xia Y., Xie H., Zhong H. (2015). Dynamics and Stabilization of the Human Gut Microbiome during the First Year of Life. Cell Host Microbe.

[97] Hoban A.E., Moloney R.D., Golubeva A.V., McVey Neufeld K.A., O’Sullivan O., Patterson E., Stanton C., Dinan T.G., Clarke G., Cryan J.F. (2016). Behavioural and Neurochemical Consequences of Chronic Gut Microbiota Depletion during Adulthood in the Rat. Neuroscience.

[98] Tillisch K., Labus J., Kilpatrick L., Jiang Z., Stains J., Ebrat B., Guyonnet D., Legrain-Raspaud S., Trotin B., Naliboff B. (2013). Consumption of Fermented Milk Product with Probiotic Modulates Brain Activity. Gastroenterology.

[99] Shi H., Ge X., Ma X., Zheng M., Cui X., Pan W., Zheng P., Yang X., Zhang P., Hu M. (2021). A Fiber-Deprived Diet Causes Cognitive Impairment and Hippocampal Microglia-Mediated Synaptic Loss through the Gut Microbiota and Metabolites. Microbiome.

[100] Dalile B., Van Oudenhove L., Vervliet B., Verbeke K. (2019). The Role of Short-Chain Fatty Acids in Microbiota–Gut–Brain Communication. Nat. Rev. Gastroenterol. Hepatol..

[101] Moloney R.D., Dinan T.G., Cryan J.F. (2015). Stress & the Microbiota–Gut–Brain Axis in Visceral Pain. Psychoneuroendocrinology.

[102] Margolis K.G., Cryan J.F., Mayer E.A. (2021). The Microbiota-Gut-Brain Axis: From Motility to Mood. Gastroenterology.

[103] Bravo J.A., Forsythe P., Chew M.V., Escaravage E., Savignac H.M., Dinan T.G., Bienenstock J., Cryan J.F. (2011). Ingestion of Lactobacillus Strain Regulates Emotional Behavior and Central GABA Receptor Expression in a Mouse via the Vagus Nerve. Proc. Natl. Acad. Sci. USA.

[104] Vauzour D. (2012). Dietary Polyphenols as Modulators of Brain Functions: Biological Actions and Molecular Mechanisms Underpinning Their Beneficial Effects. Oxidative Med. Cell. Longev..

[105] Berding K., Vlckova K., Marx W., Schellekens H., Stanton C., Clarke G., Jacka F., Dinan T.G., Cryan J.F. (2021). Diet and the Microbiota-Gut-Brain Axis: Sowing the Seeds of Good Mental Health. Adv. Nutr. Int. Rev. J..

[106] Dumitrescu L., Popescu-Olaru I., Cozma L., Tulbǎ D., Hinescu M.E., Ceafalan L.C., Gherghiceanu M., Popescu B.O. (2018). Oxidative Stress and the Microbiota-Gut-Brain Axis. Oxid. Med. Cell. Longev..

[107] Sordillo J.E., Korrick S., Laranjo N., Carey V., Weinstock G.M., Gold D.R., O’Connor G., Sandel M., Bacharier L.B., Beigelman A. (2019). Association of the Infant Gut Microbiome With Early Childhood Neurodevelopmental Outcomes. JAMA Netw. Open.

[108] Cryan J.F., Dinan T.G. (2012). Mind-Altering Microorganisms: The Impact of the Gut Microbiota on Brain and Behaviour. Nat. Rev. Neurosci..

[109] Sampson T.R., Mazmanian S.K. (2015). Control of Brain Development, Function, and Behavior by the Microbiome. Cell Host Microbe.

[110] Akbari E., Asemi Z., Daneshvar Kakhaki R., Bahmani F., Kouchaki E., Tamtaji O.R., Hamidi G.A., Salami M. (2016). Effect of Probiotic Supplementation on Cognitive Function and Metabolic Status in Alzheimer’s Disease: A Randomized, Double-Blind and Controlled Trial. Front. Aging Neurosci..

[111] Ghosh T.S., Rampelli S., Jeffery I.B., Santoro A., Neto M., Capri M., Giampieri E., Jennings A., Candela M., Turroni S. (2020). Mediterranean Diet Intervention Alters the Gut Microbiome in Older People Reducing Frailty and Improving Health Status: The NU-AGE 1-Year Dietary Intervention across Five European Countries. Gut.

[112] Dinan T.G., Cryan J.F. (2013). Melancholic Microbes: A Link between Gut Microbiota and Depression?. Neurogastroenterol. Motil..

[113] Martínez-Lapiscina E.H., Clavero P., Toledo E., Estruch R., Salas-Salvadó J., San Julián B., Sanchez-Tainta A., Ros E., Valls-Pedret C., Martinez-Gonzalez M.Á. (2013). Mediterranean Diet Improves Cognition: The PREDIMED-NAVARRA Randomised Trial. J. Neurol. Neurosurg. Psychiatry.

[114] Magnusson K.R., Hauck L., Jeffrey B.M., Elias V., Humphrey A., Nath R., Perrone A., Bermudez L.E. (2015). Relationships between Diet-Related Changes in the Gut Microbiome and Cognitive Flexibility. Neuroscience.

[115] Halliwell B. (2006). Oxidative Stress and Neurodegeneration: Where Are We Now?. J. Neurochem..

[116] Sies H. (2017). Hydrogen Peroxide as a Central Redox Signaling Molecule in Physiological Oxidative Stress: Oxidative Eustress. Redox Biol..

[117] Butterfield D.A., Halliwell B. (2019). Oxidative Stress, Dysfunctional Glucose Metabolism and Alzheimer Disease. Nat. Rev. Neurosci..

[118] Puri S., Shaheen M., Grover B. (2023). Nutrition and Cognitive Health: A Life Course Approach. Front. Public Health.

[119] Liu Z., Zhou T., Ziegler A.C., Dimitrion P., Zuo L. (2017). Oxidative Stress in Neurodegenerative Diseases: From Molecular Mechanisms to Clinical Applications. Oxid. Med. Cell. Longev..

[120] Mattson M.P. (2005). Energy Intake, Meal Frequency, and Health: A Neurobiological Perspective. Annu. Rev. Nutr..

[121] Sena L.A., Chandel N.S. (2012). Physiological Roles of Mitochondrial Reactive Oxygen Species. Mol. Cell.

[122] Joseph J.A., Shukitt-Hale B., Lau F.C. (2007). Fruit Polyphenols and Their Effects on Neuronal Signaling and Behavior in Senescence. Ann. New York Acad. Sci..

[123] Guo C., Sun L., Chen X., Zhang D. (2013). Oxidative Stress, Mitochondrial Damage and Neurodegenerative Diseases. Neural Regen. Res..

[124] Halliwell B., Gutteridge J.M.C. (2015). Free Radicals in Biology and Medicine.

[125] Tramutola A., Lanzillotta C., Perluigi M., Butterfield D.A. (2017). Oxidative Stress, Protein Modification and Alzheimer Disease. Brain Res. Bull..

[126] Sun M., Ma K., Wen J., Wang G., Zhang C., Li Q., Bao X., Wang H. (2020). A Review of the Brain-Gut-Microbiome Axis and the Potential Role of Microbiota in Alzheimer’s Disease. J. Alzheimer’s Dis..

[127] Vauzour D., Vafeiadou K., Rodriguez-Mateos A., Rendeiro C., Spencer J.P.E. (2008). The Neuroprotective Potential of Flavonoids: A Multiplicity of Effects. Genes Nutr..

[128] Scarmeas N., Stern Y., Tang M., Mayeux R., Luchsinger J.A. (2006). Mediterranean Diet and Risk for Alzheimer’s Disease. Ann. Neurol..

[129] Sang L., Liu C., Wang L., Zhang J., Zhang Y., Li P., Qiao L., Li C., Qiu M. (2020). Disrupted Brain Structural Connectivity Network in Subcortical Ischemic Vascular Cognitive Impairment With No Dementia. Front. Aging Neurosci..

[130] Radak Z., Hart N., Sarga L., Koltai E., Atalay M., Ohno H., Boldogh I. (2010). Exercise Plays a Preventive Role Against Alzheimer’s Disease. J. Alzheimer’s Dis..

[131] Phillips M.C.L. (2019). Fasting as a Therapy in Neurological Disease. Nutrients.

[132] Dias V., Junn E., Mouradian M.M. (2013). The Role of Oxidative Stress in Parkinson’s Disease. J. Park. Dis..

[133] Sekikawa A., Cui C., Sugiyama D., Fabio A., Harris W.S., Zhang X. (2019). Effect of High-Dose Marine Omega-3 Fatty Acids on Atherosclerosis: A Systematic Review and Meta-Analysis of Randomized Clinical Trials. Nutrients.

[134] Calis Z., Mogulkoc R., Baltaci A.K. (2019). The Roles of Flavonols/Flavonoids in Neurodegeneration and Neuroinflammation. Mini Rev. Med. Chem..

[135] Cory H., Passarelli S., Szeto J., Tamez M., Mattei J. (2018). The Role of Polyphenols in Human Health and Food Systems: A Mini-Review. Front. Nutr..

[136] Murawska-Ciałowicz E., Wiatr M., Ciałowicz M., de Assis G.G., Borowicz W., Rocha-Rodrigues S., Paprocka-Borowicz M., Marques A. (2021). Bdnf Impact on Biological Markers of Depression—Role of Physical Exercise and Training. Int. J. Environ. Res. Public Health.

[137] Müller P., Duderstadt Y., Lessmann V., Müller N.G. (2020). Lactate and BDNF: Key Mediators of Exercise Induced Neuroplasticity?. J. Clin. Med..

[138] Xing Y., Bai Y. (2020). A Review of Exercise-Induced Neuroplasticity in Ischemic Stroke: Pathology and Mechanisms. Mol. Neurobiol..

[139] von Bohlen und Halbach O. (2022). Editorial: Cellular and Molecular Responses to Changes in Nutrition and Exercise. Front. Cell. Neurosci..

[140] Nct (2015). Neural Changes of Exercise: A Functional MRI Study. https://clinicaltrials.gov/show/NCT02541136.

[141] Pickersgill J.W., Turco C.V., Ramdeo K., Rehsi R.S., Foglia S.D., Nelson A.J. (2022). The Combined Influences of Exercise, Diet and Sleep on Neuroplasticity. Front. Psychol..

[142] Lin T.-W., Tsai S.-F., Kuo Y.-M. (2018). Physical Exercise Enhances Neuroplasticity and Delays Alzheimer’s Disease. Brain Plast..

[143] Clemente-Suárez V.J., Redondo-Flórez L., Beltrán-Velasco A.I., Belinchón-deMiguel P., Ramos-Campo D.J., Curiel-Regueros A., Martín-Rodríguez A., Tornero-Aguilera J.F. (2024). The Interplay of Sports and Nutrition in Neurological Health and Recovery. J. Clin. Med..

[144] Schmolesky M.T., Webb D.L., Hansen R.A. (2013). The Effects of Aerobic Exercise Intensity and Duration on Levels of Brain- Derived Neurotrophic Factor in Healthy Men. J. Sports Sci. Med..

[145] Hao Z., Zhang X., Wang Y. (2024). Evidence of the Long-Term Protective Effect of Moderate-Intensity Physical Activity on Cognitive Function in Middle-Aged and Elderly Individuals: A Predictive Analysis of Longitudinal Studies. Life.

[146] Wu Z., Zhang H., Miao X., Li H., Pan H., Zhou D., Liu Y., Li Z., Wang J., Liu X. (2021). High-Intensity Physical Activity Is Not Associated with Better Cognition in the Elder: Evidence from the China Health and Retirement Longitudinal Study. Alzheimer’s Res. Ther..

[147] Levinger I., Goodman C., Matthews V., Hare D.L., Jerums G., Garnham A., Selig S. (2008). BDNF, Metabolic Risk Factors, and Resistance Training in Middle-Aged Individuals. Med. Sci. Sports Exerc..

[148] De Sousa Fernandes M.S., Ordônio T.F., Santos G.C.J., Santos L.E.R., Calazans C.T., Gomes D.A., Santos T.M. (2020). Effects of Physical Exercise on Neuroplasticity and Brain Function: A Systematic Review in Human and Animal Studies. Neural Plast..

[149] Baek J.-E., Hyeon S.-J., Kim M., Cho H., Hahm S.-C. (2024). Effects of Dual-Task Resistance Exercise on Cognition, Mood, Depression, Functional Fitness, and Activities of Daily Living in Older Adults with Cognitive Impairment: A Single-Blinded, Randomized Controlled Trial. BMC Geriatr..

[150] Jackson P.A., Pialoux V., Corbett D., Drogos L., Erickson K.I., Eskes G.A., Poulin M.J. (2016). Promoting Brain Health through Exercise and Diet in Older Adults: A Physiological Perspective. J. Physiol..

[151] Ogoh S., Ainslie P.N. (2009). Cerebral Blood Flow during Exercise: Mechanisms of Regulation. J. Appl. Physiol..

[152] Olivo G., Nilsson J., Garzón B., Lebedev A., Wåhlin A., Tarassova O., Ekblom M., Lövdén M. (2021). Immediate Effects of a Single Session of Physical Exercise on Cognition and Cerebral Blood Flow: A Randomized Controlled Study of Older Adults. NeuroImage.

[153] Melgar-Locatelli S., de Ceglia M., Mañas-Padilla M.C., Rodriguez-Pérez C., Castilla-Ortega E., Castro-Zavala A., Rivera P. (2023). Nutrition and Adult Neurogenesis in the Hippocampus: Does What You Eat Help You Remember?. Front. Neurosci..

[154] Davinelli S., Medoro A., Ali S., Passarella D., Intrieri M., Scapagnini G. (2022). Dietary Flavonoids and Adult Neurogenesis: Potential Implications for Brain Aging. Curr. Neuropharmacol..

[155] Anbari-Nogyni Z., Bidaki R., Madadizadeh F., Sangsefidi Z.S., Fallahzadeh H., Karimi-Nazari E., Nadjarzadeh A. (2020). Relationship of Zinc Status with Depression and Anxiety among Elderly Population. Clin. Nutr. ESPEN.

[156] Asigbee F.M., Whitney S.D., Peterson C.E. (2018). The Link Between Nutrition and Physical Activity in Increasing Academic Achievement. J. Sch. Health.

[157] Antonopoulou M., Mantzorou M., Serdari A., Bonotis K., Vasios G., Pavlidou E., Trifonos C., Vadikolias K., Petridis D., Giaginis C. (2020). Evaluating Mediterranean Diet Adherence in University Student Populations: Does This Dietary Pattern Affect Students’ Academic Performance and Mental Health?. Int. J. Heal. Plan. Manag..

[158] Aune D. (2019). Plant Foods, Antioxidant Biomarkers, and the Risk of Cardiovascular Disease, Cancer, and Mortality: A Review of the Evidence. Adv. Nutr. Int. Rev. J..

[159] Craig W.J., Mangels A.R., Fresán U., Marsh K., Miles F.L., Saunders A.V., Haddad E.H., Heskey C.E., Johnston P., Larson-Meyer E. (2021). The Safe and Effective Use of Plant-Based Diets with Guidelines for Health Professionals. Nutrients.

[160] Nehlig A. (2013). The Neuroprotective Effects of Cocoa Flavanol and Its Influence on Cognitive Performance. Br. J. Clin. Pharmacol..

[161] Arts I.C.W., Hollman P.C.H. (2005). Polyphenols and Disease Risk in Epidemiologic Studies. Am. J. Clin. Nutr..

[162] Cho J.A., Park E. (2015). Curcumin Utilizes the Anti-Inflammatory Response Pathway to Protect the Intestine against Bacterial Invasion. Nutr. Res. Pract..

[163] Sun G.Y., Simonyi A., Fritsche K.L., Chuang D.Y., Hannink M., Gu Z., Greenlief C.M., Yao J.K., Lee J.C., Beversdorf D.Q. (2018). Docosahexaenoic Acid (DHA): An Essential Nutrient and a Nutraceutical for Brain Health and Diseases. Prostaglandins Leukot. Essent. Fatty Acids.

[164] Sambra V., Echeverria F., Valenzuela A., Chouinard-Watkins R., Valenzuela R. (2021). Docosahexaenoic and Arachidonic Acids as Neuroprotective Nutrients throughout the Life Cycle. Nutrients.

[165] Roberts S.B., Franceschini M.A., Silver R.E., Taylor S.F., De Sa A.B., Có R., Sonco A., Krauss A., Taetzsch A., Webb P. (2020). Effects of Food Supplementation on Cognitive Function, Cerebral Blood Flow, and Nutritional Status in Young Children at Risk of Undernutrition: Randomized Controlled Trial. BMJ.

[166] Martínez V.G., Salas A.A., Ballestín S.S. (2022). Vitamin Supplementation and Dementia: A Systematic Review. Nutrients.

[167] McCaddon A. (2013). Vitamin B12 in Neurology and Ageing; Clinical and Genetic Aspects. Biochimie.

[168] McGeown J.P., Hume P.A., Theadom A., Quarrie K.L., Borotkanics R. (2021). Nutritional Interventions to Improve Neurophysiological Impairments Following Traumatic Brain Injury: A Systematic Review. J. Neurosci. Res..

[169] Muscaritoli M. (2021). The Impact of Nutrients on Mental Health and Well-Being: Insights From the Literature. Front. Nutr..

[170] Hutton C.P., Déry N., Rosa E., Lemon J.A., Rollo C.D., Boreham D.R., Fahnestock M., de Catanzaro D., Wojtowicz J.M., Becker S. (2015). Synergistic Effects of Diet and Exercise on Hippocampal Function in Chronically Stressed Mice. Neuroscience.

[171] Aguiló A., Tauler P., Sureda A., Cases N., Tur J., Pons A. (2007). Antioxidant Diet Supplementation Enhances Aerobic Performance in Amateur Sportsmen. J. Sports Sci..

[172] Mabrey G., Koozehchian M.S., Newton A.T., Naderi A., Forbes S.C., Haddad M. (2024). The Effect of Creatine Nitrate and Caffeine Individually or Combined on Exercise Performance and Cognitive Function: A Randomized, Crossover, Double-Blind, Placebo-Controlled Trial. Nutrients.

[173] Wang J., Rang Y., Liu C. (2024). Effects of Caloric Restriction and Intermittent Fasting and Their Combined Exercise on Cognitive Functioning: A Review. Curr. Nutr. Rep..

[174] Dominguez L.J., Veronese N., Vernuccio L., Catanese G., Inzerillo F., Salemi G., Barbagallo M. (2021). Nutrition, Physical Activity, and Other Lifestyle Factors in the Prevention of Cognitive Decline and Dementia. Nutrients.

[175] Pastor R., Tur J.A. (2020). Response to Exercise in Older Adults Who Take Supplements of Antioxidants and/or Omega-3 Polyunsaturated Fatty Acids: A Systematic Review. Biochem. Pharmacol..

[176] Köbe T., Witte A.V., Schnelle A., Lesemann A., Fabian S., Tesky V.A., Pantel J., Flöel A. (2016). Combined Omega-3 Fatty Acids, Aerobic Exercise and Cognitive Stimulation Prevents Decline in Gray Matter Volume of the Frontal, Parietal and Cingulate Cortex in Patients with Mild Cognitive Impairment. Neuroimage.

[177] Bischoff-Ferrari H.A., Vellas B., Rizzoli R., Kressig R.W., Da Silva J.A.P., Blauth M., Felson D.T., McCloskey E.V., Watzl B., Hofbauer L.C. (2020). Effect of Vitamin D Supplementation, Omega-3 Fatty Acid Supplementation, or a Strength-Training Exercise Program on Clinical Outcomes in Older Adults: The DO-HEALTH Randomized Clinical Trial. JAMA J. Am. Med. Assoc..

[178] Mundell N.L., Owen P.J., Dalla Via J., MacPherson H., Daly R., Livingston P.M., Rantalainen T., Foulkes S., Millar J., Murphy D.G. (2022). Effects of a Multicomponent Resistance-Based Exercise Program with Protein, Vitamin D and Calcium Supplementation on Cognition in Men with Prostate Cancer Treated with ADT: Secondary Analysis of a 12-Month Randomised Controlled Trial. BMJ Open.

[179] Formica M.B., Gianoudis J., Nowson C.A., O’Connell S.L., Milte C., Ellis K.A., Daly R.M. (2020). Effect of Lean Red Meat Combined with a Multicomponent Exercise Program on Muscle and Cognitive Function in Older Adults: A 6-Month Randomized Controlled Trial. Am. J. Clin. Nutr..

[180] Mischley L.K., Lau R.C., Bennett R.D. (2017). Role of Diet and Nutritional Supplements in Parkinson’s Disease Progression. Oxid. Med. Cell. Longev..

[181] Aragon A.A., Schoenfeld B.J. (2013). Nutrient Timing Revisited: Is There a Post-Exercise Anabolic Window?. J. Int. Soc. Sports Nutr..

[182] Takahashi Y., Matsunaga Y., Banjo M., Takahashi K., Sato Y., Seike K., Nakano S., Hatta H. (2019). Effects of Nutrient Intake Timing on Post-Exercise Glycogen Accumulation and Its Related Signaling Pathways in Mouse Skeletal Muscle. Nutrients.

[183] Ahalli S., Fort E., Bridai Y., Baborier N., Charbotel B. (2022). Mental Health and Working Constraints of First-Year PhD Students in Health and Science in a French University: A Cross-Sectional Study in the Context of Occupational Health Monitoring. BMJ Open.

[184] Wang L., Hu Y., Jiang N., Yetisen A.K. (2024). Biosensors for Psychiatric Biomarkers in Mental Health Monitoring. Biosens. Bioelectron..

[185] Bonato P. (2010). Wearable Sensors and Systems. IEEE Eng. Med. Biol. Mag..

[186] Masoumian Hosseini M., Masoumian Hosseini S.T., Qayumi K., Hosseinzadeh S., Sajadi Tabar S.S. (2023). Smartwatches in Healthcare Medicine: Assistance and Monitoring; a Scoping Review. BMC Med. Inform. Decis. Mak..

[187] Kang M., Chai K. (2022). Wearable Sensing Systems for Monitoring Mental Health. Sensors.

[188] Hickey B.A., Chalmers T., Newton P., Lin C.T., Sibbritt D., McLachlan C.S., Clifton-Bligh R., Morley J., Lal S. (2021). Smart Devices and Wearable Technologies to Detect and Monitor Mental Health Conditions and Stress: A Systematic Review. Sensors.

[189] Kim H., Kim Y.S., Mahmood M., Kwon S., Epps F., Rim Y.S., Yeo W.H. (2021). Wireless, Continuous Monitoring of Daily Stress and Management Practice via Soft Bioelectronics. Biosens. Bioelectron..

[190] Alshurafa N., Sideris C., Pourhomayoun M., Kalantarian H., Sarrafzadeh M., Eastwood J.A. (2017). Remote Health Monitoring Outcome Success Prediction Using Baseline and First Month Intervention Data. IEEE J. Biomed. Health Inform..

[191] Aranki D., Kurillo G., Yan P., Liebovitz D.M., Bajcsy R. (2016). Real-Time Tele-Monitoring of Patients with Chronic Heart-Failure Using a Smartphone: Lessons Learned. IEEE Trans. Affect. Comput..

[192] Dewa L.H., Lavelle M., Pickles K., Kalorkoti C., Jaques J., Pappa S., Aylin P. (2019). Young Adults’ Perceptions of Using Wearables, Social Media and Other Technologies to Detect Worsening Mental Health: A Qualitative Study. PLoS ONE.

[193] Osmani V. (2015). Smartphones in Mental Health: Detecting Depressive and Manic Episodes. IEEE Pervasive Comput..

[194] Behar J.A., Oster J., De Vos M., Clifford G.D. (2019). Wearables and MHealth in Mental Health and Neurological Disorders. Physiol. Meas..

[195] Reinertsen E., Osipov M., Liu C., Kane J.M., Petrides G., Clifford G.D. (2017). Continuous Assessment of Schizophrenia Using Heart Rate and Accelerometer Data. Physiol. Meas..

[196] Greco A., Benvenuti S.M., Gentili C., Palomba D., Scilingo E.P., Valenza G. (2018). Assessment of Linear and Nonlinear/Complex Heartbeat Dynamics in Subclinical Depression (Dysphoria). Physiol. Meas..

[197] Prince J., Arora S., De Vos M. (2018). Big Data in Parkinson’s Disease: Using Smartphones to Remotely Detect Longitudinal Disease Phenotypes. Physiol. Meas..

[198] Gomes N., Pato M., Lourenço A.R., Datia N. (2023). A Survey on Wearable Sensors for Mental Health Monitoring. Sensors.

[199] Robinson T., Condell J., Ramsey E., Leavey G. (2023). Self-Management of Subclinical Common Mental Health Disorders (Anxiety, Depression and Sleep Disorders) Using Wearable Devices. Int. J. Environ. Res. Public Health.

[200] Owens A.P. (2020). The Role of Heart Rate Variability in the Future of Remote Digital Biomarkers. Front. Neurosci..

[201] Kaushik A., Vasudev A., Arya S.K., Pasha S.K., Bhansali S. (2014). Recent Advances in Cortisol Sensing Technologies for Point-of-Care Application. Biosens. Bioelectron..

[202] Abd-Alrazaq A., AlSaad R., Aziz S., Ahmed A., Denecke K., Househ M., Farooq F., Sheikh J. (2023). Wearable Artificial Intelligence for Anxiety and Depression: Scoping Review. J. Med Internet Res..

[203] Jacobson N.C., Feng B. (2022). Digital Phenotyping of Generalized Anxiety Disorder: Using Artificial Intelligence to Accurately Predict Symptom Severity Using Wearable Sensors in Daily Life. Transl. Psychiatry.

[204] Meneses do Rêgo A.C., Araújo-Filho I. (2024). Leveraging Artificial Intelligence to Enhance the Quality of Life for Patients with Autism Spectrum Disorder: A Comprehensive Review. Eur. J. Clin. Med..

[205] Mone V., Shakhlo F. (2023). Health Data on the Go: Navigating Privacy Concerns with Wearable Technologies. Leg. Inf. Manag..

[206] Suryawanshi N.S. (2024). Predicting Mental Health Outcomes Using Wearable Device Data and Machine Learning. Int. J. Innov. Sci. Res. Technol..

[207] Canali S., Schiaffonati V., Aliverti A. (2022). Challenges and Recommendations for Wearable Devices in Digital Health: Data Quality, Interoperability, Health Equity, Fairness. PLoS Digit. Health.

[208] Novikov V.N., Badaeva A.V., Danilov A.B., Vorobyeva Y.D. (2023). Precision Neuronutrition: Personalized Approaches for Optimizing Brain Health. Biol. Life Sci. Forum.

[209] Bhatia D., Paul S., Acharjee T., Ramachairy S.S. (2024). Biosensors and Their Widespread Impact on Human Health. Sens. Int..

[210] Mishra A., Singh P.K., Chauhan N., Roy S., Tiwari A., Gupta S., Tiwari A., Patra S., Das T.R., Mishra P. (2024). Emergence of Integrated Biosensing-Enabled Digital Healthcare Devices. Sens. Diagn..

[211] Hernández-Mustieles M.A., Lima-Carmona Y.E., Pacheco-Ramírez M.A., Mendoza-Armenta A.A., Romero-Gómez J.E., Cruz-Gómez C.F., Rodríguez-Alvarado D.C., Arceo A., Cruz-Garza J.G., Ramírez-Moreno M.A. (2024). Wearable Biosensor Technology in Education: A Systematic Review. Sensors.

[212] Olyanasab A., Annabestani M. (2024). Leveraging Machine Learning for Personalized Wearable Biomedical Devices: A Review. J. Pers. Med..

[213] Kim D., Min J., Ko S.H. (2024). Recent Developments and Future Directions of Wearable Skin Biosignal Sensors. Adv. Sens. Res..

[214] Sidhu J.S., Jamwal A., Mehta D., Gautam A. (2024). Integration of IoT and AI in Bioengineering of Natural Materials. Calcium-Based Materials.

[215] Mahato K. (2024). Implantable Biosensors for Personalized Healthcare. Biosensors for Personalized Healthcare.

[216] Meng Z., Zhang Y., Yang L., Yuan F., Wang J., Chen J., Liu J., Wang G., Zang G. (2024). Application of Advanced Biosensors in Nervous System Diseases. Interdiscip. Med..

[217] Kussmann M., Fay L.B. (2008). Nutrigenomics and Personalized Nutrition: Science and Concept. Per. Med..

[218] Ebright B., Duro M.V., Chen K., Louie S., Yassine H.N. (2024). Effects of APOE4 on Omega-3 Brain Metabolism across the Lifespan. Trends Endocrinol. Metab..

[219] Lutz M., Moya P.R., Gallorio S., Ríos U., Arancibia M. (2024). Effects of Dietary Fiber, Phenolic Compounds, and Fatty Acids on Mental Health: Possible Interactions with Genetic and Epigenetic Aspects. Nutrients.

[220] Kaput J., Monteiro J.P. (2024). Human Nutrition Research in the Data Era: Results of 11 Reports on the Effects of a Multiple-Micronutrient-Intervention Study. Nutrients.

[221] Gáll Z., Székely O. (2021). Role of Vitamin D in Cognitive Dysfunction: New Molecular Concepts and Discrepancies between Animal and Human Findings. Nutrients.

[222] Aquili L. (2020). The Role of Tryptophan and Tyrosine in Executive Function and Reward Processing. Int. J. Tryptophan Res..

[223] Suganya K., Koo B.-S. (2020). Gut–Brain Axis: Role of Gut Microbiota on Neurological Disorders and How Probiotics/Prebiotics Beneficially Modulate Microbial and Immune Pathways to Improve Brain Functions. Int. J. Mol. Sci..

[224] Chudzik A., Orzyłowska A., Rola R., Stanisz G.J. (2021). Probiotics, Prebiotics and Postbiotics on Mitigation of Depression Symptoms: Modulation of the Brain–Gut–Microbiome Axis. Biomolecules.

[225] Lobo F., Haase J., Brandhorst S. (2022). The Effects of Dietary Interventions on Brain Aging and Neurological Diseases. Nutrients.

[226] Mohr A.E., Ortega-Santos C.P., Whisner C.M., Klein-Seetharaman J., Jasbi P. (2024). Navigating Challenges and Opportunities in Multi-Omics Integration for Personalized Healthcare. Biomedicines.

[227] Brancato V., Esposito G., Coppola L., Cavaliere C., Mirabelli P., Scapicchio C., Borgheresi R., Neri E., Salvatore M., Aiello M. (2024). Standardizing Digital Biobanks: Integrating Imaging, Genomic, and Clinical Data for Precision Medicine. J. Transl. Med..

[228] Chbeir S., Carrión V. (2023). Resilience by Design: How Nature, Nurture, Environment, and Microbiome Mitigate Stress and Allostatic Load. World J. Psychiatry.

[229] Hantsoo L., Jagodnik K.M., Novick A.M., Baweja R., di Scalea T.L., Ozerdem A., McGlade E.C., Simeonova D.I., Dekel S., Kornfield S.L. (2023). The Role of the Hypothalamic-Pituitary-Adrenal Axis in Depression across the Female Reproductive Lifecycle: Current Knowledge and Future Directions. Front. Endocrinol..

[230] Lapp H.E., Ahmed S., Moore C.L., Hunter R.G. (2019). Toxic Stress History and Hypothalamic-Pituitary-Adrenal Axis Function in a Social Stress Task: Genetic and Epigenetic Factors. Neurotoxicol. Teratol..

[231] Nowacka-Chmielewska M., Grabowska K., Grabowski M., Meybohm P., Burek M., Małecki A. (2022). Running from Stress: Neurobiological Mechanisms of Exercise-Induced Stress Resilience. Int. J. Mol. Sci..

[232] Ignácio Z.M., da Silva R.S., Plissari M.E., Quevedo J., Réus G.Z. (2019). Physical Exercise and Neuroinflammation in Major Depressive Disorder. Mol. Neurobiol..

[233] Numakawa T., Kajihara R. (2023). Involvement of Brain-Derived Neurotrophic Factor Signaling in the Pathogenesis of Stress-Related Brain Diseases. Front. Mol. Neurosci..

[234] Zühtü Birinci Y. (2023). The Potential Role of Exercise-Induced Neurotrophic Factors for Mental Health. Mental Health—Preventive Strategies.

[235] Molina-Hidalgo C., Stillman C.M., Collins A.M., Velazquez-Diaz D., Ripperger H.S., Drake J.A., Gianaros P.J., Marsland A.L., Erickson K.I. (2023). Changes in Stress Pathways as a Possible Mechanism of Aerobic Exercise Training on Brain Health: A Scoping Review of Existing Studies. Front. Physiol..

[236] Fiocco A.J., D’Amico D., De Beaumont L., Poirier J., Lupien S. (2020). Association between BDNF Polymorphism and Hypothalamic-Pituitary-Adrenal Activity in Later Adulthood. Gerontology.

[237] Tsutsumi T., Nakano D., Hashida R., Sano T., Kawaguchi M., Amano K., Kawaguchi T. (2023). The Inter-Organ Crosstalk Reveals an Inevitable Link between MAFLD and Extrahepatic Diseases. Nutrients.

[238] Estrada J.A., Contreras I. (2019). Nutritional Modulation of Immune and Central Nervous System Homeostasis: The Role of Diet in Development of Neuroinflammation and Neurological Disease. Nutrients.

[239] Calderón-Ospina C.A., Nava-Mesa M.O. (2020). B Vitamins in the Nervous System: Current Knowledge of the Biochemical Modes of Action and Synergies of Thiamine, Pyridoxine, and Cobalamin. CNS Neurosci. Ther..

[240] Holton K. (2023). The Potential Role of Dietary Intervention for the Treatment of Neuroinflammation. Translational Neuroimmunology: Neuroinflammation.

[241] Benmelouka A.Y., Shah M.A., Saleem U., Elshanbary A.A., Meshref M., Shah G.M., Alsharif I., Althobaiti N.A., Alhasani R.H. (2022). Therapeutic Role of Nutraceuticals in the Management of Brain Disorders. The Role of Phytonutrients in Metabolic Disorders.

[242] Donati Zeppa S., Ferrini F., Agostini D., Amatori S., Barbieri E., Piccoli G., Sestili P., Stocchi V. (2022). Nutraceuticals and Physical Activity as Antidepressants: The Central Role of the Gut Microbiota. Antioxidants.

[243] Liaqat H., Parveen A., Kim S.Y. (2022). Antidepressive Effect of Natural Products and Their Derivatives Targeting BDNF-TrkB in Gut–Brain Axis. Int. J. Mol. Sci..

[244] O’Riordan K.J., Collins M.K., Moloney G.M., Knox E.G., Aburto M.R., Fülling C., Morley S.J., Clarke G., Schellekens H., Cryan J.F. (2022). Short Chain Fatty Acids: Microbial Metabolites for Gut-Brain Axis Signalling. Mol. Cell Endocrinol..

[245] Gubert C., Kong G., Renoir T., Hannan A.J. (2020). Exercise, Diet and Stress as Modulators of Gut Microbiota: Implications for Neurodegenerative Diseases. Neurobiol. Dis..

[246] Belbin O., Lehmann S., Sabidó E., Hirtz C. (2020). Editorial: Proteomics as a Tool for Biomarker and Drug Target Discovery: Improving the Diagnosis and Treatment of Neurodegenerative Diseases. Front. Aging Neurosci..

[247] Akselrod S., Collins T.E., Hoe C., Seyer J., Tulenko K., Ortenzi F., Berlina D., Sobel H. (2023). Building an Interdisciplinary Workforce for Prevention and Control of Non-Communicable Diseases: The Role of e-Learning. BMJ.

[248] Koníčková D., Menšíková K., Tučková L., Hényková E., Strnad M., Friedecký D., Stejskal D., Matěj R., Kaňovský P. (2022). Biomarkers of Neurodegenerative Diseases: Biology, Taxonomy, Clinical Relevance, and Current Research Status. Biomedicines.

[249] Park S.A., Jang Y.J., Kim M.K., Lee S.M., Moon S.Y. (2022). Promising Blood Biomarkers for Clinical Use in Alzheimer’s Disease: A Focused Update. J. Clin. Neurol..

[250] Drzezga A., Barthel H., Minoshima S., Sabri O. (2014). Potential Clinical Applications of PET/MR Imaging in Neurodegenerative Diseases. J. Nucl. Med..

[251] Lamptey R.N.L., Chaulagain B., Trivedi R., Gothwal A., Layek B., Singh J. (2022). A Review of the Common Neurodegenerative Disorders: Current Therapeutic Approaches and the Potential Role of Nanotherapeutics. Int. J. Mol. Sci..

[252] Parlak O., Keene S.T., Marais A., Curto V.F., Salleo A. (2018). Molecularly Selective Nanoporous Membrane-Based Wearable Organic Electrochemical Device for Noninvasive Cortisol Sensing. Sci. Adv..

[253] Watson C.N., Belli A., Di Pietro V. (2019). Small Non-Coding RNAs: New Class of Biomarkers and Potential Therapeutic Targets in Neurodegenerative Disease. Front. Genet..

